# Biofouling of Polyamide Membranes: Fouling Mechanisms, Current Mitigation and Cleaning Strategies, and Future Prospects

**DOI:** 10.3390/membranes9090111

**Published:** 2019-08-30

**Authors:** Jane Kucera

**Affiliations:** Nalco Water, An Ecolab Company, 1601 West Diehl Road, Naperville, IL 60563, USA; jkucera@ecolab.com; Tel.: +1-815-258-6418

**Keywords:** reverse osmosis, polyamide, membranes, biofouling, biocides, chlorine, chlorine dioxide, graphene oxide, cleaning

## Abstract

Reverse osmosis and nanofiltration systems are continuously challenged with biofouling of polyamide membranes that are used almost exclusively for these desalination techniques. Traditionally, pretreatment and reactive membrane cleanings are employed as biofouling control methods. This in-depth review paper discusses the mechanisms of membrane biofouling and effects on performance. Current industrial disinfection techniques are reviewed, including chlorine and other chemical and non-chemical alternatives to chlorine. Operational techniques such as reactive membrane cleaning are also covered. Based on this review, there are three suggested areas of additional research offering promising, polyamide membrane-targeted biofouling minimization that are discussed. One area is membrane modification. Modification using surface coatings with inclusion of various nanoparticles, and graphene oxide within the polymer or membrane matrix, are covered. This work is in the infancy stage and shows promise for minimizing the contributions of current membranes themselves in promoting biofouling, as well as creating oxidant-resistant membranes. Another area of suggested research is chemical disinfectants for possible application directly on the membrane. Likely disinfectants discussed herein include nitric oxide donor compounds, dichloroisocyanurate, and chlorine dioxide. Finally, proactive cleaning, which aims to control the extent of biofouling by cleaning before it negatively affects membrane performance, shows potential for low- to middle-risk systems.

## 1. Introduction

Since its beginnings in the early 1960s, membrane-based desalination has become a leading technology in municipal and industrial settings, currently outpacing traditional thermal technologies [1]. As of June 2018, membrane-based desalination accounted for just under 2.5 million m^3^/day of annual contracted capacity, while thermal technologies accounted for less than 0.5 m^3^/day annual contracted capacity [2]. Reverse osmosis (RO) dominates membrane-based desalination techniques, which also include electrodialysis, electrodialysis reversal, forward osmosis, nanofiltration (NF), and continuous electrodeionization. Efficiency advancements in RO resulting in higher flux and higher selectivity membranes that require less energy for desalination, plus the use of energy-recovery devices, have allowed RO to surpass thermal technologies for desalination.

When first developed, RO relied on membranes made of cellulose acetate (CA). These membranes suffered from hydrolysis at pH outside the range of 4–6, low rejection, especially of silica, and high operating pressures. The membrane virtually all RO systems use for desalination today is based on polyamide chemistry [3]. Developed by John Cadotte in the 1970s and early 1980s [4,5], the polyamide membrane is actually a composite composed of polyamide formed *in-situ* on a microporous polysulfone support using interfacial polymerization. The resultant membrane has a thin, polyamide barrier layer supported on an open polysulfone structure capable of providing support for the polyamide layer and channels for permeate water to pass through unhindered. 

While polyamide membranes offer advantages over CA membranes in terms of rejection, operating pressure, and wider operating pH range, there are limitations to these polyamide membranes. Perhaps the most problematic issues for users of polyamide membranes is controlling deposition of foulants, in particular, microorganisms, on the membranes [6,7,8,9,10,11]. Biofouling leads to performance (flux and rejection) losses, and corresponding shorter useful membrane life [9,12]. The major limitation is the sensitivity of polyamide to oxidizers, making biofouling control directly on the membrane itself challenging. Mitigation strategies for biofouling require pretreatment, cleaning, and/or direct membrane treatment (currently using non-oxidizing biocides), as well as adjustments in operations to minimize effects on overall plant performance. 

**Figure 1:**
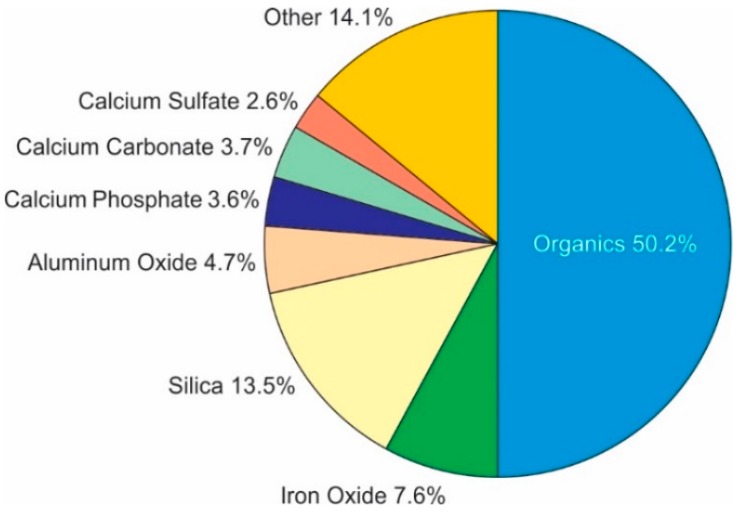
Average foulant distribution, excluding biofouling, from one hundred fifty worldwide membrane autopsies. All membranes showed at least some biofouling and for 33% of the membranes, the degree of biofouling was enough to be the primary mode of performance failure [13]. Used with permission of the Engineers’ Society of Western Pennsylvania.

To understand specific pretreatment and operational techniques required to minimize performance loses due to biofouling, one must first understand how serious biofouling of polyamide membranes is. The results of a study by Fazel [13] of 150 failed polyamide membranes which were autopsied are shown in Figure 1. The primary foulant was organic in nature, representing about half of the materials found on the membranes; inorganic scale and particulates represented the balance of materials found on the membranes. This study also found the membranes were fouled with microorganisms [13], and that the biofouling was endemic; virtually all the membranes autopsied had base-loaded biocounts of at least 10^2^–10^4^ colony forming units (CFU)/cm^2^. Further, this base loading of microorganisms was found to be irrespective of the pretreatment used or the nature of the feed water [13]. Roughly 33% of the membranes autopsied had high colony counts (> 10^5^ CFU/cm^2^) such that biofouling was a major contributor to the decline in membrane performance. Conclusions from Fazel’s [13] research show that 33% of membranes with performance loses were shown to have biofouling as the primary factor affecting performance, and another 33% of membranes had biofouling as a contributing factor. Komlenic [14] found that 45% of all membrane fouling included biofouling as a contributing factor to performance decline. Pena et al. [9] studied five hundred autopsied membranes and found that 31.3% had biofouling as the primary fouling component contributing to membrane performance decline. A study by Amjad [15] found that biofouling together with organic and particulate/colloidal fouling have synergistic mechanisms. Even when the potential for fouling with microorganisms alone is relatively low, the synergistic effects of the presence of organics and particulates in feed water, enhance the potential for all types of membrane fouling and scaling, including biofouling [15]. Indeed, the study by Pena et al. [9] found that the most prevalent, secondary component to biofouled membranes was colloidal/particulate fouling. And, work by Weinrich et al. [16] concluded that organic matter, particularly the biodegradable fraction, is also a key contributor to membrane biofouling. 

The study by Fazel [13] showed the fouling order, highest potential to lowest potential, was brackish surface water, followed by seawater, tap (municipal) water, and well water. The use of membranes for wastewater applications was not as prevalent at the time of this study as it is today (and will be in the future), so none of the membranes tested by Fazel had been employed in this manner. However, due to the nature of wastewaters, it can be inferred that wastewater recycle/reuse applications may have a greater biofouling potential than brackish surface water. Work by Xu et al. [17] demonstrated that polyamide membranes operating on municipal wastewater-reclamation pilot systems exhibited biofouling as the primary foulant and that biofouling was found in all elements in the system, including the tail elements. As polyamide membranes are tasked with treating ever more challenging feed sources, biofouling will become even more significant for these membranes [18]. 

Biofouling control/prevention remains the most difficult challenge facing polyamide membrane desalination operations, despite advances in membrane performance over the last 45 years [19,20]. Many membrane biofouling control technologies and strategies have been developed over time [6,21,22,23,24,25] and new approaches are being employed to overcome the shortcomings of current methods. In general, current biofouling control programs consist of appropriate pretreatment and good membrane cleaning practices [21,26,27]. This paper includes a primer on membrane biofouling (Section 2), a review of methods to control biofouling via traditional pretreatment and cleaning techniques (Section 3 and Section 4, respectively), and information on innovative techniques that may improve upon current results (Section 5). Discussion and conclusions are presented in Section 6 and Section 7, respectively.

## 2. Biofouling of Polyamide Membranes

### 2.1. What Is Membrane Biofouling

Membrane biofouling can be the result of aerobic bacteria, such as those found in brackish and seawater systems, as well as wastewater, and/or anaerobic bacteria in ground water systems, such as iron- and sulfate-reducing bacteria. Further, membrane biofouling can occur rapidly. Bereschenko et al. [28] found that a mature biofilm structure can form in a little as 1 month on a polyamide membrane. In their study, they discovered that a 1-month old biofilm on a polyamide membrane featured a structure similar to that found on a 5-year old polyamide membrane operating in the same plant with surface water feed (pretreatment included coagulation, flocculation, sand filtration, ultrafiltration, and cartridge filtration). Hence, it is imperative that biofouling control strategies be implemented at membrane start up and maintained throughout the life of operations. 

Biofouling begins with the formation of a conditioning film. A conditioning film is comprised of adsorbed organic and suspended materials that collect on the surface of the membrane [6]. Organic compounds that will promote the growth of microorganisms include carboxylic and amino acids, proteins, and carbohydrates. Concentrations as low as parts per billion (ppb) of these organic compounds can lead to significant biofouling-based clogging of spiral-wound element feed channels [29]. Higher water flux through the membrane speeds the rate of transport of these compounds to the membrane surface, thereby increasing the rate of accumulation in the concentration polarization layer, thus favoring the formation of the conditioning film. At this point is biofouling development, microorganisms are still reversibly attached to the membrane [30]. 

The next steps in membrane biofouling are adhesion of the bacteria and cell growth with microcolony formation [6,31]. Many factors contribute to adhesion, and bio-adhesion can become permeant within hours [32]. The rate at which microorganisms transport to, and accumulate on, the membrane is a critical factor in adhesion [33]. Another critical factor is concentration of microorganisms in the system feed water [34], where high concentrations can increase the rate of microorganism accumulation on the membrane surface [35]. Higher concentrations of microorganisms coupled with quicker transport to the membrane surface (as a function of water flux) can serve to exacerbate the accumulation of microorganisms and the rate of bacterial adhesion. Adhesion enables bacterial cell growth and the formation of microcolonies. 

Extracellular polymeric substances (EPS) are formed as growth and the formation of biofilm matures [32]. EPS consist primarily of polysaccharides, proteins, glycoproteins, and lipoproteins. EPS serve to protect the bacteria from biocides, flow shear, and predators. About 90% of the resultant biofouling structure is composed of EPS, with the remaining 10% microorganisms [36]. 

The final step of membrane biofouling is the plateau phase. This is an equilibrium phase where attachment is essentially in equilibrium with detachment that occurs due to fluid shear forces [37,38]. In a membrane module and in the membrane system itself, this phase is critical to the proliferation of bacteria and resultant biofouling further along the feed channel, thereby expanding the degree of membrane surface area infected up to including the entire system.

### 2.2. Membrane Specific and Situational Factors that Affect Membrane Biofouling

#### 2.2.1. Polyamide Membrane Characteristics

**Figure 2:**
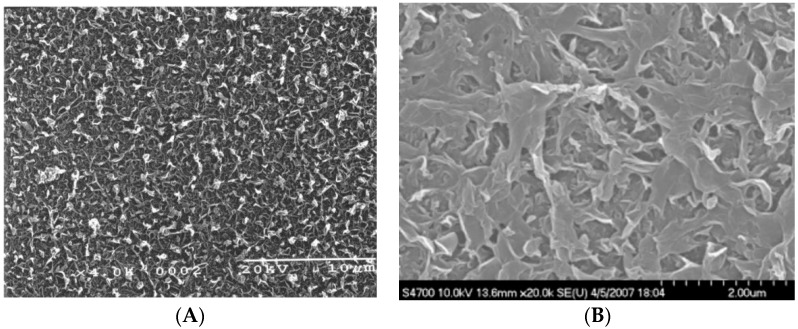
**Scanning electron micrograph** images demonstrating. surface roughness of polyamide membranes. (**A**) scale = 10 microns; courtesy of Mark Wilf. (**B**) scale = 2 microns; courtesy of Eric M.V. Hoek (reprinted with permission of Elsevier B.V.) [40].

**Figure 3:**
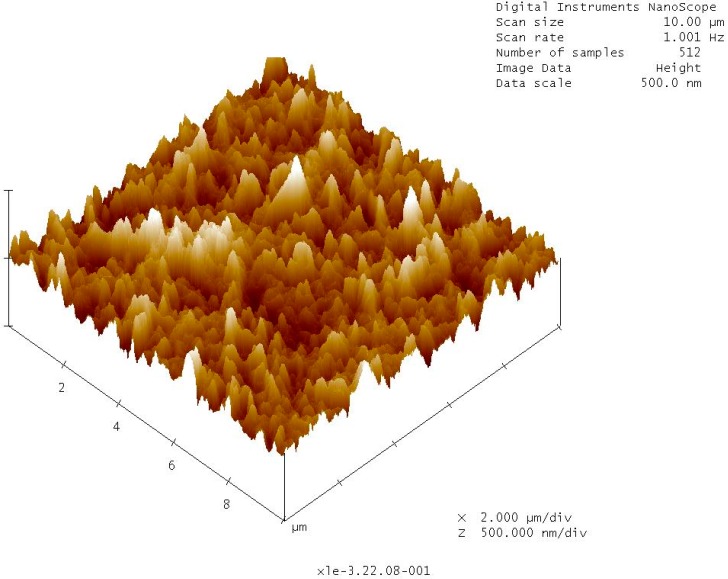
Atomic force microscopy (AFM) image of a polyamide membrane showing representative roughness. Courtesy of Eric M.V. Hoek (reprinted with the permission of Elsevier B.V.) [40].

The membrane developed by Cadotte exhibits superior performance for desalination while suffering some attributes which can enhance to microbial fouling. There are three major membrane characteristics that influence bacterial adhesion and subsequent biofouling: membrane surface roughness, membrane surface/solution interface zeta potential, and hydrophilicity. Rough surfaces promote collection and adhesion of microbes [34,39]. Polyamide membranes are characterized as having a very rough surface (see Figure 2 and Figure 3) which offers enhanced surface area for microbial adhesion and accumulation of organic nutrients. The rough structure also shelters bacteria and organic nutrients from sheer forces applied to sweep these and other foulants away from the membrane surface.

Understanding the effect of surface roughness on biofouling potential is straight forward; membrane charge and hydrophilicity effects are more complex, as they are affected by feed water characteristics (e.g., pH and ionic strength of the feed solution) and by the specific nuances in membrane chemistry among various makes and models. Polyamide membranes tend to be hydrophilic (as measured by contact angle, generally 90° or less [40,41,42]), although there are variations among membrane models and types. Lee et al. [41] tested five different, commercially available membranes for dynamic contact angle and found that the angles ranged from 33.0° ± 0.4° to 91.0° ± 0.4° (pH and molar concentration were not provided). Hurwitz et al. [40] tested the static contact angle of one membrane at various feed water conditions. They demonstrated that higher feed water pH (at constant ionic strength) and ionic strength (at constant pH), resulted in lower contact angles, greater wettability (as determined by the interfacial free energy using the Young-Depré equation (which employs contact angle)) and greater hydrophilicity (as described by the free energy of cohesion (interfacial free energy per unit area), where a negative value is indicative of non-cohesiveness and a hydrophilic material when immersed in water) [40]. Table 1 and Table 2 show these results, as reported by Hurwitz et al. [40]. The hydrophilic nature of the membrane and its non-cohesiveness, which correlate with a smaller contact angle, imply that the membrane interacts with water rather than colloids and bacteria, leading to decreasing tendency to foul with microbes at higher pH [23]. 

**Figure 4:**
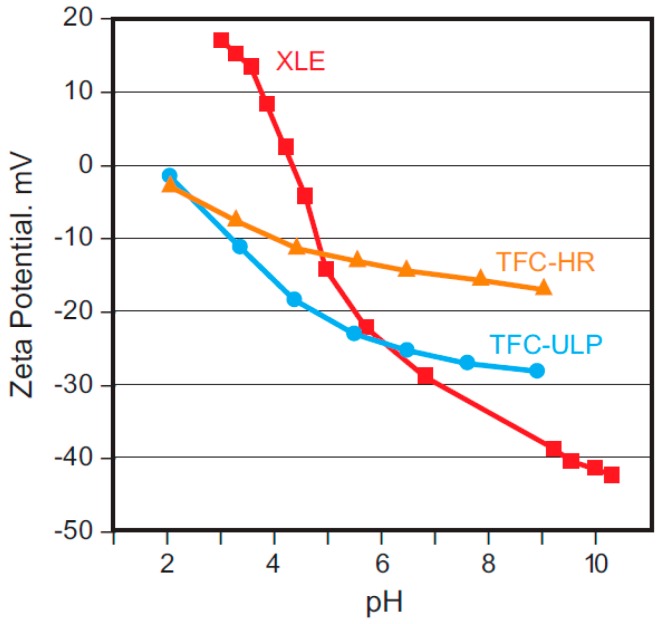
Zeta potentials for three polyamide RO membranes as a function of pH when immersed in a 10 mM solution of sodium chloride. Membranes tested include a high-rejection type (thin composite (TFC)-HR, Koch Membrane Systems, Wilmington, MA, USA), a low-energy type (XLE, DuPont-FilmTec), and an ultra-low-pressure type (TFC-ULP, Koch Membrane Systems). XLE membrane tested at 25 °C (temperature for other membranes tested was not reported). Used with Permission from Elsevier B.V. [40,44].

Membrane surface charge is typically described by streaming potential (usually tested via electro-kinetic methods, and using the Grahame, Helmholtz-Smoluchowski, Fair-Mastin and/or Gouy-Chapman equations, which relate the charge at the membrane-solution interface directly to the streaming (zeta) potential). [40,43,44,45]. As with contact angle, zeta potential for polyamide membranes depends on the specific type of polyamide membrane (e.g., low-energy (typically higher specific flux), high-rejection (typically lower specific flux), etc.), pH, and membrane feed solution chemistry [40,44]. Figure 4 shows the zeta potential for three different membranes, high-rejection, low-energy, and ultra-low energy, as a function of pH for membranes exposed to a 10 mM solution of sodium chloride [40,44]. All three membranes show greater negative zeta potential with increasing pH, suggesting that the membrane surface functional groups (such as carboxylic acid (RCOOH)) become negatively ionized with increasing pH [45,46]. The low-energy membranes, which typically exhibit higher specific flux (an indication of greater hydrophilicity), also exhibited greater negative charge with increasing pH than the “tighter,” high-rejection membrane. Bacteria have a negative surface charge, so they are more repulsed by the membrane at negative zeta potential, e.g., at higher operating pH (Table 1). 

From the discussions above, the zeta potential and hydrophilic nature discourages bio-adhesion to some degree, with pH being a key factor. At the pH of most operating RO systems (7–9), contact angles range from approximately 55° down to 47° and zeta potentials that range from −5 down to −35 over the same pH range. Although the characteristics are described individually, they do act synergistically in the development of membrane biofouling [23]. While membrane surface characteristics affect biofouling to varying degrees, they were shown to be relatively minor contributors to bacterial adhesion and biofilm formation when compared to characteristics of the bacteria themselves [47]. The presence of external bacterial appendages, such as flagella, and the EPS the bacteria produce were found to be the primary factors in the development of colony proliferation [48]. Nevertheless, research into membrane modifications to minimize the degree to which surface characteristics affect bacterial adhesion continues.

#### 2.2.2. Situational Factors 

Situational factors that promote adhesion and biofouling involve primarily the characteristics of the membrane feed water and the hydrodynamic conditions within the spiral-wound membrane element used in most industrial desalination systems. The moist and relatively low shear environment within an element promote proliferation of bacteria and biofilm on the membranes and on the element’s materials of construction (i.e., the feed channel spacer). As with the membrane characteristics described previously, situational factors work synergistically with each other and also with membrane characteristics.

##### Feed Water Characteristics

Several feed water characteristics have been shown to influence biofouling of membranes, including concentration of microorganisms and nutrients, ionic strength, pH, and pretreatment chemicals [6,37,49]. The concentration polarization layer plays a key role, as the concentration of microorganisms and nutrients in the layer will directly affect rates and quality of adhesion and the formation of the ultimate biofilm [23,30,35,50]. Higher concentration of microbes will increase the rate of biofouling [23]. Increasing the carbon concentration in the bulk solution can also lead to higher rates of biofilm formation and an increase in the mass of microorganisms present in the biomass [51]. Higher ionic strength and acidic pH both enhance bacterial adhesion [6,52,53,54,55], presumably by altering the membrane surface-solution interfacial characteristics, as described previously. 

Another feed water characteristic to consider is whether feed water chemicals, such as antiscalants, are being used [56]. Antiscalant are usually used in industrial and municipal desalination systems. A study by Sweity et al. [49] found that the use of polyacrylate- and polyphosphonate-based RO antiscalants can enhance membrane biofouling. In bench, pilot, and on-line tests at an operating desalination plant, they found that polyacrylate-based antiscalants altered the physio-chemical nature of the polyamide membrane by primarily enhancing hydrophobicity; this promoted initial attachment/deposition of microbial cells. Polyphosphonate-based antiscalants sustained biomass on the membrane by providing nutrients in the form of phosphorous [49]. As noted by Sweity et al. [49] in their work, there is very limited, quantitative data regarding antiscalants and their contribution to membrane biofouling in the literature. Two other works are cited by Sweity et al. [49] (another study by Sweity et al. [57], which confirms these findings, and Vrouwenvelder et al. [58]) along with a study by Ashfaq et al. [56] all go on to say that vast array of antiscalants available can promote biofouling but vary significantly in their ability to promote biofouling on polyamide membranes. Polymer-based antiscalants were shown to promote more biofouling than other types [56]. Assimilable organic carbon (AOC), biofilm formation rate (BFR), and biomass production potential (BPP) tests can determine the biofouling potential of a specific antiscalant [58]. Based on these studies, the effect of antiscalants on polyamide membrane biofouling should be researched further and considered when such products are employed.

##### Hydrodynamic Conditions within the Spiral-Wound Membrane Element

Convective flux to the membrane and diffusive flows near the membrane bring organics and microorganisms to the membrane surface [33,37]. The lack of convective flow in the axial direction near the membrane surface allows the organics and microorganisms to settle in the concentration polarization layer. The thicker the concentration polarization, the greater the potential for biofouling to occur [30]. Boundary layer thickness is function of the crossflow velocity of the bulk solution flowing through the membrane element. The degree to which concentration is polarized is a function of the water flux through the membrane. Lower crossflow velocities enhancing the boundary layer thickness and high water flux increases the rate at which material accumulates in the boundary layer. 

### 2.3. Characterization of Membrane Biofouling

**Figure 5:**
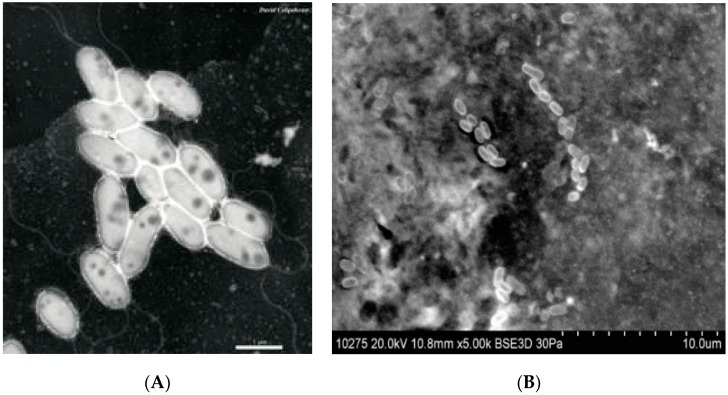
*Sphingomonas* (**A**) and other, rod-shaped bacteria on the surface of an autopsied membrane (**B**). (**A**) scale = 1 micron, (**B**) scale = 10 microns.

A review by Sanchez [20] found that bacterial populations on polyamide membranes are diverse, and depend on several factors, including nutrient availability and feed water pretreatment techniques. Several studies report that the predominant genus found in virtually all RO membrane biofilms is *Sphingomonas* [28,59,60,61,62]. (*Sphingomonas* are Gram-negative, rod-shaped, aerobic bacteria as shown in Figure 5). *Sphingomonas* also appear to be the foundational biofouling organisms. *Sphingomonas* produce unique extracellular polysaccharides that build and maintain the biofilm [63] and protect the biofilm matrix against attack from cleaning chemicals [39]. Further, the EPS they produce provides a modified membrane surface to which other microorganisms can readily attach and proliferate [20,28]. These bacteria can consume a broad range of naturally occurring organic compounds and survive in the high-nutrient environment found in the concentration polarization layer [59]. *Sphingomonas* have adapted well to the conditions within the spiral wound membrane element [59], where they survive and proliferate in a low-carbon and low-oxygen environment [39,64]. While *Sphingomonas* made up over 26% of the total bacterial counts on membranes autopsied by Bereschenko et al. [59], other varieties found include *Planctomycetes* (14.5% of the total biocount), *Lysobacter* (6.0% of the total biocount), and *Nitrosomonas* (4.6% of the total biocount). *Pseudomonas*, known for its metabolic diversity and ability to grow in a wide variety of environments [65], was found to be a minor component (2.0%) of the total bacterial population [59]. 

### 2.4. Measurement of Biofouling

There are many techniques to measure bacteria and biofouling in membrane systems, as outlined by Al-Juboori [32]. These include various microscopic and spectroscopic techniques, and more esoteric methods such as fiber optical sensors, differential heat transfer, and metabolic product measurements. The most common techniques typically employed by industrial membrane systems include AOC, adenosine triphosphate (ATP), total direct counts (TDC), and heterotrophic plate counts (HPC). 

#### 2.4.1. Assimilable Organic Carbon (AOC)

AOC is a laboratory bioassay technique used to measure the potential for heterotrophic bacteria to grow in water [66,67,68,69,70]. AOC is the small fraction (0.1–9.0%) [71] of total organic carbon (TOC) used by specific strains of bacteria (*Pseudomonas fluorenscens* P17 and *Spirillum* NOX) to increase biomass. AOC is generally composed of low molecular weight compounds, such as aldehydes, ketones, and carboxylic acids, which are most readily assimilated by the bacteria [68,72]. 

AOC testing can be used to determine the potential for heterotrophic growth in any system, but it is used primarily in municipal systems for determining regrowth potential in distribution systems following primary disinfection. To predict the potential for biofouling of polyamide membranes, studies by Vrouwenvelde and van der Kooij [66,73] showed that AOC values in feed water can range from 3 to as high as 1500 ppb acetate-C, and that values as low as 3 ppb acetate-C resulted in biofouling of membranes [73]. 

The amount of AOC present can be reduced using biological activated carbon (BAC) plus ozone or oxygen [72,74,75]. The objective is to remove nutrients via the BAC bed so that AOC available within the downstream membrane system is reduced, thereby inhibiting the growth of bacteria on the membranes. Ozone is used to break down larger organics into smaller AOC, [6,76,77] ensuring maximum availability of carbon-based nutrients for assimilation by the bacteria in the BAC bed. A study by van der Mass [75], demonstrated good RO membrane biofouling control using BAC with oxygen. However, the use of carbon beds is not desirable prior to a membrane system due to the tendency for microorganisms to slough off carbon media into downstream piping and equipment, resulting in inoculation of the membranes with microbes [78,79,80,81]. 

#### 2.4.2. Adenosine Triphosphate (ATP)

ATP is a molecule associated only with living organisms including bacteria, algae, protozoa, and fungi, and is used to provide energy for cellular metabolism and enzymatic reactions [82]. Studies by Vrouwenvelder and van der Kooij [73] showed that the ATP for feed water to membrane-based desalination facilities range from less than 1 ppt ATP to as high at 200 ppt ATP. However, direct measurement of ATP does not give a clear picture of the potential for actual biofouling of the membranes themselves. Instead, a biofilm formation rate (BFR) test has developed to measure the ATP that accumulates on a substrate over time to predict RO and NF membrane biofouling [73]. Vrouwenvelder and van der Kooij [73] demonstrated that membranes with feed water BFR of greater than 120 pico-grams ATP/cm^2^-day exhibited severe biofouling, and membranes exposed to feed water with a BFR of less than 1 pico-gram ATP/cm^2^-day experienced stable performance with minimal (2-year-interval) cleaning. 

#### 2.4.3. Heterotrophic Plate Counts (HPC)

HPC (also known as indirect viable cell counts) count active colonies in a sample and is commonly used in industrial settings. HPC field testing simply involves dipping a coupon into the solution to be tested and incubating the sample. Operators of membrane systems typically use HPC to determine the *relative* condition of a membrane with respect to microbial fouling that has already occurred rather than for determining the potential for biofouling. The degree of membrane biofouling is typically determined by monitoring the HPC of membrane system concentrate stream. The study of one hundred fifty RO membrane autopsies by Fazel [13] described previously found that counts of 10,000 CFU/cm^2^ on an RO membrane was representative of problematic biofouling. Since HPC values have been shown to represent a little at 10% of the total direct cell count on a membrane [73], it is generally accepted that HPC of 1000 CFU/mL in the concentrate is indicative of problematic membrane biofouling [83]. The HPC technique is more valuable for establishing trends in the degree of biofouling that is present rather than determining absolute number of microorganisms present [73].

#### 2.4.4. Total Direct Counts (TDC)

TDC (also known as microscopic cell counts) count both active and non-active cells. Vrouwenvelder and van der Kooij [73] found that TDC in feed water to membrane-based desalination facilities ranged from less than 400 up to 5 × 10^7^ cells /mL. No correlations were made with the degree of biofouling these counts represented. While TDC testing is faster and more accurate than HPC testing, TDC technique is not well suited for field testing due to the need for microscopy. 

### 2.5. Effects of Biofouling on Membrane Desalination Systems

Bacterial fouling in a membrane system leads to several deleterious effects on performance. These effects include loss of permeate quality and flow, as well as increases in operating energy. This costs operators labor, time, and materials via increased cleaning and shorter membrane life. 

#### 2.5.1. Scale Formation and Salt Passage

Concentration polarization is enhanced within the biofilm allowing for saturation and supersaturation of salts to occur within the biofilm causing scale to form [84,85]. Scaling within the biofilm matrix present problems in cleaning of the membrane, as high pH is used for biofouling and low pH is typically used for calcium-based scale removal. Uneven colony formations on the membrane surface yield areas between formations where crossflow velocity can be slow or non-existent. Biofilm covering these uneven formations increases the surface area for further scale nucleation to occur [86]. Localized scaling is also enhanced due to the uneven colony growth and the resultant areas of lower to non-existent crossflow velocity [6]. Scaling begins as a surface phenomenon and, therefore, typically begins without a noticeable increase in differential pressure, especially in spiral wound elements [87]. 

#### 2.5.2. Hydrodynamic Effects

Formation and proliferation of the biofilm matrix on the membrane surface and on the element feed spacer results in a decrease in water flux and an increase in differential pressure [59,87], The decrease in flux reduces the productivity of the system. Flux decline was found to be synergistically exacerbated by enhanced scaling due to biofouling, resulting in a total decline that was higher than predicted for sum of biofouling and scaling individually [85]. Operating pressure for a system is usually increased to compensate for loss of flux due to biofouling and scaling to maintain design product flow rate [6]. Additionally, accumulation of bacteria, viable and non-viable, on the feed channel spacer within the membrane element results in an increase in differential pressure through the system, and the increase in differential pressure can be observed within days of inoculation of the membranes with microbes. 

## 3. Techniques to Address Membrane Biofouling

The objectives of any biofouling technique are to kill or disable microbes to prevent infecting the membranes [37]. Additionally, living microbes and nutrients (including dead microbial bodies) within the membrane module or on the membranes themselves need to be removed [37]. There are several methods that attempt to meet these objectives. These methods include membrane modification, disinfection, membrane cleaning, and modification of the bacterium or organic nutrient (modification to the bacterium or nutrients are beyond this scope of this paper and are not covered here). Membrane modification serves to improve the characteristics of the membrane itself to resist biofouling. Disinfection of a membrane system involves reduction of the number of viable microorganisms present. Disinfection of water systems is usually accomplished via chemical, non-chemical, or thermal processes (thermal techniques cannot be used with standard polyamide membranes because their high-temperature limit is 45 °C; heat sterilization temperatures required for thermal deactivation of bacteria are at a minimum of 121 °C, hence, thermal techniques are not covered in this work.) Due to the sensitivity of polyamide membranes to oxidizing chemicals, disinfection via chemical means is limited to the pretreatment portion of the system. And, membrane cleaning, generally used after-the-fact to remove accumulated biomass, is limited to *removal* of biofouling rather than actual disinfection of the membrane itself. 

### 3.1. Disinfection Techniques 

Traditional disinfection techniques include chemical biocide and non-chemical or physical processes. Biocides include oxidizers, which cannot be used directly on polyamide membranes, and non-oxidizers, which can be used on membranes, but are not as effective as oxidizers. Physical techniques, such as ultraviolet (UV) radiation can be effective but suffer from a lack of residual disinfection capability.

#### 3.1.1. Chemical Biocides

There are numerous biocides that have been used or considered for use for disinfection of membranes systems. Chlorine is perhaps the most commonly used biocide. The availability and ease of application as well as chlorine’s ability to quickly deactivate most microorganisms make this chemical attractive for membrane treatment [50]. However, formation of trihalomethanes (THMs) and haloacetic acids (HAAs) have led to other biocides being used or considered for use by many facilities. Further, sensitivity of polyamide to destruction by chlorine, has also spurred the search for alternative chemical biocides. Chloramine is typically considered as an alternative to chlorine. Other oxidizers considered are peroxide and chlorine dioxide. Commonly used non-oxidizing biocides include 2,2-di-bromo-3-nitrioproprionamide (DBNPA) and isothiazolones.

##### Classification

Traditionally, biocides are classified by their mode of action [15,88,89]. General classifications include electrophiles, with the sub-classes of extreme- and moderate-electrophiles, or cell membrane active, with the sub-classes of lytic biocides and protonophores. [88]. Extreme electrophiles are oxidants. This sub-class includes halogenated compounds (e.g., chlorine, bromine, chlorine dioxide) as well as other oxidants, including hydrogen peroxide (typically used with peracetic acid for membrane systems) and ozone. These oxidizers exhibit rapid kill by entering the cell wall and either halogenating intracellular macromolecules (via chlorination or bromination) or generating free radicals within the cell (via chlorine dioxide and hydrogen peroxide) which are toxic to intracellular materials, including carbohydrates, proteins, lipids, and nucleic acids [15]. Note, however, that some oxidizers also react with EPS which creating a barrier layer preventing more of the oxidizer form entering the cell, which is counter-productive [89]. Moderate electrophiles include aldehydes, DBNPA, isothiazolones, and carbamates. The moderate electrophiles also interact with intracellular materials (specifically thiols) but their rate of action is slower, as their entrance into the cell is via diffusion [88].

Cell-membrane active biocides are not as aggressive against microbes as electrophiles; they focus on disrupting the function and physical structure of the cell membrane [81,88]. Lysis of the cell occurs which has two direct affects: leaking of intracellular material and entry of the biocide to interact with phospholipids in the cytoplasmic membrane. End result is that the cell membrane eventually loses integrity [88]. Lytic biocides include select surfactants and quaternary ammonium compounds. Protonophores include parabens (*para*-hydroxybenzoates and esters of *para*-hydroxybenzoic acid) and weak organics acids. Cell lysis using protonophores is not a rapid as with lytic biocides, and protonophores are ineffective against ubiquitous Gram-negative bacteria [81,88]. Hence, they protonophores generally not used for industrial and municipal treatment [88].

##### Efficacy

There are several factors that influence the efficacy of a specific biocide. These include the characteristics of the biocide, the type and condition of the bacterium (e.g., its growth state), the characteristics of the biofilm, and the environment within which the bacterium exists. Also, the type of biocide, its concentration, side reactions that generate inert compounds, and presence of other species, such as organics that compete for the biocide, impact the efficacy of the treatment. Environmental conditions, including pH and temperature of the solution, are important to the efficacy of disinfection. Finally, the time of exposure of the microbe to the biocide is another key to efficacy of treatment.

##### Oxidizing Biocides

An oxidizer’s efficiency for disinfection is measured based on many factors, including reduction potential and available chlorine (for chlorine-based biocides). Table 3 lists the reduction potential for select oxidizing compounds.

For chlorine-based compounds, a higher reduction potential describes only part of a biocide’s disinfection kinetics or efficacy. Available chlorine also plays a role. Table 4 shows the standard electrical potential, available chlorine, and kinetics of 1-log reduction of *Giardia* for several chlorine-based oxidizers. Available chlorine is used to compare the strength of chlorine-based compounds to that of chlorine gas. It simply measures the oxidizing capacity of a compound to oxidize iodide from a potassium iodide solution to iodine, as compared to that of chlorine gas. For example, hypochlorous acid has an available chlorine value of one mole, as one mole of the acid will liberate one mole of iodine. For dichloramine, it liberates two moles of iodine, so its available chlorine is two moles. Available chlorine is often represented as weight percent, where chlorine gas has 100% available chlorine [90].) As Table 4 shows, the kinetics of disinfection are not directly related to reduction potential or available chlorine. It is a combination of properties that determine kinetics; this makes it difficult to select an oxidizing biocide based only on a review of singular properties.

##### Non-Oxidizing Biocides

Due to the degradation of polyamide membranes upon exposure to oxidants, there are few options to for directly disinfecting these membranes [92]. Some non-oxidizing biocides are compatible with polyamide membranes, while others have deleterious effects on performance. Compatible biocides, such as isothiazolones, can be used continuously, intermittently (shock), or for off-line soaking. The need for off-line feed depends on application-specific regulations (e.g., potable and food and beverage applications generally require off-line feed of non-oxidizing biocides) [93]. In many cases, shock-dosing is preferred to avoid adaptation of the populations to the biocide [94,95]. Currently, non-oxidizing biocides are seldom used as the primary mode of biocontrol. They are used to compliment another technique applied in the pretreatment (typically an oxidizer) or to keep membranes free of microorganisms between membrane cleanings via direct application to the membrane.

##### Ideal Biocide for RO Systems

The most prevalent biocide used in membrane systems in chlorine [96]. However, chlorine has some significant limitations. It reacts with organics in water to form THMs and HAAs. Chlorine oxidizes polyamide membranes, so it, and all other oxidizing biocides, must be removed prior to the membranes [97,98]. This leaves the membranes open to biofouling post dechlorination. Finally, chlorination of seawater, which contains high concentration of bromide, yields hypobromous acid, also damaging to polyamide membranes. Given these limitations of chlorine treatment, it is not ideal for use with polyamide membranes for biofouling control.

Bates [21] identified characteristics of an ideal biocide for polyamide membranes (reprinted with permission from the Engineers’ Society of Western Pennsylvania):

- “Does not damage the membrane
- Controls and kills all strains of bacteria and biofilms
- Physically breaks up existing biofilms
- Compatible with all system components
- Nontoxic and easy to handle
- Easily disposed of and biodegradable
- Easily monitored and injected
- Disinfects the permeate side of the membranes
- Inexpensive.”

Clearly, such an ideal biocide does not currently exist; biocides currently available for use with polyamide membranes systems fall short of many, if not all, of these features.

#### 3.1.2. Oxidizing Biocides for Membrane Pretreatment Disinfection

In the following sections, oxidizing biocides are discussed in detail. Chemical forms and generation, dosing, efficacy, application, advantages and limitations, and removal techniques are described. This provides a complete basis for users of industrial and municipal membrane systems to evaluate options. In some cases, the optimal oxidizing biocide is site specific, depending on factors such as local regulations, capital and operating costs, ease of use/training requirements, space requirements, and permeate quality requirements. It should be emphasized that oxidizing chemicals are not compatible with polyamide membranes, as discussed herein Reaction kinetics of various oxidizers with the polyamide varies considerably such that a few biocides that exhibit slower kinetics, such as chloramine, could, under the appropriate feed water conditions, be used in direct contact with the membrane (but in the case of chloramines, is not recommended). Hence, disinfection with oxidizing biocides is limited to addressing the pretreatment system prior to the RO membranes rather than by direct application to the membrane.

##### i. Chlorine

As described previously, chlorine is the most commonly used biocide to disinfect a membrane desalination system. It is an oxidizer, and, as such, destroys microbial, cellular membranes and internal content. Specifically, three mechanisms have been proposed for bacterial inactivation via chlorine [90]. Direct oxidation of the cell wall and the interior components is proposed which leads to leaking of cell components through the oxidized cell wall. Another mechanism is inactivation of key enzymes [99] that are responsible for enabling bacteria to metabolize glucose. Finally, chlorine disables nucleic acids, making them unable to function which leads to death of the microorganism.

The reaction kinetics and efficacy of chlorine disinfection of specific bacterium is a function of several conditions [90]. The nature of the type and concentration of the organism(s) present and environmental conditions contribute to efficacy. Environmental conditions impacting efficacy include concentration of chlorine, contact time, solution temperature and pH, and the presence of other, interfering substances that exhibit a chlorine demand (e.g., organics, transitions metals).

Forms of Chlorine

The two commonly used forms of chlorine include sodium hypochlorite (bleach) and gaseous chlorine. Gaseous chlorine is more toxic than hypochlorite and requires careful handling. It is, however, more economical than hypochlorite, special handling not withstanding [100]. Chlorine gas does not remain in gaseous form in water, which inhibits its ability to penetrate biofilm; it immediately hydrolyzes in water (Equation (1)) to form aqueous hypochlorous acid, HOCl: Liquid sodium hypochlorite also hydrolyzes in water (Equation (2)) to form hypochlorous acid:

Cl_2_ + H_2_O ← → HOCl + HCl (1)

NaOCl + H_2_O → HOCl + NaOH (2)

The formation of hypochlorous acid using chlorine gas yields hydrochloric acid, while sodium hypochlorite forms caustic, thereby reducing or raising the pH of the water, respectively. These effects may have implications as to the scaling potential of the feed water for calcium carbonate (scales at higher pH) and calcium sulfate (scales at lower pH).

Hypochlorite ion, OCl^−^, is formed from the decomposition of hypochlorous acid that is somewhat unstable in solution:

HOCl ← → H^+^ + OCl^−^ (3)

**Figure 6:**
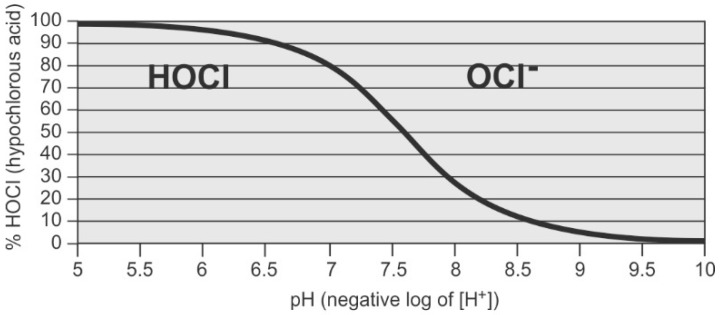
Speciation of chlorine, as hypochlorous acid or hypochlorite ion, in water as a function of pH at 15 °C. Courtesy of Scrivener Publishing [83].

Temperature, salinity, and pH all influence the stability of hypochlorous acid. Figure 6 shows the speciation of hypochlorous acid and hypochlorite ion as a function of pH.

Hypochlorite ion has a lower reduction potential than hypochlorous acid (see Table 3) and is a less powerful disinfectant [90]. Microorganisms are negatively charged, making entry of the negatively charged hypochlorite ion into the microorganism difficult [32,90]. The hydrated ion is much larger in size than the acid and, hence, the resulting kinetics of diffusion into bacterial cells is slower [90]. Also, disinfection reactions to disrupt the functions of enzymes and nucleic acids are favored at lower pH, where hypochlorous acid, and not the ion, predominates [101]. Most industrial desalination systems operate between pH 6.5–9, so operators need to be aware of which form of chlorine is present and the relative efficacy for disinfection.

Determination of Chlorine in Water

The most popular methods for determining chlorine residual include amperometric titration and the *N*,*N*-diethyl-*p*-phenylenediamine (DPD) colorimetric tests [90]. Both are capable to detecting both free (hypochlorite, + hypochlorous acid + chlorine gas + trichloride ion) and total chlorine (free + chloramine (combined chlorine)). Amperometric titration is considered the “gold standard’ to which other methods are compared [90]. However, it is not well suited for field use, although some portable titrators are available. The DPD method colorimetric testing is typically used in the field for quick determination of free and total chlorine. This technique has a lower detection limit (0.01–4 ppm) than the amperometric titration method (0.1–10 ppm) [90]. The main advantage of amperometric titration is that it doesn’t suffer the degree of interference with color and particulate matter that hinder the DPD colorimetric test [90].

Another common method for in-line detection is oxidation-reduction potential (ORP). Specifically, the ORP measures the net potential in an aqueous solution that contains both oxidants, such as chlorine, and reductant, such as bisulfite. The ORP measures the actual activity of the oxidizer or reductant. Free chlorine will register an ORP reading of about 500–700 mV (depending on the pH, chlorine residual and feed water characteristics [90]). The amperometric and DPD colorimetric methods measure just residuals, which are subject to interferences from other compounds in the solution whereas the ORP measure the actual oxidation potential of the feed water. OPR is commonly used to control dechlorination, and the ORP controller can be tuned to yield a slight residual of sulfite, even under changing chlorine/sulfite activity levels [90], thereby ensuring the oxidizer has be removed.

Dosing and Efficacy

Critical factors affecting performance of chlorine are exposure time, pH, and method of application. Hypochlorous acid has a higher potential than hypochlorite ion (see Table 3), and is, therefore, preferred for disinfection. Since pH affects the state of chlorine (hypochlorous acid versus hypochlorite ion, Figure 6), a pH below 7.5 is desirable for disinfection, as a greater proportion of chlorine is present as hypochlorous acid. Disinfection for the pretreatment system requires an exposure time of 20–30 min with a residual of 0.5–1.0 ppm at the point of dechlorination [34,50]. The ASTM method D 1291, “Standard Practice for Determining Chlorine Requirement of Water” is useful to determine the optimal dosage necessary to achieve the required residual.

There are three primary methods of applying chlorine: continuous, shock, and continuous plus shock. Shock treatment can be effective, but continuous feed, which is the most commonly practiced method, has greater efficacy than shock treatment only for disinfection [34]. For a specific seawater system with a continuous residual of 0.04–0.05 ppm free chlorine in the pretreatment, biofilm was not observed on the RO membranes [87]. Shock treatment alone is thought to minimize the potential for bacteria to become conditioned to continuous feed. Continuous feed plus shock treatments can help to overcome the conditioning of bacteria to chlorine. This method was shown to be more effective at controlling membrane biofouling than either singular method during warm weather, when bacterial activity is enhanced [96].

Advantages and Limitations

Chlorine offers several advantages over other oxidizing biocides for disinfection of membrane desalination systems [102]. Chlorination is a well-established, cost-effective disinfection technique. It allows for flexible dosing control and carries a residual in water. Residuals are important for continued biocontrol downstream of the biocide injection point.

There are some distinct limitations to using this chlorine for disinfection. On a universal level, chlorination forms THMs and HAAs, both of which are carcinogenic species. Hypochlorous acid can also form disinfection by-products (DBPs), such as bromate (in seawater—maximum contaminant level, MCL, of 0.01 ppm) and chlorate (no official MCL, but California has a notification level of 0.8 ppm) [100,103]. *Cryptosporidium parvum* and *Mycobacterium avium,* which are pervasive in water system biofilms, are not well controlled using chlorine; endospores and protozoa are also not well controlled with chlorine [104,105].

Chlorine also oxidizes organics, such as longer-chained humic acids, cleaving them into shorter-chained molecules. These smaller molecules are transformed into AOC that microbes can more easily digest. This is a particularly serious problem after the point of dechlorination [50,87,89,106]. Studies have shown increased rates of biofouling following dechlorination [106,107,108]. The absence of disinfectant, the degree of disinfection as opposed to sterilization of the system, and the availability of more AOC post chlorination, lead to greater rates of biofouling post dechlorination.

In seawater systems, chlorination leads to the formation of free bromine (typically present as hypobromite ion and hypobromous acid, both of which are pH dependent similar to hypochlorite ion and hypochlorous acid), due to the high concentration of bromide (about 65 ppm) naturally occurring in seawater [109]. Research by Shemer and Semiat [109] suggested that free bromine was more stable and more aggressive to polyamide membranes than free chlorine. As a result, some seawater membrane plants have stopped the use of chlorination all together, with variable results [110,111,112].

**Figure 7:**
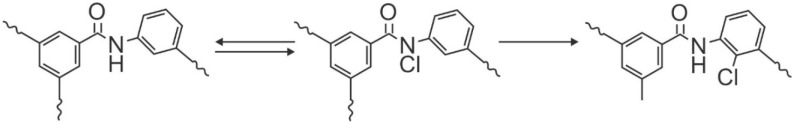
Chlorine damage to polyamide membrane via hydrogen-bond destruction and subsequent ring substitution via Orton Rearrangement

Perhaps the biggest limitation to the use of chlorine is that it oxidizes membranes, destroying the integrity of the polyamide layer. Chlorine substitutes onto the amide functional group followed by destroying the hydrogen-bond linkages in the polymer that ultimately results in ring substitution by the halogen via Orton Rearrangement, as shown in Figure 7 [97,98,113]. The degraded membrane passes more water and loses its ability to reject solutes; these results are irreversible.

Dechlorination

Due to the damage inflicted on polyamide membranes, free chlorine must be removed prior to the membrane system. In general, ORP is used to determine when free chlorine is no longer present; a reading of less than 200 mV is generally assumed to indicate the absence of free chlorine [114]. Chlorine analyzers are sometimes used but they are designed to measure the presence of chlorine rather than the absence of it. If an analyzer is used, free chlorine residual should be measured at less than 0.02 ppm to minimize degradation of the membranes [83]. Dechlorination is relatively straight- forward. Most system rely on sodium metabisulfite to chemically remove free chlorine. Carbon filtration (which relies on an oxidization/reduction reaction to reduce chlorine) is also used frequently. UV radiation is used less commonly for dechlorination.

Sodium Metabisulfite

Dechlorination involving sodium metabisulfite follows a 2-step process, where the sodium metabisulfite first forms sodium bisulfite in water:

Na_2_S_2_O_5_ + H_2_O → 2NaHSO_3_ (4)

Hypochlorous acid is then reduced by sodium bisulfite:

2NaHSO_3_ + 2HOCl → H_2_SO_4_ + 2HCl + Na_2_SO_4_ (5)

Sodium metabisulfite can be purchased as a dry product and then dissolved in water to form sodium bisulfite. Alternatively, sodium bisulfite, as a liquid, can be used directly for dechlorination. Many systems employ the liquid sodium bisulfite for ease of handling. Non-cobalt catalyzed product is required, as the cobalt in the presence of free chlorine that was not scavenged, will catalyze chlorine destruction of the membrane [21]. In theory, every ppm of free chlorine requires 1.47 ppm of sodium bisulfite or 3.0 ppm of sodium metabisulfite for reduction [83]. Since most sodium bisulfite solutions are about 33–37% active, the theoretical dosage would be about 3.5–4.5 ppm per ppm of free chlorine. Often, a safety factor of 1.5 to 2 times theoretical is applied to determine the actual dosage [83].

ORP is generally used to confirm removal of free chlorine. When using bisulfite, an ORP value of less than 175–200 mV is recommended to ensure protection of the membranes from chlorine attack [115]. Care should be used to not grossly overfeed bisulfite to the point of a negative ORP, as this produces a reducing environment friendly to proliferation of anaerobic bacteria [116,117].

Activated Carbon Filters

Free chlorine can be removed from aqueous solution via an oxidation/reduction process using activated carbon. Chlorine is reduced to chloride while the carbon is oxidized, per the following reactions:

Cl_2_ + H_2_O + Carbon → 2 HCl + Carbon-O* (6)

HOCl + Carbon → HCl + Carbon-O*(7)

OCl^−^ + Carbon → Cl^−^ + Carbon-O* (8)

where Carbon-O* is surfaced-oxidized carbon. These reactions are instantaneous, so empty bed contact time (EBCT) can be a low as 3 min for free chlorine removal, however, allowances for up to 5–10 min of EBCT is typical [83,118]. Service flow rate for dechlorination prior to a membrane system is 2 gpm/ft^3^, with a minimum bed depth of 2.5 ft. General service life for carbon that is used for dechlorination is about 12–18 months.

Carbon filters are known for incubating microbes [78,79,119,120]. Microbes can slough off with carbon fines or on their own out of the filter and travel downstream to infect the membranes. To eliminate this issue, it is recommended to use sodium bisulfite for dechlorination [21], unless other circumstances prohibit the use of bisulfite: the feed flow rate is less than about 30 gpm, where control of the bisulfite chemical feed would be difficult; organic (TOC) removal is also required; or there is a process concern with using bisulfite (e.g., in food and beverage applications).

Ultraviolet Radiation (UV)

An alternative method to chemical addition or carbon reduction for dechlorination is UV radiation. High-intensity, broad-spectrum UV (medium-pressure UV), can reduce chlorine gas at peak wavelengths of 180–200 nm, while 292 nm is ideal for hypochlorous acid destruction. A UV dose of 45–90 mJ/cm^2^ or about 150% to 300% of that required for general disinfection can dissociate as much as 15 ppm of free chlorine into chloride ions [121,122]. (Specific dosage depends on several factors, including organic concentration, total chlorine present, and the ratio of free chlorine to chloramines [121]). The UV technique offers that advantages of chemical-free dechlorination, and elimination of the problem of sloughing off bacteria that occurs when using a carbon filter.

##### ii. Combined Chlorine (Chloramine)

Combined chlorine, which includes the oxidizers monochloramine, NH_2_Cl; dichloramine, NHCl_2_; and nitrogen trichloride (trichloramine), NCl_3_, is also used to disinfect membrane-based desalination systems. Monochloramine has a reduction potential similar to hypochlorous acid (as shown in Table 3) but has lower biocidal power (0.4% of hypochlorous acid) [123] and slower reaction kinetics (see Table 4) [124]. Studies have shown that chloramines require up to 100 times the contact time or 25 times the concentration of free chlorine to inactivate some bacteria, such as *Escherichia* *coli* (*E. coli)* [125,126,127]. It can take days or weeks to achieve disinfection [105]. The slower reaction kinetics of monochloramine leads to lingering residuals in distributions systems (as opposed to chlorine, which dissipates more rapidly). Thus, municipal distribution systems benefit from the lingering monochloramine residual, making it more effective than chlorine in the long term. While water distribution systems benefit from the slower reaction kinetics, membrane pretreatment requires more rapid kinetics. Many municipalities are shifting from using strictly chlorine to chloramine [128] due to ever stricter regulations on DBPs resulting from chlorination. Operators of membrane systems with chloramine-treated municipal make-up water need to be cognizant of this disinfectant, and its effects on the membrane system.

The mechanism for chloramine attack on microorganisms is not well known but may involve inhibition of proteins or functions such as respiration (a protein-facilitated processes) [129]. Jancangelo et al. [129], found that the inactivation process is inconsistent and, therefore, monochloramine should be feed at several discrete intervals to ensure that disinfection occurs.

The comparison of chloramines to free chlorine is not straight forward, as disinfection performance depends on pH and other factors (including temperature and exposure time) [90]. Monochloramine is more effective at higher pH than the hypochlorite ion, whereas hypochlorous acid is more effective than monochloramine at lower pH. [101]. In most membrane desalination systems, the feed water pH runs about 6.5–9, so it follows that at the lower end of this range, hypochlorous acid would be more effective, but at the higher end of the range, monochloramine would be more effective.

Chloramine Generation

The combining of free chlorine (as hypochlorous acid) with free ammonia (NH_3(g)_) is known as chloramination [130]:

HOCl + NH_3_ ← → NH_2_Cl + H_2_O (9)

2HOCl + NH_3_ ← → NHCl_2_ + 2H_2_O (10)

3HOCl + NH_3_ ← → NCl_3_ + 3H_2_O (11)

**Figure 8:**
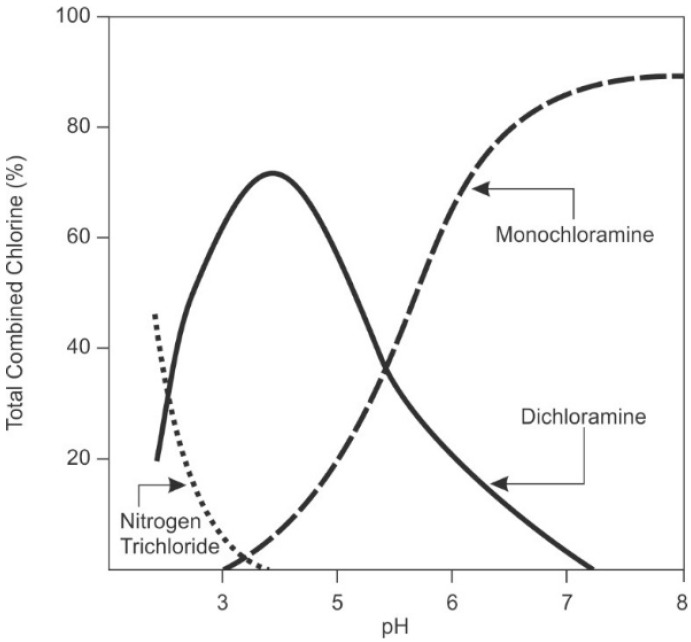
Combined chlorine as a function of pH. Nitrogen trichloride (trichloramine) is only present at pH less than about 3.5; dichloramine predominates at pH ranging from about 2–5.5; and monochloramine is the predominant species at pH greater than about 5.5.

These reactions are strongly dependent on pH, as shown in Figure 8, relative concentration of the reactants, and temperature [131]. The reaction kinetics of formation are minimized at pH 8.4 and 25 °C [90]. Stoichiometrically, Equation (9) shows that one mole of monochloramine forms in the reaction of one mole of hypochlorous acid with one mole of ammonia. This corresponds to a chlorine, (as ppm Cl_2(g)_) to ammonia (as ppm NH_3_-N), ratio of about 4.5 to 5.0. Higher ratios will slow the reaction [90], lead to breakpoint chlorination, and generate dichloramines, which are notorious for taste and odor issues [90]. Lower ratios increase the potential for nitrification to occur [90]. Lower pH favors formation of di-and trichloramine [90], although little trichloramine persists under typical water treatment conditions. Lower temperature will slow the reaction kinetics.

Dichloramine has been shown to be twice as strong of a biocide as monochloramine [101] but is objectional due to its taste and odor issues [90]. While being an even stronger oxidizer than dichloramine, little information regarding the biocidal effects of trichloramine are known, as it has highly objectionable taste and odor, and so its formation is avoided in water treatment [90].

Determination of Chloramine in Water

The most common method of determining chloramine in water is by subtraction of free chlorine (determined by DPD free chlorine method) from total chlorine (determined by the DPD total chlorine method) [132]. However, many organic chloramines also produce color for the DPD total method [70], which introduce error into the measurement of chloramine available for disinfection [132,133,134]. Despite this, the DPD subtraction method is the most commonly used field test for chloramine.

A modified indophenol method using MonochlorF (Hach Company, Loveland, CO, USA) reagent does not have interference with organic chloramines or transition metals [132], and measures only monochloramine, as opposed to all species of chloramine [135]. Research by Lee et al. [132], concluded that determination of the actual concentration of monochloramine is a better indicator of the actual disinfection residual than the DPD subtraction method [132,136].

Dosing and Efficacy

Ideally, chlorine is added to ammonia to generate chloramine, as this method reduces or eliminates chlorinous taste and odor [90]. Further, this technique minimizes the potential for damage to the polyamide membrane from free chlorine or the chloramine itself [137]. For wastewater applications, where ammonia is naturally present, addition of chlorine directly to the wastewater to form chloramines has worked successfully at Water Factory 21 (predecessor to the Ground Water Replenishment System, GWRS, (Orange County, CA, USA)) to minimize biofouling [138,139]. This was accomplished *without* the need for dechlorination/dechloramination or damage to the polyamide membranes [96]. The hypothesis for this result is that the high ammonia concentration and organic content of secondary effluent protects the polyamide membrane from Orton Rearrangement and subsequent membrane damage that occurs with chlorination of membrane [137]. The exact mechanism of interaction between chloramines and polyamide membranes is not yet understood, however.

**Figure 9:**
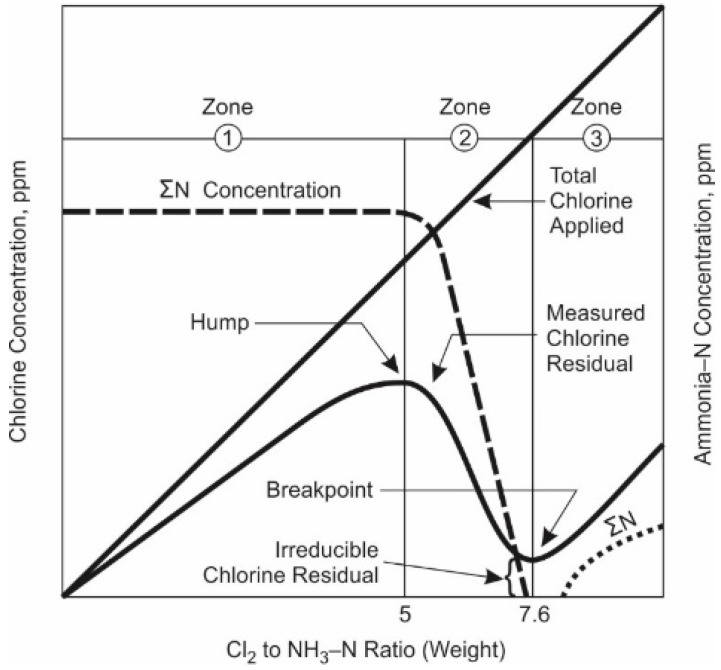
Breakpoint chlorination.

In industrial and municipal practice, for pretreatment of surface and ground water, chlorine is first added to the water to be treated (known as primary disinfection), and then ammonia is added to form chloramines (secondary disinfection) [90]. The rationale for adding ammonia to chlorinated water is that initial chlorination will result in more rapid disinfection and the resultant chloramine after ammonia addition will then linger throughout the distribution system [90]. Good mixing is essential to avoid side reactions with organics that yield organic chloramines, which have no biocidal effect. The full understanding of chloramines to treat water requires the understanding of breakpoint chlorination. Figure 9 illustrates breakpoint chlorination.

Chloramination occurs in Zone 1 of the figure where chlorine is combined with ammonia to form monochloramine. Note that there may be a lag in the initial curve due to species in the water that put an immediate demand on chlorine, such as iron and manganese. Once the immediate demand has been satisfied, the maximum concentration of monochloramine occurs when the ratio of Cl_2_ (gas) to NH_3_-N is 5.0. This is the “monochloramine hump.” For systems using monochloramine, the desired operating range is embodied in Zone 1. Addition of chlorine post Zone 1 results in the formation of dichloramine via chlorine reaction with monochloramine and ammonia (Zone 2). A series of dichloramine reactions then follow and continue until “breakpoint” is achieved, where the following equation approximates the situation [90]:

3Cl_2_ + 2NH_4_^+^ → N_2_ + 6Cl^−^ + 8H^+^ (12)

At breakpoint, the composition of the solution includes primarily dichloramine, with trace amounts of free chlorine, monochloramine, and organic chloramines; total concentrations may be a few tenths of a ppm [90]. After breakpoint, in Zone 3, the free chlorine residual increases proportionally to the chlorine dose.

Efficacy for control of membrane biofouling with chloramine is questionable [26]. While some report good biofouling control for wastewater reuse systems [96], Raffin et al. [140], found little to no benefit to direct contact of chloramine with polyamide membranes for this application. The theory promoted by Raffin et al. [140] is that the low rejection of chloramine by the polyamide membrane leads to not enough chloramine residual in the concentrate stream for adequate disinfection [140]. A literature search found little discussion of efficacy for control of ground or surface water; most research was focused on compatibility with the polyamide membrane. In general, chloramines are not as effective for disinfection compared to hypochlorous acid [26]. The assumption is, then, that they would be less effective at membrane biofouling control than hypochlorous acid.

Advantages and Limitations

The primary advantage of chloramination is its moderated formation of DBPs relative to chlorination, since they chloramines not as reactive as chlorine is with organics [136]. Chloramine has proven successful at controlling biofouling of membranes for wastewater reuse applications [141], where chlorine is added to ammonia naturally present in the feed source. However, other than membrane systems operating on chloraminated, municipal feed water, the use of chloramines has not been widespread to directly treat surface and ground water [141]. Lozier [137] studied compatibility of polyamide membranes with chloramine operating on ground water with low concentration of organics, and high-organic loaded surface water. In both cases, the membranes suffered increases in specific flux and loss of salt rejection as compared to control membranes not exposed to chloramine. Lozier [137] concludes that until further study is undertaken to understand the apparent synergistic effects of the specific water matrix and chloramine on degradation of the polyamide membrane, that chloramines may be best suited to wastewater reuse applications only.

Some report that most polyamide membranes can tolerate longer exposures to *pure* chloramine, anywhere from 150,000 to 300,000 ppm-hours [21,142,143]. However, pure chloramine is elusive due to the equilibrium of chloramines with free chlorine and ammonia (Equations (9)–(11)). Research by Lozier [137], Silva et al. [144] and Maugin et al. [145] indicates the that chloramine itself attacks polyamide membranes. Maugin et al. [145] found that just 20,000 ppm-hours of exposure resulted in a 70% increase in permeability and a linear decrease in salt rejection. The effects of temperature and pH on the equilibrium equations, and any light metals, such as aluminum, iron, and manganese, that may be present can significantly shorten membrane life when exposed to chloramines [21,143,144].

One proposed mechanism for membrane degradation via chloramines is similar to that for chlorine degradation of polyamide membranes [146]; amidogen radicals (NH_2_) are formed (catalyzed by the presence of metals ions) which attack the ring structure and allow for direct chlorination by excess chloramine [146]. The difference versus free chlorine is that the kinetics of membrane degradation via chloramine are slower and chloramine is less aggressive [147]. Maugin et al. [145], on the other hand, found evidence, using Rutherford backscattering spectrometry (RBS) and Fourier transform infrared spectroscopy (FTIR) analyses of virgin, chlorinated, and chloraminated polyamide membranes, that different reaction mechanisms may be involved for degradation via chlorine and chloramine exposure.

The potential for free chlorine to be in equilibrium with monochloramine is great (see Equation (9)). This coupled with the uncertainty regarding polyamide membrane compatibility with chloramine, makes dechlorination a necessity prior to polyamide membranes [143]. Dechlorination can lead to dechloramination, which can increase the ammonia gas or ammonium ion concentrations, depending on the pH [21].

Free ammonia (gas) can also be in equilibrium with chloramine (Equation (9)) and will affect the membrane system in negative ways. Nitrification of the system can occur since free ammonia is a nutrient source for nitrifying bacteria [130,136]. Gases, including ammonia, are not rejected by an RO membrane. Hence, the concentration of ammonia gas in the feed water will be the same as that in the permeate, which can go on to corrode non-stainless steel metal components and piping downstream. Ammonia gas swells polyamide membranes, causing a *reversible* increase in ion passage through the membrane. Swelling occurs at a pH of greater than about 7.5. If the pH is lowered to below 7.5, the ammonia (gas) will be converted primarily to ammonium ion, which does not swell the membrane and is also well rejected by an RO membrane [83]. Since most membrane desalination systems operate at pH 6.5–9, the presence of free ammonia can be a concern.

Process variables in forming chloramines are difficult to control in actual practice, which can shift Equations (9)–(11). Additionally, if the TOC concentration exceeds about 3 ppm, organic chloramines can be formed in addition to the products shown in Equations (9)–(11). Organic chloramines have little or no disinfecting capabilities. Due to these factors, field testing has been inconclusive as to the efficacy of chloramines in controlling biofouling of polyamide membranes [87].

Chloramination of seawater systems is not recommended [96]. The exposure of bromides in seawater to chloramine (ammonia) forms bromamines. Bromamine has several times the oxidation strength of chloramine [96,148], and has as much biocidal activity as hypobromous acid [148]. Severe membrane damage was incurred at West Basin Municipal Water District (Carson, CA, USA), during a pilot study on seawater intake due to the formation of bromamines via chloramination [100].

Dechloramination

Chloramines are considered a mild oxidizing agent [143] and, due to the inconclusive data regarding polyamide membrane compatibility, chloraminated water should be removed prior to the membranes There are several methods to remove chloramine, including sodium thiosulfate, ascorbic acid, sodium metabisulfite chemical addition, carbon filtration, and, occasionally, UV radiation., with the later three being the most common methods [143].

Sodium Metabisulfite

Dechloramination with sodium metabisulfite follows a 2-step process, similar to dechlorination with sodium metabisulfite. The first reaction, Equation (4), shows the reaction of sodium metabisulfite with water to form sodium bisulfite, which then reacts with monochloramine as follows:

NH_2_Cl + NaHSO_3_ + H_2_O → NaHSO_4_ + NH_4_Cl (13)

The reaction for sodium bisulfite is rapid (albeit slower than the reaction with chlorine), requiring from 1 to 5 min of contact time [149,150], with complete mixing essential.

Carbon Filtration

Chloramine is effectively removed from aqueous solution using activated carbon in an oxidation/reduction process. As with dechlorination, the reaction with chloramines is an oxidation reaction process. However, the process for complete chloramine removal, including free chlorine and ammonia, is considerably more complicated. Work by Champlin et al. [151] and Bauer et al. [152], demonstrated that removal of chloramine at Cl_2_:NH_3_-N ratios of less than 7.6 (breakpoint) is governed by the following equation:

NH_2_Cl + H_2_O + Carbon → NH_3_ + Carbon-O* + H^+^ + Cl^−^ (14)

This reaction was found to be instantaneous. Note that this process leaves behind free ammonia. As discussed previously, free ammonia, at a pH of greater than about 7.5, will swell the polyamide membranes, resulting in a reversible increase in salt passage. For complete removal of the ammonia, Champlin et al. [151] and Bauer et al. [152] determined that additional chlorine must be added to achieve breakpoint. At breakpoint, the reaction is shown in Equation (15) shows complete removal of chloramine (and ammonia) to nitrogen gas [151]:

2NH_2_Cl + HOCl → N_2(g)_ + 3H^+^ + 3Cl^−^ + H_2_O (15)

Kim and Snoeyink [153] described an additional reaction, which results in direct removal of chloramine without the intermediate formation of free ammonia:

2NH_2_Cl + Carbon-O* → N_2(g)_ + 2H^+^ + 2Cl^−^ + Carbon-O* (16)

They determined that after an initial acclimation period about 50% of the monochloramine reacted according to Equation (14) and the remainder reacted according to Equation (16) (Bauer et al. [152] determined this acclimation period to be about 20 h).

Contact time for removal of chloramine using activated carbon is critical [153]. Kim and Snoeyink [153] determined that longer contact time, with flows of less than 1 gpm/ft^3^, resulted in the most efficient removal of monochloramine. Potwora [154] indicates that a flow rate of 0.75 gpm/ft^3^ is required for removal using standard activated carbon. Further, Kim and Snoeyink [153] found that the residual monochloramine concentration was about 0.1 ppm after carbon filtration, and that longer contact times did not change this final, effluent concentration. The age of carbon also impacts contact time; new carbon can require as little ss 5 min of empty bed contact time, while older carbon can require up to 30 min.

Ultraviolet Radiation

As with UV destruction of chlorine, UV can also be used for chloramine destruction. The dose required for chloramine photolysis is about 200% that typically used for disinfection, or about 60 mJ/cm^2^ [122]. Wavelengths of 245–365 can remove up to 5 ppm of chloramine in a single pass [121]. Wavelengths for optimum photolysis of various forms of chloramine are 245 nm for monochloramine, 297 nm for dichloramine and either 260 nm or 340 nm for trichloramine.

##### iii. Ozone

Ozone (O_3_) or trioxygen is a very strong oxidizing biocide. The use of ozone itself yields minimal DBPs in most applications, except by-products formed by reaction with bromide present in seawater. Seawater by-products include bromate [155,156,157], a carcinogen [158] that is not well rejected by polyamide membranes [26], and bromo-organic compounds, such as bromoform [155].

Ozone was experimentally used to disinfect of water in 1886 [159], and first employed commercially for this purpose in 1906 operating on the Vesubie River at the Bon-Voyage facility in Nice, France [160,161]. Today, thousands of water treatment plants utilize ozone as part of their chemical water treatment [160], with 72% of ozone users in the United States reporting employing ozone for disinfection [160].

Ozone is very unstable in water, with the kinetics and reaction products of decomposition depend on the pH and alkalinity of the water, and type and concentration of natural organic matter (NOM) present [162]. Decomposition of ozone in water forms several free radicals, with hydroxyl radical, OH* being primary [162]. The free radicals formed are in themselves very strong oxidizers capable of disinfection and react within a few microseconds [160].

Primary action of ozone with microorganisms follows one of three pathways [162]: direct reaction with ozone itself, indirect reaction with radicals that form when ozone decomposes in water, or both. For indirect reaction with radicals, ozone in water initially reacts with hydroxide ions to form superoxide anion (O_2_−) and hydroperoxyl radical (OH_2_*):

O_3_ + OH^−^ → O_2_ + HO_2_* (17)

In following reactions, ozone and superoxide anion react to form ozonide radical (O_3_*), which further reacts to form several radicals, including HO_2_*, HO_3_*, HO_4_*, and the afore mentioned OH* [160]. Finally, termination reactions occur, during which formation of free radicals is inhibited; these involve the reaction of hydroxyl radical with carbonate or bicarbonate, or the reaction of 2 radicals with each other [160].

Due to the high oxidation potential of ozone and its radicals, these compounds can oxidize the bacterial cell wall. Once inside the cell, ozone and its radicals oxidize all essential cell components, including enzymes, proteins, deoxyribonucleic acid (DNA), and ribonucleic acid (RNA). The cell lyses when the microbial components and its membrane are damaged in such a manner. Shear forces can then be used to break up the organisms and their biomass [77]. Ozone is an effective biocide against bacteria, viruses, protozoa (typically resistant to most disinfectants [155]), and endospores [163].

Ozone Generation

**Figure 10:**
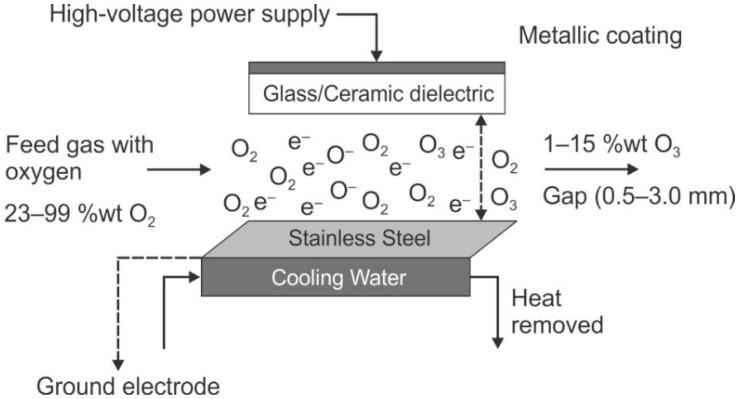
Cross section schematic of an ozone generator. Reprinted with permission of John Wiley & Sons [160].

The instability and rapid decomposition of ozone implies that it must be generated on. The most common method of ozone generation is electrical discharge (also known as coronal discharge) [160,161,164]. Electrical generation involves the exposure of air or pure oxygen to a uniform high voltage/high density alternating current, which drives the reactions. A gap between two electrodes, one of which is coated with a dielectric material, allows an electric field to develop, allowing the reactions to proceed (see Figure 10). Other generation methods include acid electrolysis, UV photochemical reaction, and radiochemically; there are a few other chemical methods that are infrequently used [160,161,164].

The overall chemical reaction for the formation of ozone is shown in Equation (18):

3O_2_ ←→2O_3_ + 0.82 kW/kg (18)

In its simplified form, the initiation reaction (Equation (18)) occurs when free electrons dissociate oxygen molecules into atomic oxygen molecules (O), which then react with additional oxygen molecules in a “three body collision” with any other molecules in the gas, M, to form ozone (Equation (20)) [165,166]:

e^−^ + O_2_ → 2O + e^−^ (19)

O + O_2_ + M → O_3_ + M (20)

The reactions of ozone with atomic oxygen and free electrons (Equations (21) and (22), respectively) are simultaneously occurring to reform oxygen molecules [165]:

O + O_3_ → 2O_2_ (21)

e^−^ + O_3_ → O_2_ + O + e^−^ (22)

Further, when air, rather than oxygen, is used as the feed gas, nitrogen species, such as N^+^, N_2_^+^, and N, complicate the reactions system [166]. Nitrogen oxide radicals are formed that consume ozone, as shown in Equation (23) [166]:

NO_2_* + O_3_ → NO_3_* + O_2_ (23)

A typical ozone generator yields about 0.5–16 wt% ozone to the carrier gas [160]. Only 5–10% of the applied energy is used to generate ozone; the remainder dissipates in the form of heat. Decay reactions in Equations (20) and (21) are favored at higher temperatures, so ozone generators include methods to dissipate heat to discourage these ozone decay reactions.

Once generated, there are several transfer methods to available to contact water with ozone. Fine bubble diffusers are most widely used and are the most energy-efficient means of transfer [153]. Side-stream injectors are commonly used in new installations [153], which offer transfer efficiencies of greater than 95% that does not decline with time. Smaller systems requiring less than 4 km^3^/day of water to be treated typically use packed columns where ozone is bubbled up through a column of water packed with ceramic media. The primary advantage of this technique is that there are no moving parts. U-tube reactors, aspiring turbine mixers, and spray chambers (typically used for oxidation of inorganic compounds rather than disinfection [160]) are also used.

**Figure 11:**
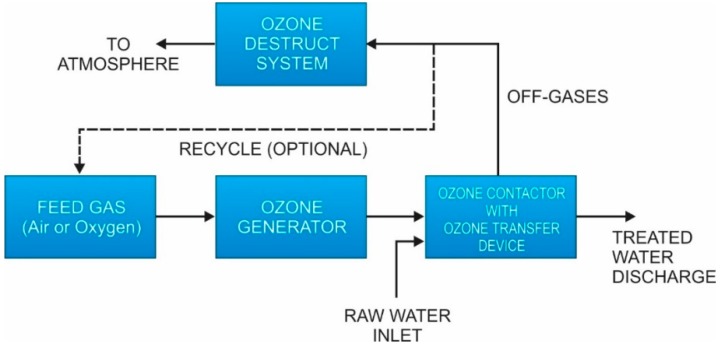
Simplified, conceptual schematic of an ozone generation system. Reprinted with permission of John Wiley & Sons [160].

Considerations in selection of transfer technique include the weight percent of ozone in the carrier gas, ozone gas to water ratio, ozone demand, and the nature of the contactor used for to provide residence time for ozone to react with the raw water [160]. Conventional baffled basins and pipeline contactors are the most common type contactors used for ozone transfer and oxidant contact time [160]. A generalized ozone process flow diagram is shown in Figure 11.

Determination of Ozone in Water

Determination of ozone residual in water is problematic [166,167,168]. The volatility in solution and the continuous self-decomposition reactions hamper efforts to determine residual. Further, the reaction of various ozone decomposition products (radicals) and reaction of ozone itself with organic and inorganic contaminants make residual quantification a challenge. The most direct method for measuring ozone residual involves measuring adsorption at 260 nm (adsorption at 258 nm also shows a maximum for ozone, and the values at the two wavelengths are virtually identical [166]). This method assumes that the water tested has little or no dissolved organic matter (DOM), turbidity, and iron that also adsorb at these wavelengths.

Colorimetric methods, such as DPD and indigo, can be used to field test for ozone residual. The DPD method, however, suffers from interference due to manganese (II) and particulate matter, as described earlier for chlorine testing [166]. Ozonation forms manganese dioxide colloids that readily oxidize DPD, yielding higher than actual ozone concentration values. And, hypobromous acid formed during ozonation of bromide-containing water also reacts with DPD [166]. Interferences make the DPD field test for measuring ozone residual unreliable, unless significant pretreatment is employed to eliminate interfering species.

The indigo thiosulfate test is recognized by *Standard Methods* [169] for determination of ozone residual in water. Although manganese (IV) and (VII) can interfere with the indigo method [167], the interferences are not as troublesome as for the DPD method [170]. Corrections to the procedures can be made to account for interferences by manganese, chlorine, and bromide [160].

Iodometric methods were the reference method used for ozone residual detection, but more recent studies have shown that this method measures all oxidative species present, and, therefore, overstates the amount of ozone present [167,168,171]. This test is considered unreliable, and the recommendation is to use this method (and its variants) only for production testing where other species are not usually present [167].

Amperometric electrodes are used for on-line measurement of ozone residual [166,167]. Bare electrodes that were initially used, suffered from interferences due to other oxidants, including bromine and iodine [167]. The use of gas-permeable membranes, such as Teflon and, more recently, polymeric membranes, have increased selectivity and reduced electrode fouling [167]. These membrane electrodes exhibit less than 2% interference due to bromine, hypobromous acid, hypochlorous acid, nitrogen trichloride (trichloramine), and chlorine dioxide [172,173], and are, therefore, more reliable than other test methods.

Dosing and Efficacy

Dosing and efficacy of ozone as a biocide can be very complex. The keys to successful ozonation are dose, mixing, and contact time [174]. The design of the transfer mechanism (e.g., fine-bubble diffuser) and the contactor are critical to achieving the proper dosages, mixing, and contact time [160]. The multifaceted nature of the target water, including the type and quantity of natural organic matter (NOM) present, temperature, alkalinity, and pH all contribute to the complexity [175]. Suspended solids also impact efficacy, as effectiveness of ozone disinfection directly depends on physical contact of the ozone with the target microbes. Prefiltration is required to remove suspended solids that may obscure microbes from contacting ozone molecules [176]. Ozone dosage is based the amount of ozone needed to inactivate microorganisms and that required to achieve the required disinfection contact time. Since the exact dosage may be difficult to determine due to variability of the target water source, the general practice is to dose enough ozone to achieve a scarcely measurable residual post disinfection [160].

The degree of disinfection achieved, known as disinfection credits, is defined as percent log removal, such as 99% removal is equivalent to 2-log removal) [160]. CT values are used to determine the concentration or contact time required for a specific disinfection credit. CT is calculated as oxidant residual multiplied by contact time, and are reported in mg-min/l. Contact time (in minutes) is defined as the period in which 10% of the feed water through a reactor has passed through, thus ensuring that 90% of the water has been exposed to the disinfectant [160]. Because ozone decays much more rapidly than chlorine-based oxidants, the CT values for ozone tend to be less precise that those for other disinfectants [177]. For ozone, the CT is measured at multiple locations within the contactor and the sum of the CT at various locations is used to determine the disinfection credit [160]. CT values can also be used to compare the efficacy of different types of disinfectants. Table 5, Table 6 and Table 7 show the disinfection credits along with CT values for ozone disinfection of *Giardia* cysts, viruses, and *Cryptosporidium*, respectively. As shown in the tables, ozone is more effective at higher temperatures. In general, every 10 °C increase in temperature hastens the kinetics of ozone disinfection by a factor of 2–3 [177].

Table 8 compares the CT values for ozone as compared to chlorine-based disinfectants for 2-log (99%) inactivation of the indicated microorganism at 5 °C. As the table indicates, ozone is a more effective disinfectant for these organisms than chlorine-based compounds commonly used in water treatment.

Advantages and Limitations

There are a few of advantages of ozone over other disinfection methods. Ozone has greater disinfection efficacy than chlorine [174], and relatively short contact times are required for inactivation of bacteria. Reactions of ozone and its radicals themselves do not persist as hazardous DBP, but do react with other species, primarily bromide and humic compounds, to yield some undesirable compounds.

The primary limitation is the sensitivity of polyamide membranes to oxidation by ozone. While it is well understood that ozone will destroy these membranes upon contact, there is relatively little literature detailing the interaction. Work by Glater et al. [180] in 1983, is a definitive work on oxidation, including ozonation, of RO membranes, while Maugin [145] in 2013, describes mechanisms for interaction and performance decay. Maugin [145] and Glater, et al. [180] found that ozone is more aggressive to the polyamide membrane than chlorine compounds. Tests with polyamide membranes demonstrated severe damage with exposure to 0.3 ppm ozone within 90 h (27 ppm-hours of exposure) [180]. Maugin [145] showed that loss of rejection and increase in permeability of polyamide membranes with ozone at a CT value of 0.015–0.02 millimoles-hour (mM-h) (61.2–81.6 mg-min/L or 1–1.4 ppm-hours) was equivalent to that of a chlorinated (as Cl_2_) polyamide membrane at a CT value of 15–20 mM-h (63,000–84,000 mg-min/L or 1040–1400 ppm-hours (the typical value used to estimate membrane life when exposed to chlorine). Hence, the reaction of ozone with the polyamide membrane is 1000 times faster than for chlorine. Ozone destruction of polyamide membranes involves direct attack of the amide bounds in the polymer leading to depolymerization and ultimate destruction/removal of the polyamide layer [145] (as opposed to the Orton Rearrangement mechanism for chlorine). As a result of ozone destruction of polyamide membranes, ozone removal prior to membrane systems is required [50,76].

Ozone generation inherently produces excess ozone (off gas), ozone that is not transferred to solution, and remains in the gases phase. This hazardous, corrosive gas must also be destroyed at the generator before discharge to the environment [160,164]. Several options are available for destroying the off-gas (known as ozone destruct systems), but thermal-catalytic destruction with a manganese dioxide catalyst is the most common technique [160]. Other techniques include thermal destruction without catalytic destruction and catalytic destruction with metal catalysts [164]. Sometimes the offgas is recycled to the head of the system (as shown in Figure 11), but the benefits of this can be negligible due to the low concentration of ozone and the cost of the recycle equipment [160]. Plus, recycling may reduce but not eliminate the need for destruction of the offgas [160].

As mentioned previously, ozone and its radicals themselves don’t persist as hazardous DBPs, but reactions with other compounds, namely bromide, do yield problematic species. The reaction of ozone with bromide forms hypobromous acid [155], which then further reacts with NOM [181] to yield many bromoorganic compounds, including bromoform, bromoactetone, bromopicrin, dibromoacetonitrile, bromoacetic acid, bromoalkanes, and others [182,183,184]. Fortunately, the concentrations of these compounds are generally much lower than current drinking water standards [155].

One by-product of major concern is bromate, BrO_3_^−^. Bromate is a genotoxic carcinogen that forms via a complicated reaction of ozone and its radicals with bromide [155]; bromate formation steps include up to six oxidation states of bromine. The World Health Organization (WHO) established a guideline for bromate of 0.025 ppm in drinking water [185]. The USEPA and European Union have established a maximum contaminant level of 0.010 ppm [186,187]. Several studies on bromate formation in ozonation facilities in both Europe and the USA were summarized by Pinkernell and von Guten [188]. The findings show that most plants had bromate concentrations less than 0.010 ppm, but 6% had greater than this limit.

There are ways to minimize bromate formation, such as adding ammonia, which interferes ozone reactions with hypobromous acid, and pH depression. Both methods yield about a 50% reduction in bromate formation [188,189,190,191]. Once formed, however, bromate tends to persist, and removal is difficult [188]. RO has been shown to reject about 96–97% of bromate in ozonated water [189,190,191,192]. Other removal methods tested are described by von Guten [155]. These include activated carbon, which reduces bromate to bromide, but other anions and NOM negatively impact the efficacy of the technique. Biological activated carbon and the dosing of iron (II) also reduce bromate, but both techniques require a low-oxygen environment. Finally, UV radiation at 255 nm at a dose 100 times higher than required for disinfection will reduce both hypobromous acid and bromate.

Another concern with the use of ozone is the ozonation of humic compounds (humic and fulvic acids), yielding carboxylic acids and aldehydes [77]. This oxidation process can result in a slight, net decrease in TOC [77]. However, a net decrease in the ratio of high-molecular weight organics to low-molecular weight organics typically occurs. Thus, there is a net production of AOC following ozonation that serves to foster microbial growth [6,76,77]. Biofiltration following ozone treatment can be used to reduce AOC and also reduce some of the bromo-organic compounds described earlier [160]. Chlorine or chloramine would then need to be used downstream of the biofilter for secondary disinfection prior to a membrane system.

Ozone can, under the right conditions, decrease the potential for chloroform formation with secondary chlorination [77]. However, analyzing and interpreting results from actual systems can be difficult due to the many variables that influence efficacy. These variables include the nature of the humic substances, ozone dosage, pH, chlorine dosage, chlorine pH of application, and bicarbonates that may or may not be present [77]. Reckhow, et.al. [193] demonstrated the reduction in formation of chloroform and total organic halides at neutral pH. But this work also shows that the potential for formation of dichloroacetic acid and trichloroacetone may increase [193]. Despite this, it is believed that ozonation yields fewer undesirable DBPs than chlorination [77].

Another limitation of ozone is that its high volatility yields little or no residual ozone for preventing regrowth downstream of the contact basin. This is particularly important if the microorganisms are shielded from ozone attack by particulates in the water. This, together with the ability of some microorganisms to regenerate themselves following ozone damage, make secondary disinfection a necessity for drinking water facilities. Secondary disinfection is not practical for pretreatment of membrane systems operating on “fresh” water supplies; however, secondary disinfection (ozone followed by chlorine-based compound(s)) may be encountered in wastewater reuse applications.

Due to the extremely corrosive nature of ozone, materials of construction are limited to 316 L for the ozone generator and wet- and dry-gas piping systems, and type 2 or type 5 Portland cement for the basin structures [77]. Some plastics have good to excellent compatibility with ozone, including CPVC, HDPE, PVDF, and PTFE, while others, including polyamide and nitrile, have poor resistance to ozone and are “not recommended” for use [194].

Removal of Ozone

There are a few ways of removing ozone from feed water to membrane systems that can be more reliable than assuming all the oxidant has dissipated. UV radiation and the use of quenching compounds are the most direct and simplistic methods. UV energy at 254 nm converts ozone to water and oxygen [50]. Quenching compounds include peroxide, calcium thiosulfate, and sodium bisulfite. These stop the ozone and radical reactions [160]. Two other methods, aeration and carbon filtration, pose hazards. Aeration requires environmental monitoring of the ozone that is stripped to the atmosphere. Additionally, adsorption on carbon media can result in fires and explosions, due to the exponential reaction of ozone with carbon; this hazard is exacerbated when the ozone is generated using pure oxygen [160,195].

Hydrogen Peroxide/Peracetic Acid

Hydrogen peroxide (peroxide) and the organic compound peracetic acid (chemical formula CH_3_CO_3_H, also known as PAA) are both electrophiles/oxidizing compounds that have higher oxidation potentials than free chlorine (see Table 3). Therefore, they are damaging to polyamide membranes. PAA is an antimicrobial agent that dissociates in water to form peroxide and acetic acid [196]. Peroxide together with PAA has demonstrated successful mitigation of biofouling on polyamide membranes [197]. The solution is effective in penetrating stagnant areas within the membrane element [87]. However, the mixture of peroxide and PAA must be used under very controlled conditions [92,198]. Conditions include a solution strength of less than 0.2 wt% peroxide and an application temperature of less than 25 °C. Transition metals and hydrogen sulfide must not be present. And application pH of 3–4 is recommended for optimal disinfection.

Due to the need to very controlled application, the recommendation is to use this solution on a periodic rather than continuous basis, with membrane exposure time limited to about 20 min [197]. A solution is typically applied in recirculation/soak modes after cleaning. Cleaning should include a high pH solution to remove as much bio-based fouling as possible, followed by a low pH solution to remove any metals that may catalyze the oxidation of the membrane with the peroxide/PAA solution [197]. While a polyamide membrane is limited to 20 min of exposure to the oxidizing solution [197], work has shown that a soak of 2 h is required to provide a 90% kill and a 24-h soak is expected to kill 99% of bacteria [198] with this type of solution. Thus, efficacy of this technique is limited.

#### 3.1.3. Non-Oxidizing Biocides for Membrane Disinfection

##### 2,2-Dibromo-3-nitrioproprionamide (DBNPA)

DBNPA is a non-oxidizing biocide that can be used directly on polyamide membranes. It is a moderate electrophile that acts on the bacterial cell wall and on the cytoplasm within the cell; it does not penetrate EPS [93]. Therefore, DBNPA is most effective with new or sufficiently cleaned membranes that are relatively free of any biofouling. Research by Siddiqui et al. [199] demonstrated that a continuous dosage of 1 ppm DBNPA could prevent accumulation of biomass and the associated increase in pressure drop during a 7-day test with chlorine dioxide pretreated water and virgin membranes. However, for membranes already biofouled, Siddiqui et al. [199] found continuous dosages of 1 ppm and 20 ppm of DBNPA only inactivated the accumulated biomass; neither of these dosages removed the inactivated cells and biomass, nor restored the original system pressure drop.

**Figure 12:**
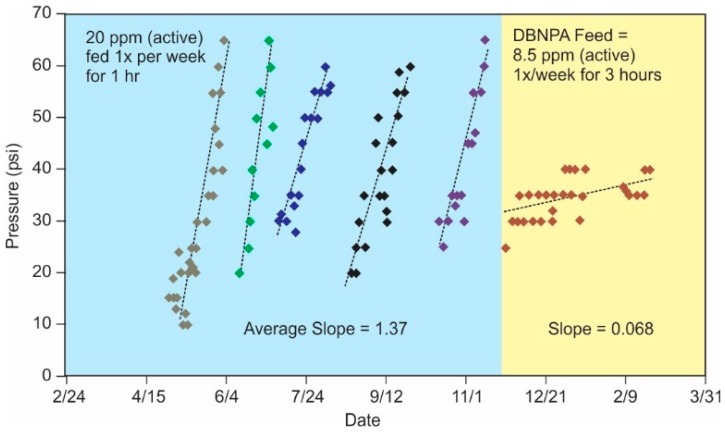
Differential pressure as a function of time for an RO system treated with conventional and a lower-dose, longer-exposure method. Surface water feed form the Weisse Elster River in Germany [93]. Reprinted with permission of the Engineers’ Society of Western Pennsylvania.

Typical application of DBNPA is either via continuous or shock treatment. Continuous treatment involves feeding 2.5–10 ppm [92] as 20% product (application at neutral pH is recommended, as DBNPA hydrolyzes a pH greater than 8 [200]. However, the expense of the product typically precludes continuous treatment. Conventional shock-dosing includes feeding 10–30 ppm as active for 1–3 h [92], every 2–3 days. Schook et al. [93] demonstrated that a shock dose 8.5 ppm as active DBNPA for 3 h a week (a lower dosage and longer exposure period than conventional shock treatment) was more effective than the generally recommended 20 ppm (as active) for 1 h once per week (see Figure 12). DBNPA can also be used to supplement a cleaning regimen via off-line recirculation, but the membranes should be cleaned to remove as much of the bacteria and biofilm as possible before dosing the product [93,199].

DBNPA is well rejected by RO membranes, up to 99.98% for seawater membranes [93]; on-line treatment is acceptable for virtually all industrial applications. However, due to the limited but discrete passage (~0.02%) of DBNPA into the permeate, potable and food and beverage applications require offline application [87,93,201]. Also, DBNPA gives an ORP reading of about 400 mV at an active concentration of 0.5–3 ppm (free chlorine yields a reading of about 700 mV). Hence, on-line use requires an understanding of the natural ORP of the feed water source and how DBNPA and free chlorine influence the site-specific measurement.

DBNPA is not recommended for use as a biocide during long-term storage due to its short half-life, less than 24 h at pH 7 and 15 min at pH 9. Decomposition of DBNPA is rapid and decomposition products are carbon dioxide, ammonia, and bromide [200,201]. Exposure to sunlight (UV) can also breakdown DBNPA to an organic compound, cyanoacetamide, an acetic amide with a nitrile functional group [200], so materials of construction and location of storage and day tanks must be considered to minimize exposure to sunlight. DBNPA is corrosive to metals, so materials exposed to high concentrations of the biocide (e.g., storage tanks and metering pumps) should be of plastic construction.

##### Isothiazolones

The conventional isothiazolone biocide used is a 3:1 ratio of 5-chloro-2-methyl-4-isothiazolin-3-one (CMIT) and 2-methyl-4-isothiazolin-3-one (MIT) [92,202,203,204]. CMIT/MIT is a moderate electrophile [202] that can enter the cell membrane by one of two methods: diffusion (when high concentrations of biocide are used thereby resulting in a more rapid kill [202]) or active transport through the membrane (at concentrations typically used in water treatment, and which results in slower kinetics of inactivation [203]) (the exact nature of the active transport is not well understood [88]). Once within the cell, inactivation of enzymes within the cell occurs via interaction of the isothiazolone with thiol groups available on specific enzymes [88]. Interference with enzymes inhibits respiration and energy generation (ATP synthesis). Cell death with isothiazolones can take several hours [203], but higher concentrations of the biocide increase kinetics of diffusion into the cell, as mentioned previously), or via the addition of other actives, such as surfactants [202]. Species that are affected by isothiazolones include aerobic and spore-forming bacteria, algae, and fungi (at pH 6.5–9.0) [92,203] Resistance of microorganisms to isothiazolones is not as common as with some other biocides [202].

Dosage rates for isothiazolones range from 0.75–1.8 ppm (or 50–120 ppm of a typical 1.5% active product solution) for 5–6 h of exposure [203]. As a result of the long contact time for isothiazolones to be effective, they are recommended for membrane layup rather than for on-line dosing. Also, isothiazolones are highly toxic to aquatic life, which can restrict use in some applications. They are recommended for off-line only treatment of potable water and food and beverage applications [92]. For desalination systems it is advised to clean the membranes of organic and other foulants prior to use, as is the case for DBNPA [92].

##### Sodium Bisulfite

Sodium bisulfite is a reducing agent typically used for dechlorination and oxygen reduction in boiler systems. Sodium bisulfite is also an antioxidant with good efficacy for inhibiting growth of aerobic bacteria as it removes oxygen from the water. It is known for being able to stop aerobic bacterial growth without removing existing colonies [50,205]. (Note, however, that anaerobic bacteria can proliferate in the presence of sodium bisulfite as the product provides an oxygen-free environment for these bacteria.)

For on-line, brackish water treatment, shock dosing using 500 ppm (as 37% active) sodium bisulfite for 30–60 min is recommended [92,205,206] using 500 ppm for 30 to 60 min of exposure time [85]; Laguna [198] recommends 500–1000 ppm for 30 min every 24 h. Continuous treatment at dosages up to 50 ppm have been used for seawater systems [50,205]. Use of sodium bisulfite as a biocide is only effective on feed water with a low to medium potential for biofouling such as well water and “clean” surface water. Sodium bisulfite is not recommended for open seawater intakes or river/lake intakes near harbors and municipal discharge [50].

Sodium bisulfite is also used to inhibit biogrowth during membrane storage [104,206,207]. Membranes should be as free of microbes as possible (usually after a cleaning) before being stored. A storage solution of 0.5% to 1% sodium bisulfite is recommended for up to about 6 months. Bisulfite scavenges oxygen from the air and reacts with it to produce sodium sulfate during the storage period. Hence, it is important to monitor the pH of the solution on a regular basis during storage to ensure it is still active for biocontrol. The solution should be replaced when its pH drops to about 3 to ensure activity of the bisulfite and to prevent damage to the membrane when stored under such strongly acidic conditions [92,207].

#### 3.1.4. Other Compounds Less Commonly Used for Membrane Disinfection

##### Ethanol

Ethanol is a lytic, membrane active agent that migrates into the hydrophobic regions of cell membranes, reducing their integrity. Under acidic conditions, the weakened cell membrane allows for protons to enter the cell, while ions, magnesium, and nucleotides leak out. Alcohols also denature proteins, thereby inhibiting cell growth [208]. Work by Heffernan et al. [209] demonstrated up to 4-log reduction in sessile bacterial populations on used membranes soaked in ethanol for 18 h. Exposure to 40% ethanol for 1.5 h was shown to effectively disinfect a DuPont-FilmTec NF90, polyamide NF membrane while not adversely affecting performance, showing virtually no loss of flux or rejection [209]. The results contradict studies by Van der Bruggen et al. [210] which found membrane swelling due to exposure to ethanol resulting in higher permeate flux. Heffernan et al. [209] note that each polyamide RO or NF membrane has a unique active-layer structure and cautions that further investigation is necessary to determine the impact of ethanol on other specific membranes makes and models.

##### Potassium Permanganate

Potassium permanganate is usually used for iron and manganese oxidation followed by filtration with greensand and pyrolusite filters or for organic fouling control rather than for direct biocontrol [173,211]. Galvin and Mellado [212] studied the use of potassium permanganate prior to polyamide membranes in a municipal seawater facility. Their work found that using a dose of 0.45 ppm to 0.8 ppm did improve membrane performance during algae blooms due oxidation of organics and the algae. The use of potassium permanganate increased the cost of water treatment by about 3–5 USD/1000 m^3^ produced in the 1998 study.

##### Copper Sulfate

Copper sulfate is known as an algicide rather than a bactericide and has limited use due to the adverse effects of copper [86]. Many countries have imposed strict limitations on concentrate discharge from RO systems using copper sulfate [86]. However, copper sulfate has been used with some success as an alternative to chlorine for large RO systems operating on the Red Sea [86].

##### Caustic

Caustic addition increases the negative potential of the polyamide membrane, resulting in greater repulsion of negatively charged microbes [213,214]. Caustic is not generally used for biocontrol for membrane desalination systems [23,215,216]. This is because the ionic concentration of typical feed water to such systems is too high for pH adjustment to be effective [215,216].

#### 3.1.5. Biocides not Generally Recommended for Polyamide Membranes

A few non-oxidizing biocides that are not compatible with polyamide membranes, including quaternary amines (quats) and aldehydes [88]. Quats, which are membrane-active biocides, and aldehydes, including glutaraldehyde and formaldehyde, that are moderate electrophiles cause irreversible flux loss [217]. Aldehydes can lead to an irreversible flux loss of up to 50% when employed with new membranes. Subsequent exposure can lead to additional flux loss that may or may not be permanent [92]. For polyamide membranes that have been on-line for at least 24 h, aldehydes can be used, but even exposure with “used” membranes can lead to flux loss, which may or may not be permanent [92]. However, due to the health hazards they pose, they are generally not used for membrane applications.

### 3.2. Non-Chemical Disinfection Techniques

To eliminate the use of chemical biocides, phytochemical, electrochemical, and other non-chemical techniques are sometimes employed. Techniques covered include UV, sonication, and electric fields. These techniques do not general DBPs but also do not offer any residual for downstream disinfection. UV is well-commercialized for disinfection, but others that are discussed herein have not yet been applied commercially.

#### 3.2.1. Photochemical Technique—Ultraviolet Radiation

##### Activity

**Figure 13:**
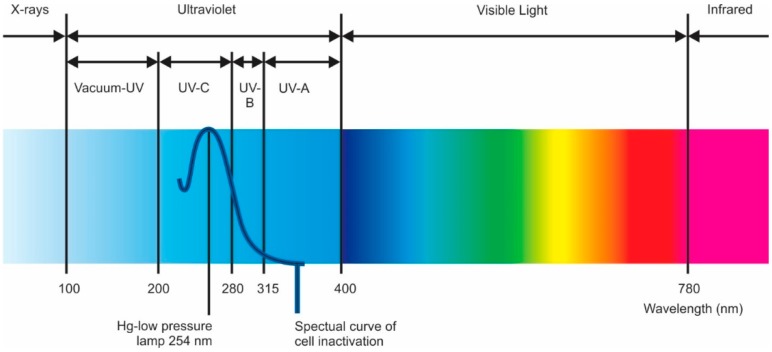
Electromagnetic spectrum with wavelength range for disinfection highlighted.

UV electromagnetic radiation ranges from 100–400 nm in the electromagnetic spectrum. There are four categories of UV radiation: UV-A at 400–315 nm, UV-B at 315–280 nm, UV-C at 280–200 nm and vacuum UV (VUV) at 200–100 nm. The UV-B/UV-C range from 200–300 nm, is where primary disinfection occurs, with maximum biocidal efficacy at 254 nm (see Figure 13).

The mechanisms of disinfection involve either direct or indirect inactivation. The process of direct inactivation involves adsorption of photons by the proteins in the cell wall with subsequent damage to the cell membrane; leakage of protoplasm leads to death of the cell [218,219]. In-direct inactivation involves the absorption of radiation by cellular nucleic acid (primarily DNA and RNA) resulting in structural damage. This renders the cell inactive and unable to reproduce [1,218,219,220,221].

##### UV Light Generation

UV light occurs when photons are emitted from gasses that are excited by an applied voltage. The specific wavelength emitted is a function of the type of gas used. The most common gas lamps for disinfection are mercury vapor and are classified as medium-pressure (MP), low-pressure (LP), or low-pressure high-output (LPHO) [220,221]. The lamps contain electrodes to generate an electric arc. A complete lamp assembly contains the lamp casing, electrodes, mercury gas, and an inert gas (typically argon). Other types of UV lamps have also demonstrated limited inactivation of microorganisms and, hence, are not as widely used [220]: xenon gas (or pulsed UV), electrode-less mercury vapor, metal halides, excimer, light-emitting diodes, and UV lasers.

There are two types of UV reactors: open channel and closed vessel. Open channel reactors feature an open basin through which the water to be treated travels by gravity flow. For closed vessels, water is pressurized as it flows through the reaction chamber.

##### Dose and Efficacy

**Figure 14:**
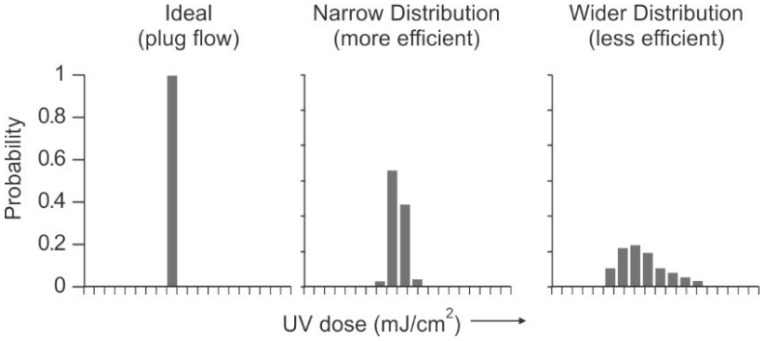
Examples of possible dosage distributions for ideal and continuous-flow UV reactors.

Intensity of UV light is a critical factor in determining the effectiveness of disinfection [221]. The intensity of light is defined by Maxwell’s equations [222]. The unit of intensity is watts per square meter (W/m^2^). If the intensity of UV light is constant, then the dose of UV light is determined by the intensity multiplied by the exposure time; if the intensity varies, then the integral of the intensity over the exposure time determines the dose. Common units for dose are Joules per square meter (J/m^2^), milliJoules per square centimeter (mJ/cm^2^), and milliwatt second per square centimeter (mWs/cm^2^). The dose a microorganism receives is a function of the degree of mixing within the reactor [221]. For a plug-flow reactor, the dose distribution is narrow, but for a continuous flow reactor, the distribution of dose can be much broader (see Figure 14). 

Due to the somewhat random motion of the microorganisms in the UV reactor, some microorganism will receive a higher dose as they pass closer to the lamp, while those further away from the lamp (such as near the reactor wall) receive a lower dose. Further, some microorganisms may short circuit though the reactor, resulting in a shorter exposure time and lower dose, while others may travel more circuitously through the reactor, with a longer exposure time and, thus, receiving a higher dose. Table 9 lists the UV dosages required for inactivation of various pathogens.

##### Maintenance

**Figure 15:**
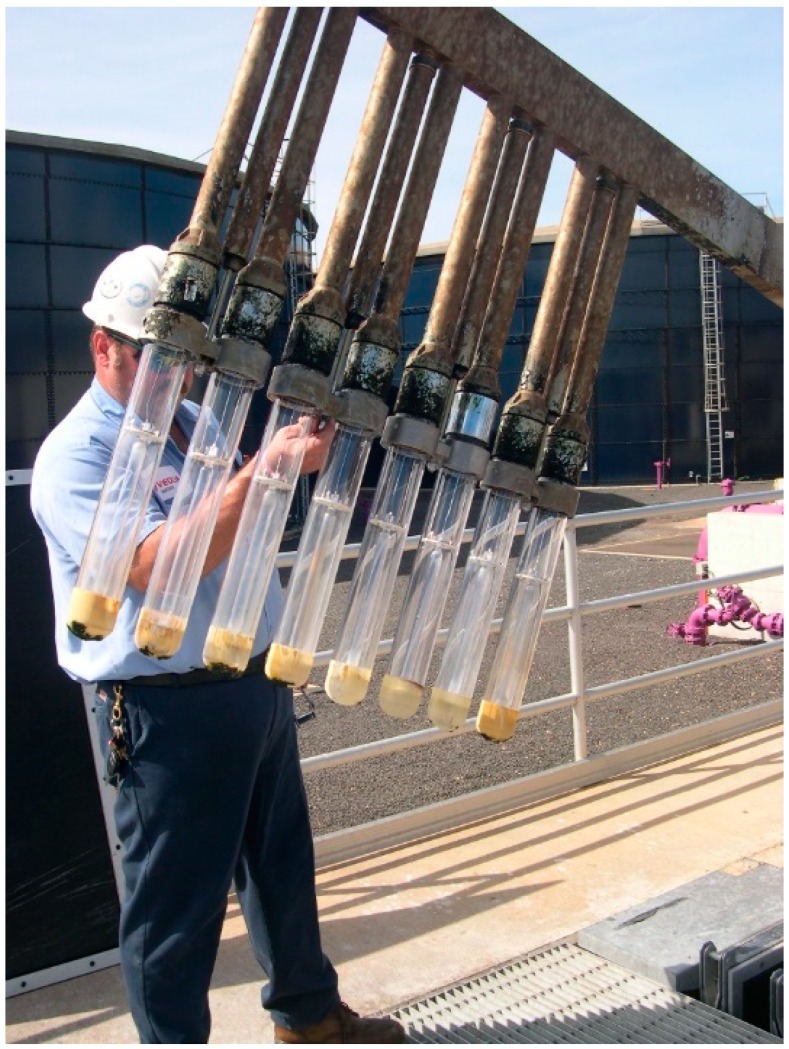
UV assembly from a basin reactor out for inspection and maintenance. Courtesy of Scrivener Publishing.

Equipment maintenance is key to optimal UV operation. Preventative maintenance is required to keep the lamps free of foulants as well as to keep the system is good operational conditions [221,223]. Refer to Hunter and Townsend [220] for a comprehensive list of weekly, monthly, semiannual, and annual maintenance requirements. Cleaning of lamp sleeves is critical. Cleaning can be conducted on-line via mechanical (OMC) or combination mechanical/chemical (OMCC) techniques. Off-line chemical cleaning (OCC) has also been practiced. The OMC and OMCC operations use wipers to physically remove particulates and organic foulants. Figure 15 shows a UV assembly removed from a basin reactor for inspection and maintenance.

##### Advantages and Limitations

UV is effective for deactivation of microorganisms in the bulk water. No harmful byproducts or residual are created by UV irradiation that adversely affect polyamide membranes. The effectiveness is not affected by pH and chemical handling is limited to chemical cleaning of the sleeves, if this cleaning method is used.

Perhaps the most important limitation of the technology is the issue of microbial repair of organisms damaged by UV radiation [96,224,225]. Two mechanisms of repair have been identified: photoreactivation and dark repair [220]. In photoreactivation, a light-activated enzyme is used to catalytically repair the damage to the microbial nucleic acids inflicted by the UV light. During dark repair, the damaged microorganisms repair themselves by multi-enzymatic clipping of the damaged strand of DNA followed by repair with a second strand of DNA. The issue of repair of microorganisms is particularly important for membrane systems following UV when used as the primary means of disinfection. The potential for regrowth suggests that higher UV dosages are required to compensate for regrowth [220], or secondary disinfection methods be employed. To minimize time for regrowth, UV systems should be in close proximity to the membrane system [219].

UV radiation has also been known to cause cleaving of DOM into smaller, more biodegradable AOC [226,227,228,229]. This stimulates microbial regrowth and biofilm formation downstream of the UV system [230]. Studies by Choi, et.al. [230] and IJepaar, et al. [231] found that the use of MP mercury lamps did increase AOC in bench- and pilot-scale studies, and in actual drinking water facilities; LP mercury lamps did not appreciably increase AOC concentration. The increase in AOC and possible regrowth of microorganisms downstream of the UV system are critical considerations for membrane systems using MP-lamp, UV radiation for pretreatment disinfection.

UV efficacy is diminished in the presence of suspended solids and turbidity and humic acids [219,232]. UV transmittance, defined as the amount of UV radiation at a given wavelength passing through a defined path length of water, typically 1–10 cm, is affected by these interferences. For UV disinfection, UV transmittance at 254 nm (UVT_254_) is the design basis for most systems. Table 10 lists general UVT_254_ for various water sources [220]. Additionally, particulates surrounding microorganisms in water can completely block the transmittance of UV light to the organisms, further reducing the efficacy of the disinfection process [233].

The cost for UV generation is more than chlorination [96]; however, total life cycle costs may be competitive, in some cases, for membrane pretreatment, if removal costs of chlorine-based disinfects are considered. UV operational costs include power consumption and maintenance items (cleaning chemicals, equipment repairs, and lamp/sleeve replacements [221]. Cost is also dependent on feed water quality, due to transmittance issues and the need to design the UV system to compensate for lower transmittance with lower-grade feed water sources [96].

#### 3.2.2. Physical Methods

##### Ultrasound

Ultrasound (sonication) is a chemical-free process that uses acoustic cavitation to cause bacterial cell disruption. Acoustic cavitation produces bubbles that introduce turbulence into a solution. The turbulence leads to rupture of the microbes. The bubbles also generate pressure differences during formation and bursting that can also result in damage to the microorganisms [32]. The technique is still under research for use with membranes; it is considered for substitution for UV and chlorine disinfection [23].

##### Thermosonication

Thermosonication (ultrasound plus heat) is being studied by Al-Jaboori, et al. [232] for biological control of RO membranes in a batch process. This study used sonication at 48 °C to disturb microorganisms such that they could not form adequate EPS; fewer and less aggregated colonies were formed as compared to untreated feed water [232]. Specifically, the study found that an intensity of 21.5 W/cm^2^ applied for 4 min (dose of 5160 J/cm^2^) eliminated almost half of the 10^6^ CFU/mL of *E. coli* bacterium and damaged more than 10% of the surviving cells [232]. The technique has only recently been studied, and results indicate the need for further research on efficacy and feasibility for operational membrane desalination systems [232].

#### 3.2.3. Electrochemical Methods

The electrochemical methods involve either direct electrolyzers or mixed oxidants for treating biologicals in water. These methods require that an electrical field is generated. The electric field can directly damage cell membranes, leading to death of the microorganism [23]. The electric field can also create oxidizing species that affect the microorganisms [23]. A limitation of this technique is that mutagenic compounds can also be created in the water being treated [37]. Additionally, this method yields no residual bicode to address microbes that pass through the field untouched [37]. Pretreatment is also required to minimize fouling of the cathodes [37]. These techniques are in their early stages of research with polyamide membrane systems [234].

#### 3.2.4. Biological Methods

Biological methods are used to inhibit or alter how biofilm forms. Sample biologics that can be used include enzymes, bacteriophages, and signaling molecules [235]. They have low toxicity and are biodegradable which may make them attractive for some applications [235]. Some proteolytic enzymes (protease) have been shown to disperse established biofilm and to inhibit further biofilm formation [235]. Such enzymes include proteinase K, trypsin, and subtilisin [236,237]. However, enzymes are unstable and sensitive to environmental conditions, e.g., pH, temperature, and high ionic strength, thereby limiting their large-scale application [235].

Goldman, et al. [238] demonstrated a 40% reduction in bacterial attachment on a UF membrane used in a membrane bioreactor could be achieved using a phage (a virus that that infects the host bacterium and inactivates the bacterium by cell lysis or incorporation into DNA for cell disruption [239]). The general use of bacteriophages for large-scale membrane pretreatment poses challenges, in that the phage needs to be specific for the populations of bacteria present [235,238].

Signaling molecules, known as autoinducers (AI), play a role in the quorum sensing that microorganisms use to coordinate communal behaviors, such as biofilm formation, swarming, motility, and the and the formation of EPS [240,241]. Yeon, et al. [242] demonstrated membrane biofouling on a microfiltration membrane could be removed by the addition of a specific AI. Most studies using AIs, however, have been limited to lab-scale, pure cultures [235]. Significantly more study is required in real-world applications to determine the applicability of this technique for membrane biofouling control.

## 4. Membrane Cleaning and Storage

Membrane cleaning is generally used after-the-fact in an attempt to detach and sweep away adhering microorganisms and ESP that has already formed on the membrane. Cleaning does not include disinfection of the membrane. Disinfection cleaning would require the use of chemicals, specifically oxidizers, that destroy the bacteria but are harmful to polyamide. Hence, cleaning focuses on *removal* of biofoulants rather than disinfection. Preventive cleaning is not usually practiced, but research demonstrates that cleaning before biofouling become severe enough to affect membrane performance can result in fewer actual cleanings over the lifetime of the membrane [243]. As each cleaning stresses the membrane due to combinations of high temperature and pH [244], fewer cleaning events could lead to longer membrane life [12]. Also of importance is off-line flushing to keep membrane clean during short-term idling and when membrane will be off-line for more than a few days.

### 4.1. Reactive Cleaning

Cleaning membranes is usually performed based on an observed decline in performance, which manifests as a decrease in normalized permeate flow (NPF), an increase in normalized differential pressure (NdP), or, sometimes, an increase in normalized salt passage (NSP). Generally-accepted guidelines indicate that when the NPF decreases by 10–15%, or the NdP and/or NSP increase by 10–15%, a cleaning should be conducted [197,243,245]. Cleaning later than recommended has been shown to result in more frequent cleanings overall and subsequent shorter membrane life due to membrane damage that occurs under cleaning conditions [243,246,247,248].

Membrane cleaning involves chemical and physical interactions [249] in an attempt to remove accumulated biofouling. Chemical reaction between the cleaning chemical and foulant is necessary as is the mass transfer and sweeping away of the foulant removed by chemical means into the bulk cleaning solution. Key parameters in for successful membrane cleaning focus on the characteristics of the cleaning solution (pH, ionic strength, concentration, and temperature) and the physical nature of the cleaning (crossflow velocity, duration, frequency, and pressure) [245].

High pH (12+) (17) and moderate temperature (35 °C) [197,250] are necessary to effectively clean biofouling from membranes. Temperatures higher than 35 °C at a pH of 12 (or higher) is not recommended due to the potential for hydrolysis of the membrane fabric backing [244]. High pH cleaners typically include detergents and/or chelants to help penetrate the biofilm for more effective removal [7].

Two-phase cleaning (gas and water) can increase shear forces to necessary to dislodge foulants [251]. Recent research by Chesters, et al. [243] demonstrated that cleaning together with air scour (using a microbubble generator) improved flux after cleaning by 52% overusing the same cleaner without air scour. The cleaning was not able to restore flux to membrane specification, however (it is not clear if the elements tested were cleaned on time, according to the guidelines, which could account for the incomplete restoration of flux). Rietman [252] has also demonstrated effective removal of biofilm with two-phase cleaning using carbon dioxide and water.

The drawback to reactive cleaning is that membrane biofouling may involve *irreversible* adhesion of microbes to the membrane [253,254,255]. Cleaning after-the-fact can only achieve minimal success for biofouling control and mitigation.

### 4.2. Preventative Cleaning—Direct Osmosis-High Salinity Cleaning

Direct osmosis-high salinity (DO-HS) cleaning is used for preventative cleaning of polyamide membranes [256]. This is an on-line technique, during which the system feed water is spiked with high concentration of sodium chloride (25% with an osmotic pressure of 194 bar). The high osmotic pressure of the feed solution causes the membrane permeate to flow backward through the membrane via direct osmosis. The osmotic shock results in the death of microorganisms, which are subsequently lifted off the membrane surface by the direct osmosis of permeate. This DO-HS cleaning is conducted on a frequent basis to minimize the time under which the system operates in observed performance decline. Qin et al. [257] found that daily injections of the salt solution were necessary to reduce the rate of membrane biofouling.

### 4.3. Membrane Flushing

During operations, membranes not only generate pure water permeate, but also concentrate up dissolved solids and other foulants that serve to enhance the rates of fouling, including biofouling, and scaling when the system is off-line. When a membrane system goes off-line, with no crossflow water serving to scour the membrane surface, fouling and scaling rates can increase precipitously [258], leading to accelerated biofouling, given the synergistic effects general foulants have on biofouling [15]. Flushing the membranes upon shut down removes the high concentration of potential foulants and scale before a condition favorable for growth of microbes and biofilm is exceeded [258,259]. Intermittent flushing of system during stand-by is also recommended [83,258]. RO permeate-quality water or better, free of any pretreatment chemicals, should be used for flushing membranes. High crossflow velocity, up to 170 L/m per 20.3 cm diameter pressure vessel, at pressures less than 4 bar, should be used [83].

### 4.4. Membrane Storage

Membrane storage conditions are critical to minimizing biogrowth while off-line [83]. Conditions inside a membrane element without flow are ideal for biofouling of the membranes: stagnation, relative warmth or warming conditions [260], moisture, and any foulants and scale already on the membrane to promote synergistic biofouling [15].

For short-term lay-up (less than 1 to 2 weeks), routine flushing of the membranes is sufficient. A flush of the feed side of the membranes with permeate better quality water every 4 to 24 h is recommended [261]. Flushing frequency depends on the ambient temperature and nature of the feed water, with higher temperature and lower feed water quality necessitating shorter intervals between flushing.

Longer-term storage will require cleaning of the membranes prior to storage [83,197,261]. The usual preservation chemical used after the cleaning is sodium metabisulfite [204]. A 0.5%–1.0 wt% solution is recommended. Sodium metabisulfite readily oxidizes, so long-term storage should be under air-tight conditions. Exposure of sodium bisulfite to oxygen in air will oxidize it yielding sulfuric acid [197]. The pH drops to 3, the bisulfite solution should be replaced to prevent damage to the membranes [197]. Further, oxidized product acts as an ideal nutrient for anaerobic bacteria [204]. Because sodium bisulfite hydrolyses at alkaline pH (8) [207], a slightly acid pH is recommended for storage [204].

Majamaa et al. [204] conducted a comprehensive, long-term study of DBNPA and isothiazolone (CMIT/MIT) as compared to sodium metabisulfite for membrane storage and preservation. While DBNPA is a fast-acting biocide that hydrolyses at high pH, it is stable at pH less than 5 and does exhibit preservation efficacy under this condition [204]. During the Majamaa et al. [204] study, twelve used polyamide membranes, both seawater and brackish water that had been in operation in industrial plants, were used. They were cleaned with a high-pH cleaner prior to preservation testing and then preserved with DBNPA (at pH 5), CMIT/MIT, or sodium metabisulfite for up to 12 months. Membranes were tested for flux, salt rejection, and differential pressure after cleaning, and after 1, 3, 6, and 12 months of storage. The salient result of this investigation is that the performance of the membranes during preservation were very similar for all storage solutions, and without significant increases in differential pressure, which was determined by the researches to indicate good efficacy at controlling biogrowth on the membranes. An economic evaluation of DBNPA, CMIT/MIT, and sodium metabisulfite was also conducted. Table 11 shows the results of this economic study. Different preservation times for each preservative were calculated based on the activity of the specific preservative. The isothiazolone product, CMIT/MIT, was found to be the most economical preservative due to its low dosage requirement, while the standard preservative, sodium metabisulfite, was least cost effective due to the high concentration of chemical required and shorter preservation time.

## 5. Future Prospects

As a result of the limitation of current biofouling control techniques, other methods are being developed. Membrane modification shows promise for minimizing biofouling to either eliminate the need for chemical biocides or to work in concert with oxidizing biocides that may directly contact the modified membrane. New and existing biocides, including oxidizers, are being developed and tested for use with polyamide membranes. Finally, preventive cleaning is being investigated to minimize or eliminate biofouling before performance of the system declines.

### 5.1. Membrane Modification

Modification of polyamide membranes has been investigated ever since the development of the interfacially-polymerized membrane by Cadotte. Researchers have explored modifications with the goals of higher flux, higher salt rejection, higher permeability, greater resistance to chlorine, and greater resistance to biofouling [163,262,263]. The objectives for biofouling control are to minimize the factors favoring polyamide membrane biofouling. Membrane surface morphology (e.g., smoother surface), a more negatively-charge membrane, and a more hydrophilic membrane are sought. Modification techniques investigated involve surface coating, polymer blending and grafting; and inclusion of nanoparticles. Modifications to polyamide membranes to improve bioresistance while maintaining flux and rejection performance can involve coating of the membrane using polymers, anti-microbial compounds, nanoparticles, and inclusion of nanoparticles as an integral part of the membrane itself.

#### 5.1.1. Surface Modifications

To minimize bacterial adhesion, modification of membrane surface characteristics has been researched [264,265,266,267] and a comprehensive review was conducted by Nguyen, et al. [6]. Surface modification attempts to give the membrane bacteriostatic properties by smoothing the membrane surface, making the membrane more hydrophilic, and/or preparing membranes with a greater negative charge so at to repel negatively charged microorganisms [6]. Techniques to reduce surface roughness include coating the membrane with polymers or surfactants [265,266]. Other techniques to create biostatic surface characteristics include polymer blending and grafting [6].

Wang et al. [268] used a commercial polyamide membrane coated with polyacrylic acid and tobramycin (TOB—a strong anti-microbial agent) in a bilayer configuration. Assembly of the bilayer coated membranes involved exposing the polyamide membrane to 0.5 ppm TOB for 15 min, rinsing with deionized water, then exposure to 1.0 ppm polyacrylic acid for 15 min, followed by a final rinse with deionized water; this procedure constituted one bilayer. A 10-bilayer coated membranes exhibited increased hydrophilicity (contact angle less than 20°) and decreased surface roughness (average roughness 62.8 nm versus 81.6 nm for the uncoated membrane); contact angle and surface roughness both decreased with increasing number of bilayers.

**Figure 16:**
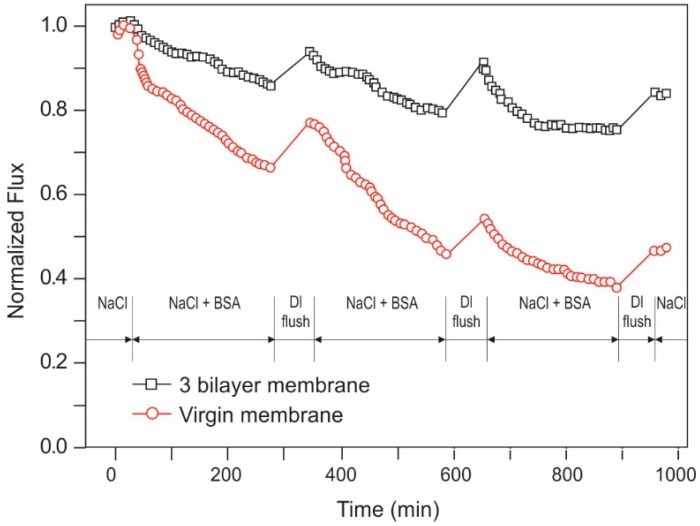
Normalized flux of un-coated and 3-bilayer-coated polyamide membranes, as a function of time, when exposed to solution of 100 ppm BSA in 2000 ppm NaCl. Cleaning of the membranes involved washes with deionized water only. Conditions: pH = 7.0 ± 0.2. Temperature and pressure where not provided. [268]. Reprinted with permission of Elsevier B.V.

**Figure 17:**
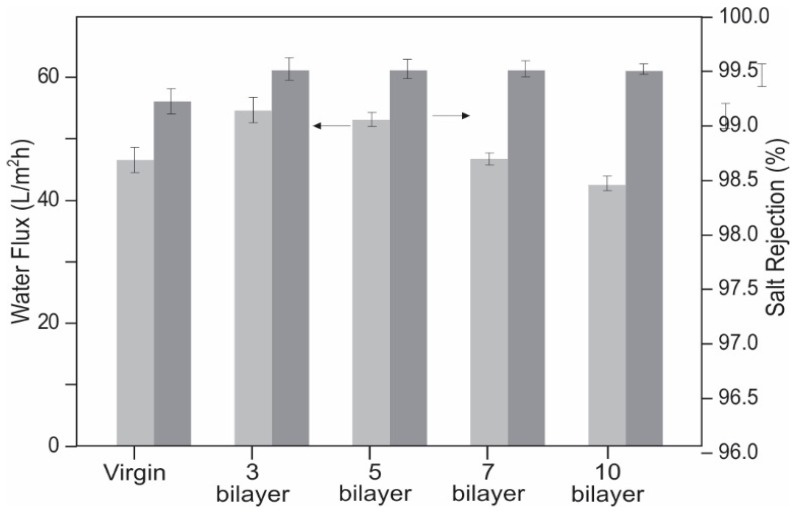
Water flux and salt rejection of 3-bilayer-coated polyamide membranes compared to un-coated membranes. Conditions: NaCl: 2000 ppm; pressure: 15.5 bar; temperature 25 ± 0.5 °C. pH was not provided. [268]. Reprinted with permission of Elsevier B.V.

Figure 16 shows the normalized flux of the uncoated polyamide membrane and a 3-bilayer coated membrane as a function of time when exposed to a solution of bovine serum albumin (BSA) in sodium chloride. The improved biofouling resistance due to the modified surface characteristics is evident. Figure 17 demonstrates the performance of the un-coated polyamide membrane and coated membrane as a function of number of coatings. Wang et al. [268] attribute the increase in salt rejection for the 3- and 5-bilayer membranes to sealing of minor defects in the un-coated membrane; increase flux is assumed to be a result of the higher membrane hydrophilicity. These coated membranes also exhibited significant anti-microbial properties over the uncoated membrane, due to the inclusion of TOB in the membrane coating, as shown in Table 12.

Other researchers have found variable results with surface coatings in terms of sustained bioresistance and flux/rejection performance [268,269,270]. In particular, Miller et al. [269] found that polyamide NF membranes with a hydrophilic coating (polydopamine and poly (ethylene glycol)) showed significant short-term reduction in adhesion of BSA and *Pseudomonas aeruginsa* during static tests. However, they concluded that short-term tests were not reliable in predicting biofouling resistance. No reduction in biofouling was observed during longer-term, continuous flow tests [269].

Nguyen [6] describes some distinct disadvantages of surface coatings, including mechanical and chemical stability and delamination during chemical cleaning. Further, not all attempts to modify membranes via coatings to change charge or hydrophilicity are always successful at minimizing all fouling of the membrane [34]. For example, increasing the negative charge of polyamide membranes to reduce the potential for bio-adhesion, can increase the potential for fouling with positively charge species [268]. And, increasing the hydrophilicity of the membrane can result in fouling with hydrophilic species, such as some components of NOM [271].

Nguyen [6] discusses issues with other surface modification techniques, such polymer miscibility and as long-term stability of the modified surface (polymer blending technique). Also discussed are grafting techniques that result in changes to membrane chemistry that alter membrane performance, such as permeability [6]. Despite the limitations of coatings, initial successes merit additional investigation into coating formulations and coating techniques to enhance long term stability and bioresistance, as well as flux/rejection performance.

#### 5.1.2. Use of Nanoparticles

Nanoparticles have also been investigated as a means of improving the resistance of polyamide membranes to biofouling [270]. Most investigations focused on hydrophilic nanoparticles to increase permeability. Some studies included antimicrobial nanoparticles like silver [272] and chitosan [273], and other inorganic additives, such as titanium dioxide nanoparticles [274,275], silica [276], zirconium dioxide [277], and alumina nanoparticles [278] to improve bioresistance. Manjumeena et al. [279] conducted research into coating conventional polyamide membranes with silver nanoparticles (AgNP) to impart antibacterial features to the membranes. These AgNP-coated membranes demonstrated good antibacterial properties against strains such as *E. coli*, *M. Luteus*, and *K. pneumonia* [279]. Emadzadeh et al. [280] found that polyamide membranes that incorporated halloysite (titanite) nanotubes exhibited higher tolerance to organic fouling than standard polyamide membranes, and what organic fouling did occur, was highly reversible [281]. Lower tendency for organic fouling will presumably reduce the potential for biofouling of the membrane (work by Dudley et al. [282] found that autopsied membranes with significant biofouling also contained a higher percentage of organics (greater than 60%) in the total foulant).

Polyamide membranes incorporating *hydrophobic* zeolitic imidazolate framework-8 (ZIF-8) were found to increase the hydrophilicity of the resultant nanocomposite membrane [283]. Surface roughness of the resultant thin-film nanocomposite (TFN) membrane varied from between 0% and −13% of that for a polyamide membrane, with the greatest decrease for ZIF-8 loadings of 0.05 to 0.10 wt/vol %. The corrected contact angle decreased with increasing ZIF-8 loading from 65° for the polyamide membrane to as low as 51° for the TFN membranes at 0.10 and 0.20 wt/vol % loadings. The smoother surface and smaller contact angle for the ZIF-8 based TFN membranes show promise for reducing the probability for biofouling. These membranes also exhibited higher permeability than some TFN membrane using hydrophilic zeolites with comparable rejections. The higher permeability was assumed to be due to lower attraction of water to the hydrophobic ZIF-8 pore wall, rather allowing the water to pass through unhindered [283].

Overall, results of the investigations described above with TFN membranes were mixed, however, and can have unintended negative effects on overall performance as compared to commercial polyamide membranes. None of these TFN membranes are currently commercialized.

**Figure 18:**
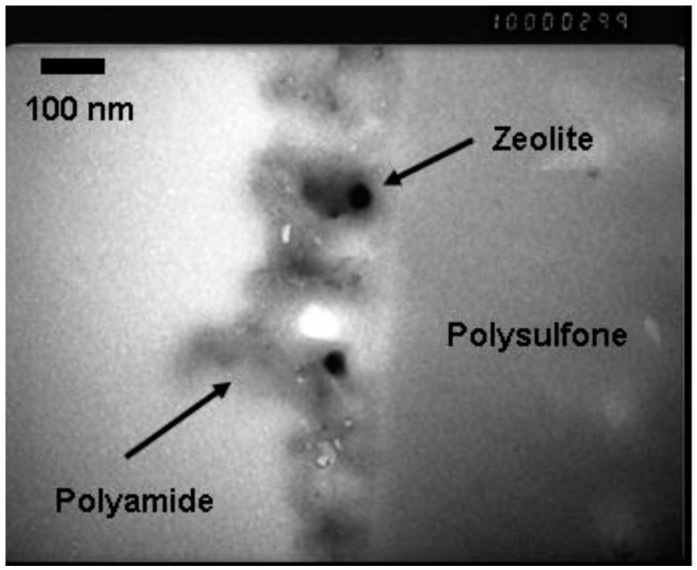
TFN membrane showing zeolite Linde type A imbedded into the polyamide membrane layer [284]. Reprinted with permission of Elsevier B.V.

One TFN membrane that is commercially available exhibits high flux and rejection (particularly for the seawater version of the membrane, LG Chem’s *QuantumFlux* (El Segundo, CA, USA) that exhibits a 99.89% rejection of sodium chloride). These membranes incorporate a hydrophilic zeolite (Linde type A, an aluminosilicate molecular sieve), into the organic phase of the interfacial polymerization reaction, yielding the zeolite in the polyamide membrane layer (see Figure 18). Work by Jeong et al. [284] indicated that high loadings of the zeolite resulted in higher water permeability when compared to TFC membranes, with equivalent rejection. The bioresistance of these commercial membranes has not yet been determined.

Despite some improvements in membrane performance, membrane modifications using nanoparticles have yet to generate a membrane that has both high productivity and salt rejection, with enhanced resistance to biofouling and to chlorine (and other oxidants). More research is necessary to further investigate techniques and methods of membrane preparation to attempt to achieve truly effective, bacteriostatic membranes [6,37,272,273] that do not enhance other types of fouling, and that also retain polyamide membrane performance. Additional review discussions on this topic are provided by Misdan et al. [285] and Kang and Cao [286].

#### 5.1.3. Graphene Oxide (GO) Composite Membranes

Research has been conducted over the last 3 to 5 years with GO thin film composite membranes for higher productivity, resistance to biofouling, and chlorine tolerance [287,288,289,290,291,292]. GO-based membranes are generally classified as polyamide surface coatings or embedded in the polyamide layer as a “nanofiller” [293]. Various preparation methods have been employed to develop GO-based membranes, including embedding GO in the polyamide layer via dissolution in the aqueous, m-phenylenediamine (MPD) solution [292], covalently-bounded surface grafting using azide-functionalized GO (AGO) [290], spin-coating [289,291], and layer by layer coatings on the polysulfone layer prior to forming the polyamide layer [294]. Many of the coating techniques result in a loss of permeability as compared to a commercial polyamide membrane [292,293,295]. But, GO-embedded membranes reported by Ali et al. [287] and Chae et al. [292], exhibited permeabilities on a par with virgin polyamide membranes (at brackish water conditions, 15 bar and 2000 ppm NaCl solution). Still, salt rejection was found to lag commercial polyamide membranes, with reported values ranging from about 96% [287] up to about 99.4% [292] (versus 99.5+% rejection for most commercial polyamide brackish water RO membranes).

Table 13 shows the performance of various GO membranes (adapted from Ali et al. [287]). The GO membrane performance presented by Ali et al. [287] is compared to commercially available polyamide composite seawater and brackish water RO membranes as well as commercial seawater and brackish water TFN membranes. The permeability for the commercial polyamide brackish membrane shown in the table is 3.5 L/m^2^-h/bar. This permeability is 1.8 times higher than the highest permeability for the GO brackish membranes listed (2.0 L/m^2^-h/bar), contrary to the findings of Ali et al. [287] and Chae et al. [292]. The same holds true to the seawater GO and PA membranes listed in the table. However, the GO seawater membrane, with a permeability of 0.52 L/m^2^-h/bar, exhibited slightly higher permeability than the seawater nanocomposite membrane, with a permeability of 0.47 L/m^2^-h/bar. The GO seawater membrane suffered significantly higher salt passage, as was noted by Ali et al. [287] and Chae et al. [292] in comparing GO and commercial membranes at brackish water conditions. In fact, all GO membrane rejections in Table 13 are shown to be well below the 99.6+ percent rejection exhibited by the commercially available membranes in the table.

While permeability and rejection are lacking for current GO membranes, additional research by Chae et al. [292] using GO embedded within the polyamide layer found that the resultant membranes are more hydrophilic, more negatively charged, and smoother than commercial polyamide membranes, making them less likely to foul with microbes. Huang et al. [290] found the same membrane properties for their AGO membranes, while and Ali et al. [287] found a rougher surface for their GO embedded membranes, presumably due to aggregation of GO within the polyamide matrix as described in the text (no data in terms of AFI or average surface roughness was provided). Table 14 describes characteristics and performance of the various GO membranes described herein.

There are some interesting trends in the data shown in Table 14. Regarding overall membrane performance, greater GO content appears to increase permeability as compared to virgin polyamide membranes, while rejection showed mixed results with changes in GO content; rejections still approximated those of the virgin polyamide membranes. Regarding membrane characteristics, contact angles for GO-functionalized polyamide membranes are smaller, and in some cases, significantly smaller, than for the virgin polyamide membrane. And, the higher the GO concentration in the preparation, the smaller the contact angle, indicating that the GO membranes are more hydrophilic. The surface roughness also decreases with increasing GO content, that can minimize fouling of any sort. Zeta potential becomes more negative for increasing GO content (in the study by Chae et al. [292]). The triple effect of increased hydrophilicity, smoother membrane surface, and greater negative charge relative to polyamide membranes could significantly reduce the potential for biofouling of these GO-functionalized polyamide membranes.

**Figure 19:**
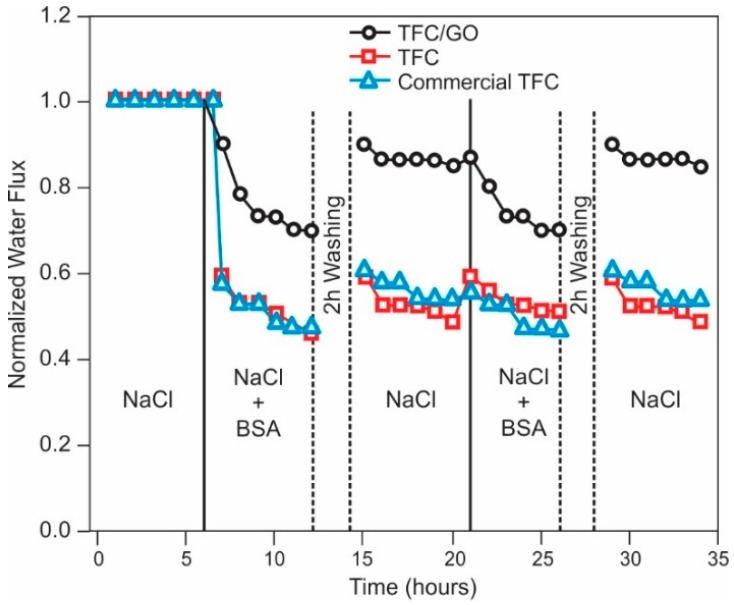
Water flux (normalized to initial water flux) as a function of operating time for GO-embedded polyamide membrane (TFC/GO), house-prepared polyamide membrane (TFC) and a commercially available polyamide membrane (Commercial TFC). Feed solution contained either pure NaCl at 2000 ppm, or 100 ppm BSA + 2000 ppm NaCl. Membrane washing was conducted using water only. Feed temperature, pressure, and pH were not reported. [287] Reprinted with permission of Elsevier B.V.

Indeed, GO composite membranes show marked improvement in resistance to biofouling compared to commercial polyamide membranes [287,290,292]. Huang et al. [290] found a 170% reduction in AGO composite membrane fouling with *E. coli* after 24 h of exposure, compared to the commercial polyamide membrane. Ali et al. [287] also found improved resistance to biofouling, as shown in Figure 19, albeit the rougher surface, attributing this to the GO-embedded membrane’s higher hydrophilicity and greater negative surface charge. Perreault et al. [296] found that *E. coli* viable cell counts using a composite membrane with a GO nanosheet covalently bonded to the polyamide membrane layer were 35.5% of the counts for a polyamide membrane after 1 h of exposure. GO deactivates bacteria on direct contact via cell damage and result loss of cell integrity [297]. This biocidal effect is greater for GO-coated polyamide membranes than for GO-embedded polyamide membranes due to the greater direct access of microbial cells to the GO [296].

**Figure 20:**
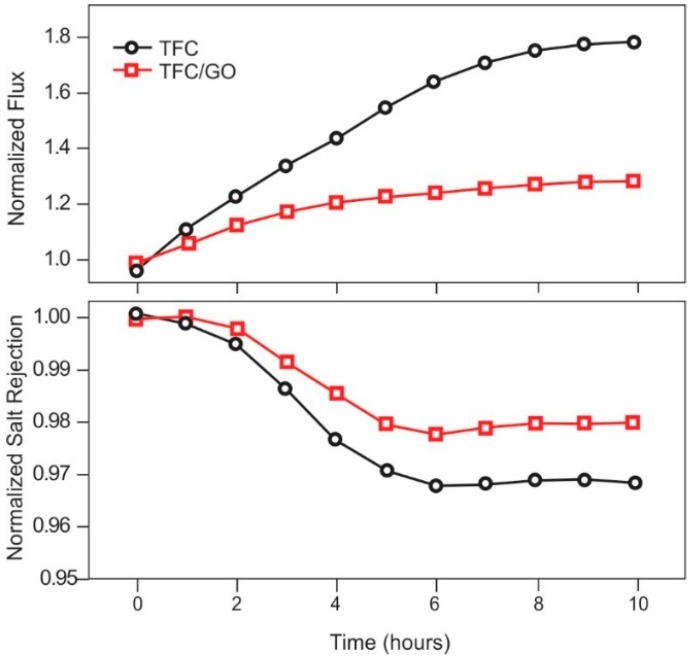
Water flux and salt rejection, normalized to initial performance, for a GO-embedded PA membrane (TFC/GO) and a house-prepared polyamide membrane (TFC) as a function of exposure time to a 500-ppm solution of chlorine. Conditions: 7 bar, 20 °C, pH = 7.0, and 2000 ppm NaCl [287]. Reprinted with permission of Elsevier B.V.

Biofouling was not completely abated in any of the testing using GO composite membranes [287,290,292,296,297]; Figure 19 is representative of fouling performance for GO embedded polyamide membranes [287]. Hence, current development status requires that chlorine or other oxidant be used to supplement the membrane performance. GO composite membranes have demonstrated enhanced resistance to chlorine over polyamide membranes [289,292,298]. The proposed mechanism of resistance to chlorine based on the strength of hydrogen bonding between GO and polyamide that hinders replacement of the amidic hydrogen with chlorine (the initial step in chlorine oxidation of a polyamide membrane) [290,292]. While the tolerance to chlorine is greater for the GO membranes than for commercial polyamide membranes, the resistance is not 100%. Figure 20 shows the effects of chlorine exposure for a polyamide membrane embedded with GO to that of an un-modified polyamide [287]. As shown in the figure, the control doubles salt passage in 1000 ppm hours of exposure to chlorine, which is consistent with the consensus regarding the degree of damage to a polyamide membrane [83] (most membrane manufacturers cite 1000 ppm-hours of chlorine exposure is tolerable before salt passage doubles [3,143]). The GO-embedded membrane also showed decline the salt rejection, but the loss was not as significant as for the virgin polyamide membrane. Both membranes also showed an increase in flux, but, again, the effect on the GO-modified membrane was not nearly as substantial.

Suggested areas of additional research involving GO membranes include selecting promising GO preparation techniques for desalination applications which can improve on the current state of solute rejection, permeability and resistance to biofouling and oxidants. Additionally, establishing best practices in these promising preparation techniques, and resolving conflicts in reported permeabilities of GO-modified membranes compared to that of commercial polyamide membranes are needed. Most current efforts are in the R&D stage, but scale-up to pilot-sized and larger systems will be necessary before commercialization is possible [293]. A review by Jiang et al. [293] provides additional details on GO, GO membrane preparation techniques, and current/future challenges.

### 5.2. Chlorine Dioxide

The use of chlorine dioxide gas has increased considerably since 1990–2000. It is an oxidizing biocide used for disinfection as well as color, taste, and odor control [299,300]. It is a very effective biocide as it, unlike chlorine, remains a gas in in water so it easily penetrates biofilm to attack the underlying bacteria. Hence the interest in using it for membrane disinfection. And, chlorine dioxide oxidizes THM precursors thereby yielding fewer HAA and THMs than chlorine [301,302,303,304]. Volk et al. [305], reported that the substitution of chlorine dioxide for chlorine in distribution systems reduced the concentration of THMs by 81%.

However, the question of compatibility with polyamide membranes is at issue if membrane disinfection is to be realized. As described herein, research is inconclusive and at times contradictory about compatibility. Moreover, chlorite and chlorate, byproducts of chlorine dioxide generation, application, and/or degradation, are oxidizers themselves that must also be considered.

#### 5.2.1. Chlorine Dioxide Generation

Chlorine dioxide in water treatment is used as a dissolved gas in water. The highly volatile and explosive nature of the chlorine dioxide necessitates on-site generation of the product. Generation usually involves the oxidation of sodium chlorite (NaClO_2_) or sodium chlorate (NaClO_3_) (Equations (24)–(28)). Equations (24) and (25) represent the conventional generation methods using chlorine gas or hypochlorous acid with sodium chlorite. These processes are approximately 95–98% efficient in converting chlorite to chlorine dioxide [306]:

2NaClO_2_ + Cl_2_ → 2ClO_2_ + 2NaCl (24)

2NaClO_2_ + HOCl + HCl → 2ClO_2_ + H_2_O +2NaCl (25)

Chlorine dioxide generated using Equations (24) and (25) can contain unreacted chlorine or hypochlorite that will degrade polyamide membranes, as noted by DuPont [307]: “The recommendation is to not use chlorine dioxide with FILMTEC membranes. FILMTEC membranes have shown some compatibility with pure chlorine dioxide. Chlorine dioxide that is generated on-site from chlorine and sodium chlorate, however, is always contaminated with free chlorine that attacks the membrane.”

There are other generations methods that do not rely on free chlorine that need to be employed for possible use with polyamide membranes. Chemical reactions (Equations (26) and (27)) using sodium chlorite and acid as reactants can be used. However, conversions are only about 80% efficient [308]. Chemical reactions (Equation (28)) involving sodium chlorate, sulfuric acid, and hydrogen peroxide as reactants have also been employed. Electrochemical reactors (Equations (29)–(31)) and catalytic generators (Equation (32)) have been used to minimize the use chemical reactants:

4NaClO_2_ + H_2_SO_4_ → 2ClO_2_ + HCl + HClO_3_ + 2Na_2_SO_4_ + H_2_O (26)

5NaClO_2_ + 4HCl → 4ClO_2_ + 5NaCl + 2H_2_O (27)

2NaClO_3_ + H_2_O_2_ + H_2_SO_4_ → 2ClO_2_ + NaSO_4_ + O_2_ + 2H_2_O (28)

○ Anodic oxidation of sodium chlorite:

Half reaction:

NaClO_2_ → ClO_2_ + e^−^ + Na^+^ (29)

Overall reaction:

2NaClO_2_ + 2H_2_O → 2ClO_2_ + H_2_ + 2NaOH (30)

○ Cathodic reduction of sodium chlorate:

Half reaction:

NaClO_3_ + 2H^+^ + e^−^ → ClO_2_ + H_2_O + Na^+^ (31)

○ NaClO_2_ is passed through a strong acid cation exchanger to yield

H^+^ + ClO_2_ (32a)

5H^+^ + 5ClO_2_^−^ → 4ClO_2_ + H^+^ + Cl^−^ + H_2_O (via catalyst) (32b)

While conversion of precursors to chlorine dioxide is important economically, conversion is not the same as product purity, which is important for high efficacy and to limit byproduct formation [308]. Less than 100% conversion efficiencies associated with chlorite-chlorine based generation systems (Equations (24) and (25)), for example, can lead to operators increasing the amount of chlorine reactant to the point where the excess, unreacted chlorine passes into the product, especially for systems not using gas eduction [308]. In this case, product purity is reduced due to the presence of chlorine. The chlorine can react further to form chlorate, an additional impurity in the chlorine dioxide solution [308].

For chlorate-acid based generation systems, high conversion is also not consistent with high product purity [309]. The optimized chlorine dioxide product rate law (empirical—Equation (28));

*R*_ClO2_ = 4.4 × 10^12^exp(−12230/T)*[H_2_SO_4_]^4.4^[NaClO_3_]^1.3^[H_2_O_2_]^0.6^ (33)

is not stoichiometric, suggesting that there are side reactions occurring [310], which lead to product impurity. Specifically, the product will be acidic, containing sulfuric acid, as well as unused peroxide. In the case of an upset in the delicate peroxide/sulfuric acid ratio of the reactants, the product may be very acidic [310]. And, perchlorate ion, ClO_4_^−^, as well as chlorine gas, is generated from chlorate under very acidic conditions (commercial sodium chlorate solution can also contain up to 200 ppm perchlorate as an impurity, as shown by Equation (34) [308]):

8HClO_3_ → 4HClO_4_ + 2H_2_O +3O_2_ + 2Cl_2_ (34)

Table 15 and Table 16 show expected product constituents for optimized and non-optimized chlorine dioxide generators, respectively; it is not clear from the literature whether all possible side-reaction or decomposition compounds were considered (adapted from [308]). It is interesting to note that the product from the optimized, chlorite-based generation system contains very little chlorite, but chlorate may be present in an amount proportional to the free chlorine present. The optimized chlorate-based generation system exhibited significant concentrations of chlorate, sulfuric acid, hydrogen peroxide, and perchlorate ion. Product concentrations of these compounds was even greater in the non-optimized operation. As expected, the non-optimized, chemically based generation system exhibited higher product concentrations of byproducts than did the optimized systems. The electrochemical system yielded lower concentrations of by-products and higher product purity than the chemically based processes. Specific solution purity (not including side reactions or decomposition compounds) for a commercially generated chlorine dioxide solution using chlorate (Equation (28)) is given in Table 17 [311]. One can conclude from these data that even optimized generation systems can yield a host of species, some of which are themselves oxidizers, in addition to chlorine dioxide. Ergo, even if chlorine dioxide itself is shown to be compatible with polyamide membranes, some of the byproducts are not.

#### 5.2.2. Efficacy

Disinfection efficacy of chlorine dioxide depends on pH, temperature, turbidity, concentrations of other oxidizable species, and the presence of nitrogen and hardness. The effect of pH on inactivation time is in uncertain. While chlorine dioxide has greater disinfection efficacy than chlorine over the pH range of pH, 5–10 [312], some studies have indicated that pH does affect its disinfection efficacy, with alkaline pH showing significantly shorter contact times for deactivation of viruses than near neutral pH (note that all contact times are short, even for low pH conditions) [313,314,315,316]. Conversely, studies by Clarke et al. [317] with *Cryptosporidium* showed no effect of pH on inactivation time. Junli et al. [318] studied *E. coli*, *Staphylococcus aureus*, *Chloropseudomonas*, *Bacillus subtilis*, and *Sarcina*; conclusions were that these bacteria are effectively killed within the pH range of 3.0–8.0, but that some species may have variable resistances to chlorine dioxide.

Temperature and turbidity also effect the contact time needed for inactivation of microbes. Colder temperatures increase the contact time needed; the USEPA, in development of the Surface Water Treatment Rule, assumed that for every 10 °C decrease in temperature, the contact time required for inactivation doubles [178]. Turbidity (particulates) and amassed microorganisms can shield target microorganisms from exposure to the disinfectant [319]. Turbidity as low at 3 NTU can negatively affect performance [319].

The concentration of other oxidizable species, such as organic matter and divalent metal ions, as well as nitrogen, and hardness affect the disinfection efficacy of chlorine dioxide [319,320]. Chlorine dioxide readily oxidizes organic matter (yielding chlorite), to the degree that chlorine dioxide loses residual to disinfect microbes [319]. Work by Copes et al. [320] demonstrated that the largest demand on chlorine dioxide, however, comes from divalent metal ions in solution, including iron and manganese. The interaction of nitrogen and hardness in water also lead to an increase in oxidant demand [320,321]. In order of decreasing demand, Copes et al. [320] found the following effect: concentration of divalent metals ions >> pH > concentration of nitrogen and hardness in solution.

#### 5.2.3. Determination of Chlorine Dioxide in Water

There are several methods to test for chlorine dioxide [322]. Kortelyesi [322] offers a complete review of all methods, the most common of which are summarized here. It is noted that these methods, particularly the DPD method used almost exclusively by field personnel for chlorine dioxide analysis, are inaccurate and depend on the skill of the analyzer. There is no field test to accurately and precisely determine the concentration of chlorine dioxide.

##### Iodometric Titration (Standard Method 4500-ClO2 Method B)

This method involves oxidation of iodide ion to iodine by chlorine dioxide, followed by titration with a standard sodium thiosulfate solution. This method can also be used for other oxidizers containing chlorine. This is a good method to use if chlorine dioxide is the only chlorine-based oxidizer present (due to interference by other chlorine species). This is the preferred method to develop calibrating solutions for other chlorine dioxide measurement methods, such as spectrophotometric [322]. However, this method does not easily distinguish among chlorine dioxide, chlorine, chlorite and hypochlorite [323], and, thus, is not recommended for determination of chlorine dioxide other than for preparation of stock solutions.

##### Spectrophotometric (USEPA Method 327.0)

The USEPA Method 327.0 for determination of chlorine dioxide concentration relies on the Horseradish Peroxidase (HRP)/Lissamine Green B (LGB)/reagent. HRP catalyzes chlorite conversion to chlorine dioxide, which then oxidizes LGB. Oxidized LGB has a reduced absorption in the red region of the visible spectrum; the reduction in absorption is proportional to the concentration of chlorine dioxide. This method cautions that due to the reactive and volatile nature of chlorine dioxide, detection limits, accuracy, and precision are analyst technique and instrument dependent [324]. Hence, this method is not recommended for field analysis, as different operators are routinely used for this sort of testing in industrial settings.

##### Colorimetric

Colorimetric methods are based on the reaction of chlorine dioxide and a dye; the reaction decreases the absorbance of the dye. The major limitation of these methods is that the purity of the dyes can vary from 40% to 95% [322]. The impurities also react with chlorine dioxide, reducing the amount available to react with the dye, which directly affects the decrease in absorbance of the dye [322]. Impurities can also affect the stability of the dye [238]. The DPD dye is well suited for measuring chlorine in the field not for measuring chlorine dioxide [322]. The DPD method (Standard Methods 4500-ClO2 Method D-Reserved [325]) is based on a differential determination of the various oxidizing species in the sample, so the potential for interference in the determination of chlorine dioxide is probable [323]. Other species, such as chromate and oxidized manganese, interfere with the DPD method [322]. The potential for inaccurate results has led to the DPD method in *Standard Methods for the Examination of Water and Wastewater* to be placed in reserve (not recommended for use) [325].

#### 5.2.4. Dosing

Compared to chlorine and chloramines, much lower concentrations of chlorine dioxide are required for disinfection. Studies have shown that 5 min of exposure to as little as 0.1 ppm of chlorine dioxide can successfully disinfect against *Salmonella paratyphi B, Eberthella typhosa*, and *Shigells dysenterias* (common pathogens) [326]. Milpas [327] showed chlorine dioxide to be at least as effective as chlorine against *E. coli*, *Salmonella typhosa*, and *Salmonella paratyhi*.

The ideal feed point for chlorine dioxide is post clarification and filtration, where the oxidant demand of organics and soluble transition metals is lower [299], and dosages of the product are, therefore, lower (low dosage of product is also necessary to minimize the concentration of carry-through reactants and chlorine dioxide decay products, including chlorine, chlorite, and chlorate.) However, feeding chlorine dioxide post clarification/filtration is not optimal for disinfection of membrane-based desalination pretreatment systems, as microbes can proliferate within clarifiers and filters, increasing oxidant demand and raising the risk for some bacteria to pass through the disinfection zone unaffected.

One of the most extensive studies on chlorine dioxide efficacy for polyamide-membrane biofouling control was conducted by Eriksson and Dimotsis [328]. The use of chlorine dioxide at three industrial plants was studied with inconsistent results, both in terms of biofouling control efficacy and in membrane degradation. The impediment to long-term biofouling efficacy data in most studies was the degradation of the membranes due to oxidation [328,329].

#### 5.2.5. Advantages and Limitations

Major advantages of chlorine dioxide are that it minimizes the formation of THMs and HAAs and it remains a gas in water, allowing for penetration into biofilm for more effective microbial kill than with chlorine. The gas also passes through the polyamide membrane un-rejected, to provide disinfection of the permeate stream. Further, studies have shown that relatively low dosages, on the order of less than 0.5 ppm, are required to achieve effective disinfection in membrane systems [328,330].

While advantages of chlorine dioxide are relatively easy to define, limitations are more challenging to describe. A few of the simple limitations involving the nature of the chemical and its byproducts are discussed here. Discussion of more complex issues follows.

Chlorine dioxide is a free radical in water [299], and in concentrated solution, is highly volatile and unstable. Gaseous chlorine dioxide and aqueous solutions of greater than 4% will detonate when compressed [299]. Hence, chlorine dioxide must be generated on site, which requires equipment, real estate, and trained personnel to handle the generation system. The difficulty in feeding the product to a therapeutic dose when other oxidant-demanding species, such as organics and divalent metals (e.g., iron and manganese), are present [330] is another concern. Further, the inaccuracies of the most common analytical methods introduced by operator technique or competing species make it difficult to quantify the concentration of chlorine dioxide present.

Chlorine dioxide does form some DBPs including THMs. In seawater treatment, tribromomethane (bromoform, CHBr_3_) form due to the presence of bromide ions. Al-Otoum et al. [331] reported a maximum total THM concentration of 0.077 ppm and an average value of 0.005 ppm in a study of 294 water samples from plants using chlorine dioxide in Qatar; the primary THM found was bromoform. A maximum contaminant level goal (MCLG) of 0 ppm for bromoform, and a MCLG of 0.06 ppm for total THMs has been established by the United States Environmental Protection Agency (USEPA) [332].

Other DBPs formed by chlorine dioxide include chlorite and chlorate [333]. The presence or formation of chlorite and chlorate, and their potential health impact is a significant limitation [299,334]. About 60% of chlorine dioxide converts to chlorite in water [299]. There is also carry through of chlorite and chlorate reactants into treated water. The USEPA has established a MCLG for chlorite of 0.8 ppm [158,331] and a health reference level (HRL) of 0.21 ppm [158,335]; therefore, 1.3 ppm is the theoretical maximum chlorine dioxide that can be applied, unless a chlorite removal process is employed [299,336]. Additionally, due to adverse effects of chlorine dioxide in laboratory animals (e.g., hemolytic anemia [301]), the maximum residual disinfection limit (MRDL) of chlorine dioxide leaving a drinking water facility is 0.8 ppm [332,337]. (The United Kingdom water quality regulations [338] limit combined concentrations of chlorine dioxide, chlorite, and chlorate to 0.5 ppm, thereby limiting the feed dose of chlorine dioxide to 0.75 ppm.) The Occupational Health and Safety Agency (OSHA) has set a permissible exposure limit (PEL) of 0.1 ppm chlorine dioxide gas at 25 °C and 1 atmosphere [339] While the USEPA has not yet established a MCLG for chlorate, it is a probable health concern [334,336]. The USEPA has established an HRL of 0.210 ppm for long-term exposure [340], and it is anticipated that short-term exposure HRLs will also be developed in the future [335]. In a report issued in 2002, the California Office of Environmental Health Hazard Assessment [338] recommended a chlorate action level of 0.2 ppm, but as of 2015, the notification level remained at 0.8 ppm [341].

As a result of the low dosages/residual limitations the low dosage limit of chlorine dioxide may not be high enough to meet the disinfection demand in industrial systems. For surface water or wastewater make-up sources, the competing demand by organics may require more chlorine dioxide than the limits allow. Similarly, for ground water make-up sources, demand due to metals in reduced states also compete with bacteria for the biocide.

The objectives of using chlorine dioxide are to disinfect the pretreatment system as well as consideration of disinfection of the polyamide membrane itself, due to the ability of the gas to penetrate biofilm and the results of some research indicating some compatibility of the oxidant with polyamide membranes. Perhaps the primary limitation to using chlorine dioxide for these purposes is the conflicting information regarding the compatibility of chlorine dioxide and polyamide membranes found in the literature [328,342,343]. This is an opportunity for definitive research, as the disinfection capability of chlorine dioxide is superior to chlorine, particularly with respect to kinetics (Table 4) and its ability to penetrate biofilm. A defined set of feed water conditions under which chlorine dioxide will not degrade polyamide membranes needs to be clarified. If direct contact with polyamide membranes is possible, without degradation to the membrane polymer or formation of hypobromite in seawater, chlorine dioxide may be an exceptional tool for membrane-targeted biofouling mitigation and perhaps even prevention.

Current status of research indicates that despite lack of free chlorine in the recipes shown in Equations (25)–(31), chlorine dioxide does not appear to be completely compatible with RO membranes. Adams [342] conducted the definitive study on interaction of chlorine dioxide and polyamide membranes in 1990. Results of this work and research by Glater et al. [343], demonstrated loses in membrane rejection was a function of pH. Membrane rejection decreased at any pH, with higher pH exposure resulting in greater loss of slat rejection. It is unclear if the membrane damage is due to chlorine dioxide itself and/or the presence of chlorite and chlorate, which each have significant oxidative potentials [344].

**Figure 21:**
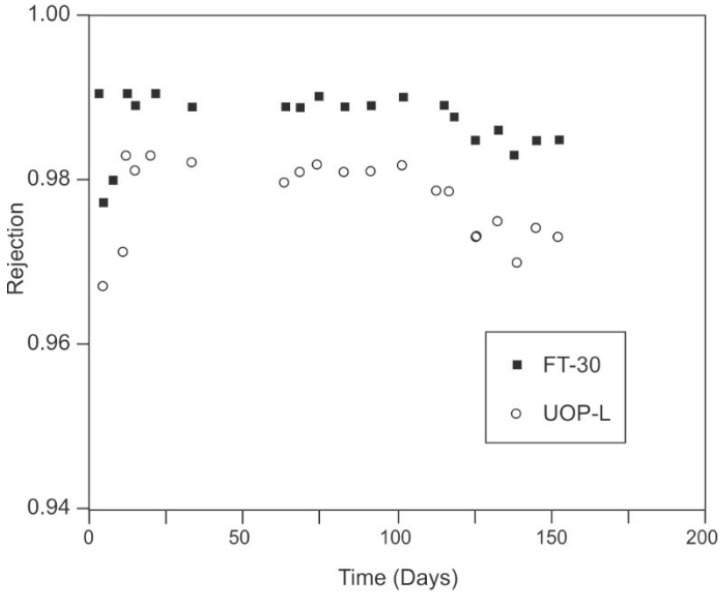
Rejection (reported as decimal) vs. time for DuPont-FilmTec FT-30 and Universal Oil Products UOP-L membranes. Conditions: 22 bar, pH 7.0, conductivity 6000 µS/cm with 1 ppm chlorine dioxide [342]. Reprinted with permission of Elsevier B.V.

**Figure 22:**
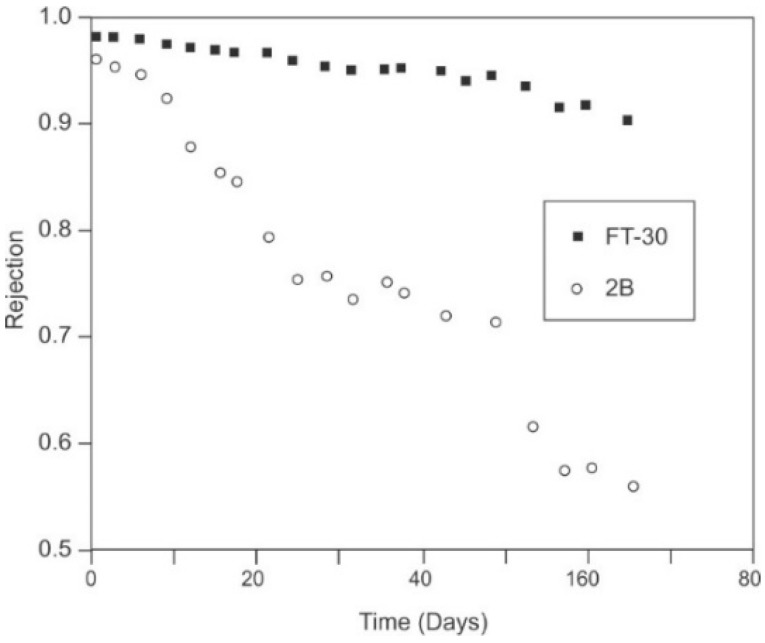
Rejection (reported as decimal) vs. time for DuPont-FilmTec FT-30 and Desalination Systems 2B membranes. Conditions: 22 bar, pH 7.0, conductivity 6000 µS/cm with 5 ppm chlorine dioxide [342]. Reprinted with permission of Elsevier B.V.

Representative results as reported by Adams [342] include a loss of rejection for the DuPont-FilmTec FT-30 polyamide membrane from 99% to 98% over 152 days at a exposure of 1 ppm (152 ppm-hours), as shown in Figure 21 [342], and a decrease from 99% to 96% in 24 days at an exposure of 5 ppm (120 ppm-hours) shown in Figure 22 [342], for membranes operating at pH 7. To investigate the effects of pH, Glater et al. [343] tested membranes at pH 8.6 that showed severe damage at 1200 ppm-hours; membranes tested at pH 5.8 exhibited less damage given the same exposure. (Recall that chlorine exposure to polyamide membranes guidelines notes that approximately 1000 ppm-hours of exposure results in damage to the membrane.)

Studies by Alayemeike and Lee [213] and Eriksson and Dimotsis [328] also confirm the effect of pH on degree of membrane degradation caused by chlorine dioxide. Table 18 summarizes results of research conducted by Alayemeike and Lee [213] regarding the effect of pH on seawater polyamide membrane performance when exposed to 100 ppm–hours of chlorine dioxide generated without chlorine reactants. As the data show, alkaline pH resulted in the worst damage to the membrane tested, while acidic pH had minor effects; neutral pH resulted in an increase permeate flux with little loss in rejection. The explanation for this result at neutral pH has to do with the effect of pH itself on the membrane combined with the effects of the oxidant [214]. Alkaline pH results in a more negatively-charged membrane which has two direct effects on the membrane [214]: the polymer chains in the 3-dimentiaon polyamide surface start to repel each other, tending to open up the polymer matrix resulting in an increase in flux, and charged molecules tend to be rejected to a higher degree. The observed *lower* salt rejection and much higher flux at pH 9, as reported in Table 18, is presumably due to membrane damage by chlorine dioxide [214].

Eriksson and Dimotsis [328] in their study considered two power plants and one paper mill using pure chlorine dioxide generated by electrochemical and catalytic processes to avoid any membrane degradation with chlorine byproduct. Results demonstrated little or no membrane degradation while operating at a feed pH of 6.2, but severe degradation in a matter of weeks and days for the plants operating at feed pH values of 8.2 and 9.0, respectively. They drew two major conclusions. First, pH plays a role in degradation; pH greater than 8 results in damage to the membrane. The presence of iron and manganese can catalyze the reaction of chlorine dioxide with the membrane at any pH. The disinfection strength of chlorine dioxide is enhanced at higher pH for species including *Giardia* and viruses [345] and thus, may be more likely to oxidize the polyamide membrane at higher pH as well. And, second, due to conflicting data in the literature [253,342,343,346] and the results of testing in their work [328], chlorine dioxide is not recommended for use in membrane desalination systems until the mechanisms of chlorine dioxide/membrane interaction/degradation are better understood.

Other conflicting studies involve chlorine dioxide used with polyamide membranes for seawater desalination. Sandin et al. [347] and Kwon et al. [348] point to possible chlorine dioxide-based oxidation of bromide found in seawater to hypobromite, which subsequently leads to polyamide bromination and degradation. On the other hand, a review by Mizuta [349] indicated that most researchers found that chlorine dioxide does not oxidize bromide [350,351,352]; Agus [350] suggested that further investigation should be conducted in this area for confirmation.

These conclusions agree with the opinion of several membrane manufacturers, including Hydranautics [330], DuPont [353], Toray [354], and Microdyn Nadir [355], regarding the effect of chlorine dioxide on polyamide membranes. These manufacturers recommend against using chlorine dioxide for polyamide membrane pretreatment or cleaning until further investigation is conducted. Hydranautics states (reprinted with permission from Hydranautics [330]):

“At this point further studies are needed to more fully characterize the effect on membrane performance. In particular, Hydranautics is concerned with the effect of transition metals which are known to greatly accelerate the membrane oxidation for chlorine and chloramine attack. Since the reaction of ClO_2_ is different than OCl^−^, the interaction of ClO_2_ with the membrane is not yet fully understood. Hydranautics does not fully endorse the use of ClO_2_ for frequent cleaning or daily dosing until more extensive studies are done, especially with the presence of transition metals.”

Chlorine dioxide is an effective biocide due to its ability to penetrate biofilm, which would be a great benefit for direct membrane disinfection. But, clearly, more research work with real-world applications is necessary to definitively determine the impact of chlorine dioxide and its DBPs, chlorate and chlorite, on polyamide membranes. Therefore, the current recommendation is to avoid the use of or remove chlorine dioxide, chlorite, and chlorate from membrane feed water to prevent potential oxidation of the polyamide layer [353].

#### 5.2.6. Removal of Chlorine Dioxide and its DBPs, Chlorite and Chlorate

Sodium bisulfite, commonly used for dechlorination and dechloramination [356], is not recommended for removal of chlorine dioxide. Transition metals catalyze the oxidation of the sulfur (IV) compounds, including bisulfite [357]. Bisulfite, when oxidized, yields persulfate or peroxodisulfate anions. These compounds react with the byproduct chlorite to reproduce chlorine dioxide [353]. When this occurs within the concentration polarization boundary layer of the membrane, it results in significant oxidation of the polyamide membrane [353].

The preferred methods for neutralizing chlorine dioxide employ sodium thiosulfate or sodium sulfite [358], respectively:

5Na2S_2_O_3_ + 8ClO_2_ + 9H_2_O → 10Na2HSO_4_ + 8HCl (35)

5Na2SO_3_ + 2ClO_2_ + H_2_O → 5Na2SO_4_ + 2HCl (36)

Theoretically, 1 ppm of thiosulfate is required per 1 ppm of chlorine dioxide, and 2.95 ppm of sulfite is required per 1 ppm of chlorine dioxide.

Photodecomposition of chlorine dioxide with UV light has also been shown to be an effective removal method [359] for direct destruction of chlorine dioxide. Chlorine dioxide photodecomposition reactions proposed by Karpel Vel Leitner et al. [360], Zika et al. [361], and Bowen and Cheung [362] are as follows, respectively:

10ClO_2_ + 5H_2_O → 4Cl^−^ + 6ClO_3_^−^ + 3.5O_2_ + 10H^+^ at 253.7 nm (37)

4ClO_2_ + 2H_2_O → 2ClO_3_^−^ + ClO^−^ + Cl^−^ + O_3_ + 4H^+^ at 300–436 nm (38)

2ClO_2_ + H_2_O → ClO_3_^−^ + Cl^−^ + O_2_ + 2H^+^ at 300–436 nm (39)

All of these decomposition reactions yield chlorate [360,361,362]. Chlorate is a strong oxidizer in acidic conditions (reduction potential of +1.47 volts) and a moderate oxidizer in alkaline conditions (reduction potential of +0.63 volts) [344] that must be removed prior to polyamide membranes (see discussion that follows).

Other removal methods for chlorine dioxide include carbon, membrane processes and ion exchange. Sorption on activated carbon is simple, but this is reversible, and desorption occurs rapidly [363]. Membrane biofilm reactors under anaerobic conditions [364], are an efficient method, but not practical for make-up water sources to membrane-based desalination systems except when wastewater is the feed source. And, anion exchange resins have been tried, but competition from other anions in solution will limit the efficacy of this technique for chlorine dioxide removal [365].

Much of the chlorine dioxide literature discusses removal of chlorite as a DBP of chlorine dioxide decomposition rather than outright removal of chlorine dioxide [302,345,363,366,367]. Chlorite is the dominant degradation by-product of chlorine dioxide reactions [363,367,368]. Yang [310] reported that the reaction and decay of chlorine dioxide in water yields both chlorite and chlorate, with approximately 60% of the applied chlorine dioxide forming chlorite and 8% forming chlorate; this concurs with work by Al-Otoum et al. [306]. For polyamide membranes, chlorite ion is important as it can react with excess acid carried over from the initial production of the chlorine dioxide solution to reproduce chlorine dioxide and free chlorine that will oxidize the membrane [369]. Chlorite itself is also a strong oxidizer, with an oxidation-reduction potential of 1.64 volts in acidic conditions and 0.78 volts in alkaline conditions [344].

Results of experiments conducted by Donaque, et al. [369] to investigate the behavior of chlorine dioxide in seawater are presented in Table 19 The data show that chlorine dioxide generated using chlorine and chlorite (via Equation (23)) yielded free chlorine (from excess reactant), that dissipated after 30 min; chlorine dioxide, two-thirds of which dissipated after 30 min; and chlorite, that *increased* in concentration after 30 min. The increase in chlorite concentration is indicative of chlorine dioxide decay reactions yielding chlorite. Impurities in the product, such as peroxide used in chlorate-based generation systems, react with chlorine dioxide to also form chlorite [308]. In contrast, Gordon, et al. [359] found that the presence of chlorite was due to carry through of reactant rather than decay of chlorine dioxide. In either case, chlorite is a contaminant of concern for polyamide membranes, whether it carries through into the product of chlorine dioxide generation or due to decomposition of chlorine dioxide during use.

Chlorite removal can be achieved using one of two methods, neither of which is ideal for pretreatment prior to membrane desalination systems. The use of reducing salts, such as ferrous chloride or ferrous sulfate in reactions that are instantaneous at pH 5–7 can be used to reduce chlorite. This technique is not recommended prior to a membrane system, due to the generation of iron oxide (a membrane foulant) via oxidation of the iron (II) by the chlorite [345,366]. The other method involves filtration through activated carbon [345,367]. Gounce and Voudrias [363] established that an oxidation-reduction reaction is the mechanism by which chlorite is removed from water via carbon filtration. The degree to which chlorite is chemically reduced was found to be dependent on the concentration of NOM, pH, hydraulic loading, carbon media particle size, and temperature [363]. Reduction is enhanced at low NOM concentration, low pH and hydraulic loading, small carbon particle size, and high temperature. Despite the ability of carbon to remove chlorite, Schajnoha [345] determined that if any free chlorine is present, the reaction of chlorite with carbon and free chlorine can yield chlorate; Gounce and Voudrias [363] found that chlorate is indeed formed in the when free chlorine is present, but it is not chemically reduced by carbon; chlorate that is present is physically and *reversibly* adsorbed onto the carbon media. Some suggest RO is capable of efficient removal of chlorate ion [365], but oxidization of the polyamide membrane is a great risk, as described previously.

Removal of chlorine dioxide is complex, but given the doubt about its effect on polyamide membrane integrity, its use requires removal prior to the membranes. Moreover, chlorite and chlorate, present via carry through of reactants, decomposition of chlorine dioxide, or formation via carbon reduction, must also be removal prior to the membranes. Removal of these byproducts is also problematic.

### 5.3. 1-Bromo-3-Chloro-5,5-Dimethylhydantoin (BCDMH)

BCDMH in water yields hypochlorous acid and hypobromous acid [370]. Reactions involving these acids with bromides (typically found in seawater) form additional hypobromous acid. Hypobromous acid will attack and degrade polyamide membranes [360] just as hypochlorous acid does.

**Figure 23:**
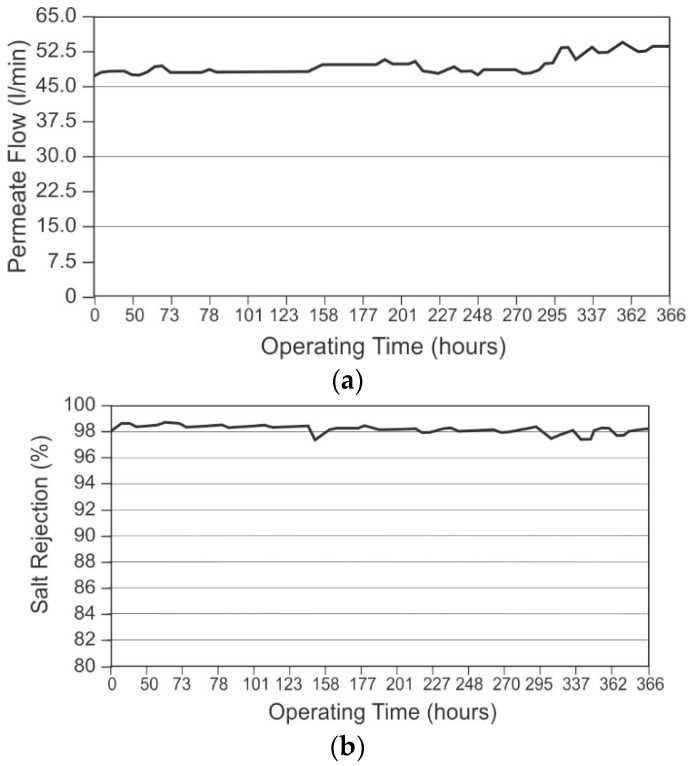
Normalized permeate flow (**a**) and normalized salt rejection (**b**) as a function of operating time for a polyamide seawater membrane operating on BCDMH biocide dosing. Conditions: feed water: Atlantic Ocean at St. Croix, United States Virgin Islands; feed pressure: 57 bar; system recovery: 43%; membrane: Koch Membrane Systems 2822SS; BCDMH dosing: 4-h intervals (dosage concentration was not provided) Obtained from patent application US 20060032823 A1 [371].

However, a patent application by Harrison and Sisk [371] considers the use of BCDMH for direct, on-membrane disinfection of polyamide membranes and they claim little or no membrane damage. They propose that halogen in combined form releases the halogen slowly over time to sufficiently disinfect the membrane but not damage it. Example 3 in the patent application describes a seawater field test with intermittent dosing of BCDMH. Over the 366 h-test (15 days), the salt rejection was fairly stable with some fluctuation near the end of the test (ppm-hours exposure could not be determined, as concentration of BCDMH was not provided; however, the invention claims halogen concentration of 0.05–1 ppm with 0.5 to 1 ppm being preferred, leading to an exposure of 183 to 366 ppm-hours). The normalized permeate flow increased by 12% during the relatively short test (see Figure 23). 

The increase in flux shows damage to the membrane, contrary to the conclusions by the Harrison and Sisk [371], who claim a more significant increase in normalized permeate flow would be necessary to indicate membrane damage (note that no virgin or chlorine-exposed control membranes were tested for comparison in the example cited here). The salt rejection is fairly stable for the first 158 h of the test, but then decreases slightly, and then fluctuates near the end of the test. These salt-rejection data are inconclusive. The patent application does include data with polyamide fibers, comparing tensile strength, elongation, and Young’s Modulus (a measure of the stiffness of a solid material) for fibers exposed to BCDMH, bromine, and chlorine; all membrane fibers showed loss of integrity, but the BCDMH membranes showed the least damage, particularly with respect to Young’s Modulus. The patent application does not present any data relevant to biofouling control.

Longer-term tests at known dosages would be required to determine if BCDMH could be an alternative to chlorine for direct us on polyamide membrane for biofouling control. An economic study would also be required.

### 5.4. Dichloroisocyanurate (DCC)

DCC, a compound related to chloramine compound, has shown promising results for disinfection efficacy with minimal membrane damage, even with direct membrane exposure [88,372,373,374]. DCC releases hypochlorous acid and isocyanuric acid, toxic to microorganisms [26,375]. Bench-scale tests by Yu et al. [374] showed that flux and salt rejection were maintained after exposure to DCC, intermittently dosed as 5000 ppm total available chlorine for up to 3 h when run on 2000 ppm NaCl solution for 120 h. The antimicrobial effect of DCC as compared to chlorine on *Pseudomonas aeruginosa* was also determined in the study. DCC yielded a 2.0 log concentration reduction, while chlorine yielded a 1.6-log reduction [375].

There is currently no regulatory approval for using DCC as a biocide, and it is more expensive than chlorine [26]. However, due to the promising result in terms of biocontrol and limited membrane damage, additional research is recommended for pilot- or larger-scale basis as well as on mixtures of bacteria that are more representative of actual membrane system populations.

### 5.5. Nitric Oxide (NO) Donor Compounds

NO is toxic to bacteria. Cell death occurs via damage to the intercellular DNA and destruction of the iron-sulfur centers, which yields ionic iron and iron-nitrosyl compounds [26]. This results in dispersion of the biofilm in favor of the planktonic state of bacteria [376,377]. NO donor compounds, with [6-(2-hydroxy-1-methyl-2-nitrosohydrazino)-N-methyl-α-hexanamine] (MAHMA NONOate) as the compound of choice, have recently been shown to effectively remove EPS and kill bacteria in wastewater RO membrane applications [378,379]. This technique is still in the research and development stage. Some populations can develop resistance to NO donors [374], and no regulatory approval has been given for use of NO donors as biocides [26]. However, the positive results of work by Barnes et al. [378] indicate that more research should be conducted into the use of NO donors for biocontrol of polyamide membranes.

### 5.6. Advanced Oxidation Processes (AOPs)

AOPs are used to generate highly reactive radicals, such as the hydroxyl radical [380]. This radical has been traditionally used for the remediation of organic and inorganic contaminants in wastewater [376,381] and address removal of taste and odor compounds, volatile organics, and pesticides, and not for disinfection [380]. The most common AOPs use the ozone plus UV, ozone plus peroxide, or peroxide plus UV. Hydroxyl radicals are formed via the decomposition of ozone (as described previously). The goal is to generate more of the hydroxyl radical and force more reactions with more hydroxyl and other free radicals [77,380]. However, hydroxyl is not a recognized biocide presumably due to the low the concentration of resultant hydroxyl radicals and the fact that no research data is available on the disinfection potency of hydroxyl radical [380]. Ergo, the companion oxidant, such as ozone, is to provide any disinfection credits for potable or food and beverage applications.

A literature search found few AOPs currently used for disinfection of membrane pretreatment systems on an industrial or municipal scale. AOPs have been proposed to replace chloramination to treat RO permeate for reclaim of tertiary effluent that will be reused as potable water in Los Angeles, California [382]. AOP using ozone and UV is currently used on secondary effluent reclaim water as make-up to a polyamide RO membrane at the University Area Joint Authority (State College, PA, USA) [383]. AOPs are generally more effective on higher quality permeate water than make-up water [384].

**Figure 24:**
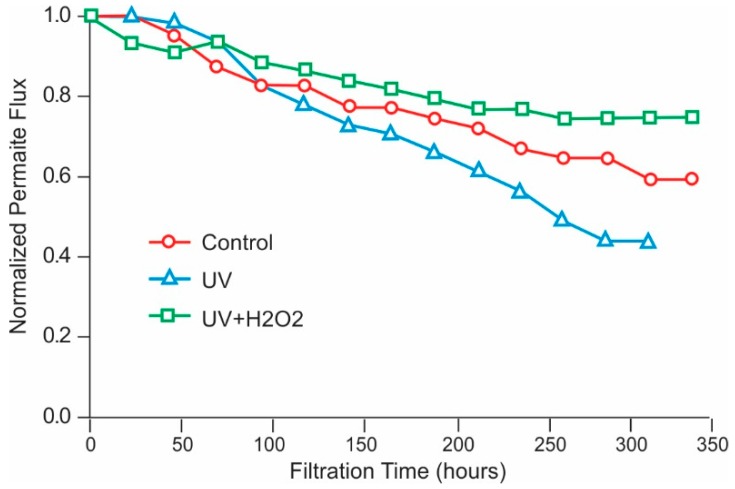
Normalized permeate flux as a function of operating time as a measure of biofouling control for membrane pretreated with MP-UV/hydrogen peroxide, UV only, and no bio-control method. [385]. Reproduced by permission of The Royal Society of Chemistry.

Lakretz, et al. [385] investigated using an AOP using MP-UV with peroxide for biofouling control of brackish water RO processes. In this lab- and bench-scale study, brackish water from Mashabei Sade (south Israel) was treated with 2.5 ppm peroxide, followed by UV at a dose 137 mJ/cm^2^ and 99% UVT_254_ (excess peroxide was removed prior to the membranes using sodium thiosulfate). Results demonstrated that the polyamide membranes exposed to the AOP-treated water had a slower rate of normalized flux decline that membrane treated with UV alone or the control (see Figure 24). Based on this study this particular AOP shows promise for membrane biofouling control in larger-scale applications; significant pilot work is required prior to full-scale implementation.

### 5.7. Extrapolative Cleaning

Based on work by Bereschenko et al. [28] which found that a mature biofilm structure can form in a little as one month on a polyamide membrane, implies that it may be advantageous to minimize the deleterious effects of biofouling on membrane performance by intervening early. Preventative cleaning to minimize bacterial adhesion or to attack the EPS in its formation stage, may be successful in for low- to medium-risk systems. It is easier to remove singularly attached bacteria than mature biofilm [50]. Chesters, et al. [243] advocate preventive cleaning before “observed” fouling manifests in performance decline. They recommend performing autopsies on relatively new membrane elements to determine when the membranes will begin to show signs of fouling, which typically occurs before a decline in performance is perceived. This becomes the point in time when the membranes should be cleaned. At the very least, they suggest cleaning as soon as fouling is observed via deterioration in normalized performance. Cleaning at this point in the development of membrane fouling can reduce the overall frequency of cleaning over the lifetime of the membrane, and, therefore, minimize the damage to the membrane that occurs during chemical cleaning [246]. More work is necessary to confirm that preventative cleaning of this sort does indeed minimize the effects of biofouling on performance and membrane life.

## 6. Discussion

Biofouling of polyamide membranes is ubiquitous and is a serious problem for most desalination systems. Membrane biofouling results in a decline in performance with losses in productivity and salt rejection, which can lead to shorter useful membrane life. Biofouling necessitates frequent membrane cleanings, which also lead to shorter membrane life. Based on the work described herein, most currently employed control methods focus on pretreatment of feed water or cleaning after-the-fact. Limitations of these techniques is that they do not directly addressing biofouling prevention and biofouling control on the polyamide membrane itself. The reason for this is that most effective disinfection techniques currently used involve oxidizers, which can damage the membrane on direct contact.

Oxidizing biocides are often the most effective options for minimizing new biogrowth or attacking established biofouling and can keep the membrane pretreatment system somewhat free of microorganisms. Chlorine is the most common oxidizing biocide currently used for this purpose. It is effective and easy to employ. However, chlorine and other oxidizers cannot be used directly on polyamide membranes, and, therefore, must be removed prior to the membrane system. This leaves the system downstream of removal, including the membranes themselves, vulnerable to regrowth of bacteria. Additionally, some oxidizers lead to the formation of DPBs or other undesirable products. Therefore, numerous municipalities which provide feed water to many membrane systems, are moving away from chlorine to other oxidizers such as chloramine or ozone. While chloramine is less aggressive than chlorine, there is conflicting information as to the potential for damage to polyamide membranes with direct contact. Hence, membrane manufacturers recommend removal of chloramine prior to the membranes. Ozone is very aggressive to membranes and must also be removed. The use of ozone adds to the capital cost of the water treatment system, so it is seldom used at the industrial level; industrial plants encounter ozone generally through municipal feed water sources.

Some non-oxidizing biocides (e.g., DBNPA and isothiazolones) can be used directly on polyamide membranes to control biofouling as they are not as aggressive to the membrane. Because they are less aggressive, they are most effective at keeping membranes clean when the membrane surfaces are new and/or freshly cleaned of biocontamination than for primary disinfection. They are not effective for pretreatment (oxidizers are preferred) or for primary cleaning of membranes already fouled with microbes. Thus, they are typically used in conjunction with another disinfection technique such as oxidizers. DBNPA is optimally applied in slug doses while isothiazolones are used for long-term soak. Also, DBNPA must be used off-line for food and beverage applications. Other non-oxidizing biocides, such as quats and aldehydes are not recommended as membrane exposure will negatively affect performance.

Physical and photochemical techniques, such as UV, can be very effective at disinfection. However, unlike chemical disinfection, these techniques lack residual. Species not neutralized during disinfection, are free to infect downstream equipment, piping, and membranes. Further, there is the potential for photoreactivation and dark repair of treated organisms. UV equipment and maintenance adds capital and operating costs to the plant that is avoided when using chemical biocides.

Cleaning is usually conducted after biofouling of the membranes has already resulted in performance decline. At this point in the life cycle of biofouling, aggressive cleaning chemical and physical techniques are required. However, polyamide membranes are sensitive to aggressive chemical techniques. And, the nature of a spiral wound element does not lend itself to truly effective physical cleaning of the membranes. The DO-HS approach has been used as a preventative cleaning technique, but is not widely practiced, presumably due to the supplementary equipment, capital costs, and complexity that is added to the system.

Table 20 summarizes the advantages and limitations of the most commonly used membrane biofouling control techniques discussed herein. Chemical, physical and cleaning techniques currently employed are not completely effective at preventing biofouling of polyamide membranes. They are only capable of controlling biofouling to varying degrees; none address direct membrane disinfection. And, some damage polyamide membrane on contact, while others have undesirable side effects such as producing DBPs and actually promoting biogrowth by increasing AOC or via photoreactivation/dark repair. There are some promising techniques in various stages of research that may further minimize or even prevent biofouling of polyamide membranes. These techniques include membrane modifications, specific disinfection chemicals, and proactive membrane cleaning.

Coating of a polyamide membrane with the antimicrobial agent TOB has shown initial success for biocontrol, as shown in Table 12. Research efforts yielded coated membranes with slightly improved flux and rejection, and with improved biofouling resistance. In general, however, surface coatings are plagued with issues regarding mechanical and chemical stability of the coating. Additional work with TOB and other anti-microbial agents should be conducted to advance the long-term performance and stability the resultant membranes.

Membrane modification using nanoparticles has shown preliminary success at improving the resistance of the polyamide membrane to adhesion and subsequent biofouling. These modifications alter the surface charge or hydrophilicity of the membrane. However, as described in this paper, some modification can lead to unintended effects, such as increasing the potential for fouling with other, non-biological species. Work should continue to find the appropriate mix of polymer/nanoparticle to minimize all types of membrane fouling.

Research efforts have demonstrated marginal performance of GO membranes compared to TFN and traditional polyamide membranes, as described in Table 13. However, GO membranes have shown improved resistance to fouling with microbes (see Figure 19) and to degradation upon exposure to chlorine (see Figure 20). More work is necessary to identify the most promising membrane preparation techniques that can result in high-performance membranes with resistance to biofouling and chlorine. Focus should also include scalability of the formation process, and pilot testing of the resultant membranes.

Chlorine dioxide, NO donor compounds, and DCC, have each demonstrated good efficacy for microbial control albeit some debatable compatibility with polyamide membranes. NO donor compounds and DCC have demonstrated good compatibility in lab-scale; pilot- and full-scale performance and compatibility research should be conducted. Chlorine dioxide, an oxidizing biocide, is commercially available and has received much attention for use with membrane systems due to its excellent disinfection capability. However, questions remain about compatibility with polyamide membranes. Some work has shown resistance of polyamide membrane to chlorine dioxide generated using non-chlorine gas or hypochlorous acid reactants, but other research contradicts this (Figure 21 and Figure 22 clearly show degradation upon exposure to chlorine dioxide). Studies using seawater systems have also shown conflicting results with respect to hypobromite formation and subsequent bromination/degradation of the polyamide membrane. A comprehensive study of compatibility is necessary to determine if indeed chlorine dioxide can be used to minimize biofouling without leading to the degradation of the polyamide membrane either directly or via by-products such as chlorite, chlorate, or bromine compounds.

AOPs, commonly used for wastewater effluent treatment, have been used as RO pretreatment for a handful of wastewater reuse projects. Figure 24 shows the results of one study using MP-UF and peroxide improved biocontrol over UV only and the control with no pretreatment. While, application for RO pretreatment is early in development, the potential is good for AOPs in wastewater reuse systems.

Proactive, extrapolative cleaning is a novel approach to cleaning that can be implemented immediately at membrane desalination facilities. Cleaning is conducted before membrane show performance decline with the objective to intervene before biofouling becomes well established. This approach sacrifices elements initially during operations but may result in lower lifecycle costs.

Table 21 summarizes the advantages, limitations, and status of the most promising new techniques. Many show potential for direct membrane disinfection, but demonstrated mixed performance in the studies reviewed herein, particularly for modified polyamide membranes. As noted in the table, many of these technologies are still in the R&D stage.

## 7. Conclusions

In closure, current options to end users of membrane-based desalination systems are limited to chemical or non-chemical pretreatment, on-membrane application of select non-oxidizing biocides which are of limited value, and reactive cleaning. The very nature of polyamide membranes encourages biofouling to some degree as do the membrane elements. Commonly used oxidizing biocides cannot be used on the membrane itself. Both chemical and physical pretreatment methods can suffer from regrowth post treatment. Cleaning after the fact can result in a cycle of more frequent and progressively ineffective cleaning. The nature of a spiral wound element and the polyamide membrane limit the how aggressive the chemical and physical cleaning can be, thereby, limiting the efficiency of the cleaning regimen.

The ultimate objective of research into biocontrol control strategies should involve direct minimization or prevention of biogrowth on the polyamide membrane itself. The most promising techniques to directly minimize biofouling on polyamide membranes discovered during research for this paper are membrane modifications and the use of biocides directly on the membrane. Membrane modifications include surface coating, nanoparticle membranes, and GO membranes which modify the surface properties to discourage biofouling from taking hold. GO membranes have demonstrated good biofouling mitigation and chlorine resistance albeit at marginal performance with respect to standard polyamide and TFN membranes. These are obviously not field techniques, so end users need to rely on membrane researchers and manufacturers to develop these membranes. Promising biocidal preparations, that could be used in the field, include NO donor compounds, DCC, and chlorine dioxide. Some of these techniques are in their infancy while others are commercially available but have not been fully vetted for application with polyamide membranes. Of these compounds, chlorine dioxide is commercially available and has demonstrated excellent biofouling control in other applications; it has the potential address biofilm formation directly on membranes. Questions linger, however, about its compatibility with polyamide membranes, as results of research work are conflicting. The objectives of current and future research should focus on scale-up of fledgling techniques and rigorous membrane compatibility testing of more mature techniques.

## Figures and Tables

**Table 1:** Contact angle and zeta potential as a function of pH for DuPont-FilmTec (Edina, MN, USA) XLE membrane at constant, 10 mM sodium chloride solution. Used with Permission of Elsevier B.V. [40].

pH (Standard Units)	Contact Angle (Degrees)	Zeta Potential (mV)
3	62 ± 2.4	+20.6
5	61 ± 2.8	−12.7
7	55 ± 1.8	−30
9	47 ± 1.4	−38.8
10	45 ± 1.2	−41.5
11	44 ± 0.7	ND

ND: not determined.

**Table 2:** Membrane characteristics as a function of ionic strength of a sodium chloride solution for a DuPont-FilmTec XLE membrane at pH 6.8 ± 0.2. Used with Permission of Elsevier B.V. [40].

Ionic Strength (mM)	Contact Angle * (Degrees)	Interfacial Free Energy (mJ/m^2^)	Interfacial Free Energy of Cohesion (mJ/m^2^)
10	65.6	95.2	−39.2
100	59.6	100.3	−30.2
1000	56.5	102.8	−25.6

* average.

**Table 3:** Oxidizing compounds and their respective reduction potentials. Used with permission of the Engineers’ Society of Western Pennsylvania.

Species	*E°*, Volts	Species	*E°*, Volts
Hydroxyl Radical, •OH	+2.8	Chlorine gas, Cl_2_	+1.346
Ozone, O_3_	+2.076	Dichloramine, NHCs’	+1.34
Peracetic Acid	+1.81	Oxygen, O_2_	+1.23
Hydrogen peroxide, H_2_O_2_	+1.776	Chlorine Dioxide, ClO_2_	+0.954
Hypochlorous Acid, HOCl	+1.482	Hypochlorite Ion, OCl^−^	+0.81
Monochloramine, NH_2_Cl	+1.4	-	-

**Table 4:** Comparison of various chlorine-based oxidizers showing standard reduction potential, available chlorine, and deactivation time for 1-log reduction of *Giardia* cysts at 25 °C. Adapted from [90,91].

Oxidizer	*E°* (V)	Available Chlorine (%)	pH	Deactivation Time (min)
Chlorine Dioxide	+0.95	263	6–9	3.7
Hypochlorous Acid	+1.49	135.2	6	10
Hypochlorous Acid	+1.49	135.2	7	14
Hypochlorite Ion	+0.94	139	8	20
Hypochlorite Ion	+0.94	139	9	29
Monochloramine	+1.40	137.9	6–9	250

**Table 5:** Disinfection credits and CT values in mg-min/L for inactivation of *Giardia* cysts by ozone, as noted by the United States Environmental Protection Agency (USEPA), 1991. [178].

Disinfection Credit (Log Removal)	Temperature (°C)		
	5	15	25
1.0	0.63	0.32	0.16
2.0	1.3	0.63	0.32
3.0	1.9	0.95	0.48

**Table 6:** Disinfection credits and CT values in mg-min/L for inactivation of viruses by ozone, and noted by the USEPA, 1991. [178].

Disinfection Credit (Log Removal)	Temperature (°C)		
	5	15	25
2.0	0.60	0.30	0.15
3.0	0.90	0.50	0.25
4.0	1.2	0.60	0.30

**Table 7:** Disinfection credits and CT values in mg-min/L for inactivation of *Cryptosporidium* by ozone, as noted by the USEPA, 2003. [179].

Disinfection Credit (Log Removal)	Temperature (°C)		
	5	15	25
1.0	16	6.2	2.5
2.0	32	12	4.9
3.0	47	19	7.4

**Table 8:** Comparison of CT values in mg-min/L for various oxidants to achieve 2-log (99%) inactivation of the given organism at 5 °C, as noted by Hoff, USEPA, 1986. ND = not determined. [177].

Organism	Ozone	Free Chlorine	Chloramine	Chlorine Dioxide
	pH 6–7	pH 6–7	pH 8–9	pH 6–7
*E. coli* bacteria	0.02	0.034–0.05	95–180	0.4–0.75
Polio 1 virus	0.1–0.2	1.1–2.5	770–3740	0.2–6.7
Rotavirus	0.006–0.06	0.01–0.05	3806–6476	0.2–2.1
*Giardia lamblia* cysts	0.5–0.6	47–150	ND	ND
*Giardia muris* cysts	1.8–2.0	30–630	ND	7.2–18.5

**Table 9:** UV dosage required to achieve given log inactivation of various microorganisms, as noted by the USEPA, 2003 [223].

Microorganism	UV Dose (mJ/cm^2^)			
	1-log	2-log	3-log	4-log
*E. coli*	1.5	2.8	4.1	5.6
Polio 1	4	8.7	14	21
*Cryptosporidium*	<2	<3	<5	
*Giardia lamblia*	<1			<2

**Table 10:** General ranges of UVT_254_ for various water sources. Used with permission of John Wiley & Sons. [220].

Water Source	UVT_254_
Raw, untreated surface water	<45%
Unfiltered, secondary effluent	45–65%
Tertiary filtered wastewater	55–75%
Drinking water	70–98%

**Table 11:** Comparison of preservative solutions, DBNPA and CMIT/MIT (isothiazolone), with sodium metabisulfite for brackish water, polyamide membranes. Basis: 10,000 m^3^/day plant using 630 standard brackish water, polyamide membranes, 20 m^3^ preservative solution. Reproduced from WST:WS volume 11, issue number 3, pages 343–351, with permission from the copyright holders, IWA Publishing. [204].

Biocide	Dosage, Active (ppm)	Dosage, Product (ppm)	Preservation Time (months)	Relative Product Cost	Relative Cost/month
Sodium metabisulfite	3900	10,000	3	1	1
DBNPA	30	150	3	8.6	0.13
CMIT/MIT	10	71	6	10	0.036

**Table 12:** Mortality of *E. coli* for coated and un-coated polyamide membrane after 1 h of exposure time. 10^9^ CFU/m^2^ incubation level. Adapted from [268]. Used with permission of Elsevier B.V.

Sample	1-Hour Mortality (%)
Uncoated membrane	15 ± 2.5
3 bilayer	99.6 ± 0.03
5 bilayer	99.8 ± 0.02
7 bilayer	99.9 ± 0.01
10 bilayer	100

**Table 13:** Performance of various polyamide (m-phenylenediamine (MPD)–trimesoyl chloride) composite membranes prepared with graphene oxide (GO) as compared to commercially available, polyamide (PA), and thin-film nanotechnology (TFN) membranes. Type: B = brackish water, S = seawater, N = nanofiltration. Adapted from Ali et al. [287]. Used with permission of Elsevier B.V.

Type	GO Concentration (ppm)	GO Addition Procedure	Pressure (bar)	NaCl Concentration (ppm)	Permeability (l/m^2^-h/bar)	Salt Rejection (%)	REF
B	76 in MPD	embedded	15	2000	1.1	99	131
S	1000	on PSf * substrate	55	32,000	0.51	98	133
N	2000 in MPD	embedded	15	2000	1.5	88	136
B	20,000	on PA layer	15.5	2000	0.90	96	134
B	100 in MPD	embedded	15	2000	2.0	98	126
B-PA **	0		15.5	1500	3.4	99.8	CPA7-LD
S-PA **	0		54	32,000	0.94	99.8	SWC6-LD
B-TFN ***	0		15.5	2000	2.9	99.6	LGBW400R
S-TFN ***	0		55	32,000	0.47	99.85	LGSW400R

* Polysulfone, ** Hydranautics commercial polyamide membranes (a Nitto Group Company, Oceanside, CA, USA), *** LG Chem Water Solutions commercial TFN membranes (El Segundo, CA, USA).

**Table 14:** Characteristics and performance of TFC and GO-functionalized TFC membranes from different studies by Ali, et al., Huang et al., and Chae et al. [287,290,292].

Membrane	Contact Angle (°)	Average Surface Roughness (nm)	Zeta Potential (mV)	Thickness of Active Membrane Layer (nm)	Permeability (l/m^2^-h/bar) *	Rejection (%) *
TFC 1	75	84	−21	340	0.58	99.2
GO 2	55	75	−38	270	0.97	99.3
GO 3	50	68	−42	250	1.1	99.4
GO 4	48	60	−44	245	0.99	99.5
TFC 5	64	NA	NA	4560	1.4	98.5
GO 6	55	NA	NA	1930	1.9	98
GO 7	52	NA	NA	4060	1.7	94.5
TFC 8	85	44	NA	NA	2.4	94.1
AGO 9	45	29	NA	NA	2.3	95.3

NA not available; TFC 1: virgin TFC membrane (origin and type not provided) [292]; GO 2: GO-imbedded polyamide via 15 ppm GO in MPD solution [292]; GO 3: GO imbedded polyamide via 38 ppm GO in MPD solution [292]; GO 4: GO imbedded polyamide via 76 ppm GO in MPD solution [292]; TFC 5: hand prepared TFC brackish water membrane [287]; GO 6: GO imbedded polyamide via 100 ppm GO in MPD solution [287]; GO 7: GO imbedded polyamide via 200 ppm GO in MPD solution [287]; TFC 8: DuPont Filmtec XLE (low energy, high permeability, lower rejecting) commercial brackish water membrane [290]; AGO 9; AGO-grafted polyamide membrane [290]; 2000 ppm sodium chloride solution.

**Table 15:** Expected product constituents for optimized chlorine dioxide generators, per 1000 ppm of chlorine dioxide generated. Adapted from [308]. Used with permission of John Wiley & Sons.

Compound	Chlorite-Based Generation (ppm)	Chlorate-Based Generation (ppm)	Electrochemical Generation (ppm)
Chlorine (as hypochlorous acid/hypochlorite ion)	<50,000	NA	NA
Chlorite ion	NA	NA	Without gas-stripping, high concentrations possible
Chlorate ion	based on chlorine concentration	2.5–23	NA
Sulfuric acid	NA	3500	NA
Hydrogen peroxide	NA	120	NA
Perchlorate ion	NA	0.0001	NA

NA = generally not present.

**Table 16:** Expected product constituents for non-optimized chlorine dioxide generators, per 1000 ppm chlorine dioxide generated. Adapted from [308]. Used with permission of John Wiley & Sons.

Compound	Chlorite-Based Generation (ppm)	Chlorate-Based Generation (ppm)	Electrochemical Generation (ppm)
Chlorine (as hypochlorous acid/hypochlorite ion)	>50,000	NA	NA
Chlorite ion	variable	NA	Without gas-stripping, high concentrations possible
Chlorate ion	based on chlorine concentration	9590	variable
Sulfuric acid	NA	8200	NA
Hydrogen peroxide	NA	25	NA
Perchlorate ion	NA	0.007	variable

NA = generally not present.

**Table 17:** Product composition for a commercial chlorine dioxide product generated using sodium chlorate via Equation (28) [311]; does not include side reaction or decomposition compounds.

Compound	Type	Product (wt%)
Chlorine Dioxide	Product	17.8
Sulfuric Acid	Reactant	56.4
Sodium Chlorate	Reactant	1.4
Hydrogen Peroxide	Reactant	1.4
Sodium Sulfate	Product	18.7
Oxygen	Product	4.3

**Table 18:** Performance of a new Toray TM820H-400 (Toray, Japan) seawater membrane and when exposed to 100 ppm-hours of chlorine dioxide (5-h exposure at 20 ppm) at 49 bar and 25 °C. Testing pH for the new membrane was not disclosed. Permission has been granted by the author and publisher (Balaban Desalination Publications), as adapted from *Desalination and Water Treatment* (2012) 45(1–3) 84–90. [214].

Condition of ClO_2_ Exposure	Permeate Flux (l/m^2^-h)	Salt Rejection (%)
New Membrane—no exposure	439.491	99.46
pH = 4	588.265	98.47
pH = 7	587.462	99.39
pH = 9	779.308	97.97

**Table 19:** Concentration of chlorine dioxide and the by-product, chlorite, in 1000 mL of seawater before and after addition of 0.40 ppm chlorine dioxide. Chlorine dioxide generated using chlorine and sodium chlorite (Equation (23)). Conditions: temperature = 27 °C, pH = 7.81, conductivity = 42.4 mS/cm, turbidity = 1.93 NTU. Adapted from [369]. Used with permission of the American Water Works Association.

Compound	Before Addition	Immediately After Addition	After 30 Minutes of Agitation at 40 rpm
Chlorine (Cl_2_)	BDL	0.053 ppm	BDL
Chlorine dioxide (ClO_2_)	BDL	0.400 ppm	0.141 ppm
Chlorite (ClO_2_^-^)	BDL	0.267 ppm	1.82 ppm

BDL = below detection limits.

**Table 20:** Summary of the salient advantages and limitations of currently employed, conventional biofouling control techniques for polyamide membrane systems.

Class	Advantages	Limitations
**Oxidizers**		
Chlorine	Easy to use Good bio control Carries residual disinfectant	Polyamide degradation DBP formation
Chloramine	Carries residual disinfectant Lingers in distribution system	Polyamide degradation (with metals as catalyst) Slow reaction kinetics
Ozone	Very good bio control	Quick dissipation Polyamide degradation
**Non-Oxidizers**		
Dbnpa	Good polyamide compatibility	Short half-life at pH > 7 Poor disinfection (better at keeping clean membranes clean) Off-line use for potable applications
Isothiazolones	Good polyamide compatibility Best used for long term storage	Hazardous chemical—aquatic toxicity Little disinfection (better at keeping clean membranes clean) Slow acting Off-line use for potable applications
Sodium Bisulfite	Good polyamide compatibility Best for preventing aerobic bacteria growth Generally used for long term storage	Can promote anaerobic biofouling Does not provide membrane disinfection
**Non-Chemical**		
Ultraviolet Radiation	Good bio control	Bacteria subject to photoreactivation or dark repair Carries no residual Equipment subject to fouling/scaling Capital intensive
Cleaning	Easy to employ Possibility to restore membrane performance	Typically applied after biofouling has reached plateau phase Does not disinfect membrane

**Table 21:** Summary of the salient advantages and limitations as well as development status for the most promising new techniques for polyamide membrane biofouling control.

Technique	Advantages	Limitations	Development Status
**Membrane Modification**			
Coatings	Surface modification to discourage bio-adhesion	Mechanical and chemical instability Mixed results for biocontrol and membrane performance	R&D
Nanoparticles	Use of biostatic nanoparticles to discourage bio-adhesion	Mixed results for biocontrol Deleterious effects on membrane performance	R&D/Commercial
Graphene Oxide	Demonstrated resistance to biofouling Demonstrated resistance to chlorine Membrane specific flux improvement with similar salt rejections to polyamide membranes	Mixed results reported for GO membrane performance Possible scale-up issues	Early bench-scale R&D
**Chemical**			
Chlorine Dioxide	Excellent biocide to penetrate EPS	Mixed results on polyamide membrane compatibility Forms chlorite and chlorate oxidative DBPs Capital intensive—on-site generation required Difficulty in measuring residual USEPA and OSHA limits on residual and exposure	Commercial (compatibility studies in R&D phase)
DCC	Some polyamide compatibility Superior inactivation of *Pseudomonas aeruginosa* to chlorine	Lacking regulatory approval as a biocide Expensive	Bench-scale R&D
NO Donor Compounds	Toxic to bacteria Disperses EPS	Lacking regulatory approval as a biocide Resistance of some bacterial populations	R&D
AOP	Effective at oxidizing chemical and biological oxygen demand	Limited to membrane pretreatment	Commercial/wastewater reuse applications
Extrapolative Cleaning	Cleaning early in conditioning/adhesion phases of biofouling development	Does not disinfect membrane Frequent membrane autopsies	Infrequently practiced

## Abbreviations

AFM	Atomic force microscopy
AgNP	Silver nanoparticle
AGO	Azide-functionalized graphene oxide
AI	Autoinducers
AOC	Assimilable organic carbon
AOP	Advanced oxidation process
ATP	Adenosine triphosphate
BAC	Biological activated carbon
BCDMH	1-Bromo-3-chloro-5,5-dimethylhydantoin
BFR	Biofilm formation rate
BSA	Bovine serum albumin
CA	Cellulose acetate
CFU	Colony forming units
CMIT	5-Chloro-2-methyl-4-isothiazolin-3-one
CT	Oxidant concentration times contact time
DBNPA	2,2-Dibromo-3-nitriopropionamide
DBP	Disinfection byproduct
DCC	Dichloroisocyanurate
DNA	Deoxyribonucleic acid
DO-HS	Direct osmosis high salinity
DOM	Dissolved organic matter
DPD	N,N-diethyl-*p*-phenylenediamine
EBCT	Empty bed contact time
EPS	Extracellular polymeric substances
FTIR	Fourier transform infrared spectroscopy
GO	Graphene oxide
GWRS	Ground water replenishment system
HAA	Halo-acetic acid
HPC	Heterotrophic plate counts
HRL	Health reference level
HRP	Horseradish peroxidase
LGB	Lissamine Green B
LP	Low pressure
LPHO	Low pressure high output
MAHMA NONOate	6-(2-Hydroxy-1-methyl-2-nitrosohydrezion)-N-methyl-α-hexanamine
MCL	Maximum contaminant level
MCLG	Maximum contaminant level goal
MIT	2-Methyl-4-isothiazolin-3-one
MP	Medium pressure
MPD	*m*-Phenylenediamine
MRDL	Maximum residual disinfection limit
NdP	Normalized differential pressure
NF	Nanofiltration
NO	Nitric oxide
NOM	Natural organic matter
NPF	Normalized permeate flow
NSP	Normalized salt passage
OCC	Offline chemical cleaning
OMC	Online mechanical cleaning
OMCC	Online mechanical/chemical cleaning
ORP	Oxidation reduction potential
OSHA	Occupational Health and Safety Agency
PA	Polyamide
PAA	Peracetic acid
PEL	Permissible exposure limit
PSf	Polysulfone
RBS	Rutherford backscattering spectrometry
RNA	Ribonucleic acid
RO	Reverse osmosis
TDC	Total direct counts
TFC	Thin film composite
TFN	Thin film nanocomposite
THM	Trihalomethane
TOB	Tobramycin
TOC	Total organic carbon
USEPA	United States Environmental Protection Agency
UV	Ultraviolet
UVT_254_	Ultraviolet transmittance at 254 nm
ZIF-8	Zeolitic imidazolate framework-8
